# Microfluidic reactors for advancing the MS analysis of fast biological responses

**DOI:** 10.1038/s41378-019-0048-3

**Published:** 2019-02-11

**Authors:** Iulia M. Lazar, Jingren Deng, Mark A. Stremler, Shreya Ahuja

**Affiliations:** 1 Department of Biological Sciences, Virginia Tech, 1981 Kraft Drive, Blacksburg, VA 24061 USA; 2 Virginia Tech Carilion School of Medicine, Virginia Tech, 2 Riverside Circle, Roanoke, VA 24016 USA; 3 Department of Mechanical Engineering, Virginia Tech, 780 Drillfield Drive, Room 333P, Blacksburg, VA 24061 USA

**Keywords:** Microfluidics, Environmental, health and safety issues

## Abstract

The response of cells to physical or chemical stimuli is complex, unfolding on time-scales from seconds to days, with or without de novo protein synthesis, and involving signaling processes that are transient or sustained. By combining the technology of microfluidics that supports fast and precise execution of a variety of cell handling operations, with that of mass spectrometry detection that facilitates an accurate and complex characterization of the protein complement of cells, in this work, we developed a platform that supports (near) real-time sampling and proteome-level capturing of cellular responses to a perturbation such as treatment with mitogens. The geometric design of the chip supports three critical features: (a) capture of a sufficient number of cells to meet the detection limit requirements of mass spectrometry instrumentation, (b) fluid delivery for uniform stimulation of the resident cells, and (c) fast cell recovery, lysis and processing for accurate sampling of time-sensitive cellular responses to a stimulus. COMSOL simulations and microscopy were used to predict and evaluate the flow behavior inside the microfluidic device. Proteomic analysis of the cellular extracts generated by the chip experiments revealed that the identified proteins were representative of all cellular locations, exosomes, and major biological processes related to proliferation and signaling, demonstrating that the device holds promising potential for integration into complex lab-on-chip work-flows that address systems biology questions. The applicability of the chips to study time-sensitive cellular responses is discussed in terms of technological challenges and biological relevance.

## Microfluidics: Weighing the benefits of real-time cellular monitoring

Chip-scale devices that quickly deliver proteins expressed by cells to mass spectrometers may bring quantitative insights into the early stages of cancer. Many proteins generated by cells during signaling events are transient and present in numbers too small to be detected by typical analytical instruments. Iulia Lazar and colleagues from Virginia Tech in Blacksburg, United States have developed a microfluidic system that improves the capture of these biomolecules by exposing cells, held in high-capacity chambers, to a crosswise flow of stimulating agents. This setup yielded faster and more accurate mass spectrometry analysis of the cellular protein content than the systems that delivered agents lengthwise along the sample chambers. Experiments with breast cancer cells enabled the team to identify hundreds of proteins involved in growth and division processes in the few minutes following exposure to mitosis-triggering substances.

## Introduction

Cells are the elementary unit of life and they are constantly exposed to various stimuli from the external microenvironment. Signals transduced into the cell result in changes in the composition of the cellular contents, such as gene and protein expression and post-translational modifications (PTMs)^1^. The analysis of intracellular proteins and their modifications is playing an increasingly important role in understanding the cell signaling regulatory mechanisms, in biomarker characterization, and/or in drug target discovery^2,3^. Over the past decades, mass spectrometry (MS) has emerged as one of the most powerful and widely used technologies for the characterization of cellular proteins, due to its high sensitivity, specificity, and feasibility of coupling with other analytical technologies such as HPLC. Unfortunately, despite the fact that MS analysis can be highly automated and rapid, conventional sample preparation methods are labor-intensive, time-consuming, and most importantly, not prone for supporting real-time explorations of cell behavior. Therefore, studying certain biological processes and low-level and/or transient cell responses to external stimuli represents a challenge to many studies. In addition, the need for various lytic agents, such as SDS or Triton X-100^4^ to disrupt the cell membrane, results in the contamination of the sample, additional processing steps and possibly hampered detection.

In recent years, microfluidics has gained dramatic interest due to the novel, high-throughput analysis approaches that it can provide to molecular biology, clinical medicine, and biomedical sciences^5–7^. The main advantages presented by microfluidics include integration, miniaturization, automation, and low sample and reagent consumption. Microchip devices have been developed for a variety of applications, including the study of cellular responses to a variety of stresses, intra-/extra-cellular signaling, cell–cell interactions, immunoassays, and even tissue regeneration^8–12^. For studies that involve the analysis of the cellular content, microfluidic-based cell lysis methods have been proposed^13–24^. Particular emphasis has been placed on advancing platforms for nucleic acid extraction^25,26^ and single-cell analysis, to enable sequencing efforts and to assess the variability associated with the behavior of single cells^27–29^. There are, however, a number of limitations associated with this strategy that stem from the very concept of isolating the cells from the bulk. This is particularly important in the context of biological interpretation of results, as isolated cells lose the spatial context of a tissue (or culture) and respond differently to certain stimuli. Moreover, the technical errors that are associated with the handling and analysis of limited amounts of material lead to noise and artifactual results that often are misinterpreted as biological variability^29^. It has been shown, for example, that stochastic ERK (extracellular signal-regulated kinase) activity pulse and cell proliferation were cell-density dependent^30^. As a result, studies that involve cell signaling and functional assays in a physiologically relevant environment will be better served by platforms that accommodate population of cells that mimic the conventional cell or tissue culture model^29,31–33^. It has been also proposed that isogenic cell lines formed by subcloning single cells would represent good proxies to single cells^29^.

When entering the realm of the proteome, tools for single molecule amplification, such as PCR, do not exist. Many proteins with important roles in cell regulation are present in very low copy numbers (<1,000 copies/cell), and their abundance in a cell is far below the detection limits of commonly used analytical methods, including mass spectrometry. Moreover, in a typical proteomic experiment, after cell stimulation with growth factors in a culture chamber, cell processing steps such as detachment from the culture flask, rinsing, and centrifugation, expose the cells to chemical and mechanical stresses, and introduce a time-delay of at least 30–40 min between the cell stimulation and cell sampling events. As many cellular responses to stimuli are fast and/or transient, conventional cell culture and sample handling approaches totally decouple the biological event of interest from the product that is being analyzed. The onset of biological responses that occur within minutes of stimulation are therefore completely missed if the sampling strategy of cells (and/or the sampling frequency) is too slow, or if it introduces changes in the chemical composition of the cellular environment. To address this challenge, in this work, we devised a microfluidic chip that facilitates (near) real-time sampling of cell responses to a stimulant for downstream MS analysis and proteomic profiling. The time-delay and interference from various processing steps that are integral to typical cell handling protocols, that alter the accurate characterization of the biological process under study, are eliminated. Signaling events are frozen within minutes of stimulation. We evaluated the performance of the platform for effectiveness in cell handling, stimulation, and cell processing for MS detection. We demonstrate its applicability to enabling proteomic profiling of cells subjected to various treatments, and we discuss the utility of the device in the context of addressing systems-level biology questions that involve the evaluation of time-sensitive cellular responses.

## Results

### Microfluidic chip design

**Fig. 1:**
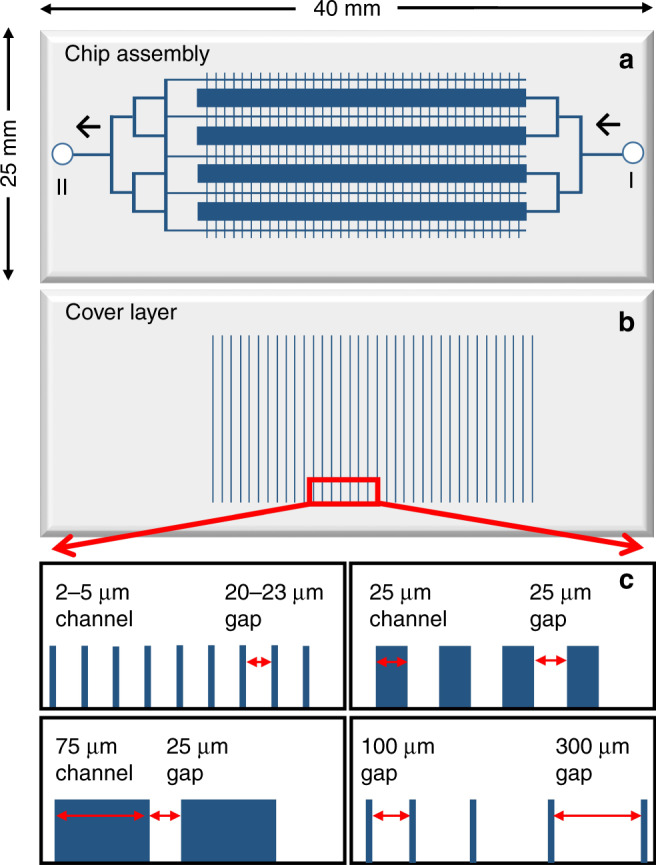
Multiplexed microfluidic chip with axial delivery of cells and cell stimulation medium. **a** Schematic diagram of the microfluidic chip, with arrows indicating the direction of cell and fluid delivery, from inlet (I) to outlet (II); **b** Cover slip with filter channels; **c** Layout and dimensions (prior to etching) of the filter channels

To address diverse needs of biological research, a number of different chips, featuring two main microfluidic designs, were developed: one with axial/uni-directional delivery of cells and cell culture medium that facilitated multiplexing, and one with axial loading of cells and transversal delivery of the stimulation medium for applications that require a quick exchange of conditions in the cell culture chamber. The chips were fabricated from glass substrates to minimize protein losses due to adsorption on the channel walls, and to facilitate easy sterilization and re-usability. A 4-plex design was initially envisioned, to enable the uniform capture and stimulation of a large number of cells (Fig. 1a). The chip comprised 4 cell capture chambers placed in an arrangement that enabled uniform cell loading from a common inlet reservoir (I). The drawing dimensions of each chamber were L = 10 mm and W = 500 µm, generating a corresponding volume of 250 nL for a 50 µm etch depth. For cells of an average size of 15 μm in diameter, the theoretical loading capacity of a fully packed chamber was estimated to be ~100,000 cells (i.e., for a maximum possible, regular packing density of 74% with incompressible and non-deformable spheres of 15 µm in diameter). The cells, however, are not uniform-sized non-deformable spheres and cannot be packed regularly to achieve maximum packing density. Therefore, the actual number of cells in a 250 nL chamber is expected to be lower. Experimental observations in our laboratory led to estimates of (20–40) × 10^6^ cells in 100 µL packed cell volumes (PCV). As such cell counts typically generated ~5–10 mg protein per 100 µL PCV, based on Bradford assay measurements, one 250 nL microfluidic cell chamber would be expected to produce up to 12–25 µg protein extract from 50,000–100,000 cells. Modern mass spectrometers enable the detection of hundreds-to-thousands of proteins from only a few µg of proteomic sample. Therefore, unless a large number of proteomic experiments are to be conducted with the proteins extracted from the cells processed on the chip, even a chip with a single, smaller chamber would provide a sufficient amount of material for downstream MS analysis. The four chambers were placed apart at an equal distance, and connected by symmetrically branched channels (feed channels) to the inlet port (I) that was used to load the cells and deliver the culture medium. Five culture medium collection channels (waste channels), placed on both sides of the four chambers, were connected to a common outlet reservoir (II). Shallow, transversal channels (~1.5–4 µm deep) were used as a filter to allow for the retention of cells and the passage and removal of the medium (Fig. 1b). The cell chamber and feed/waste channels were etched in the chip substrate, while the transversal filter in the cover layer. To achieve efficient removal of the cell culture medium during cell loading, uniform distribution of cells in the cell chambers, and uniform cell stimulation with growth factors, four layouts of the transversal filter element were designed and tested. These layouts encompassed 2 µm, 5 µm, 25 µm and 75 µm wide channels placed 25 µm, 25 µm, 50 µm, and 100 µm apart, respectively. The goal was to maximize the areas through which the medium is removed, minimize the back pressures during cell loading and stimulation, and prevent the cells from escaping the cell chambers during cell handling operations. Alternatively, 2 µm channels were separated by progressively smaller gaps (from 300 µm to 100 µm), as progressing from the inlet to the outlet of the cell chamber, to allow for more efficient removal of the medium from the tail once the chamber becomes filled with cells (Fig. 1c). Due to isotropic etching to a depth of 1.5–4 µm, the width of the filter channel elements became 6–10 µm wider than the width on the photomask drawing. For the flow rates that were used in this work, all designs enabled trouble-free removal of the cell medium during cell loading, as well as uniform cell loading and distribution in the cell chambers. The etch depth, however, which was the critical dimension on the chip, had a larger impact on the operation of the device. Shallow filters (<2 µm) minimized cell penetration and prevented the cells from breaking through. However, the back pressure increased substantially for such filters, and impeded easy delivery of the stimulation medium to the cell chambers. Deeper filters (3–4 µm), on the other hand, while facilitated trouble-free delivery of the stimulation medium, allowed for some cells to squeeze through the filter channels and be eliminated to waste. Altogether, to ensure the quality of the stimulation step, layouts comprising 25 µm and 75 µm wide transversal channels placed 50 µm or 100 µm apart (Fig. 1c, upper right and lower left panels), etched to a depth of 3–4 µm, proved to be the most practical choice for the experiments performed in this work. It is also envisaged that the filter layout in which the gaps become progressively smaller from the inlet to outlet (Fig. 1c, lower right panel) would benefit the loading of cells in chips with longer cell chambers than the ones used in this study.

**Fig. 2:**
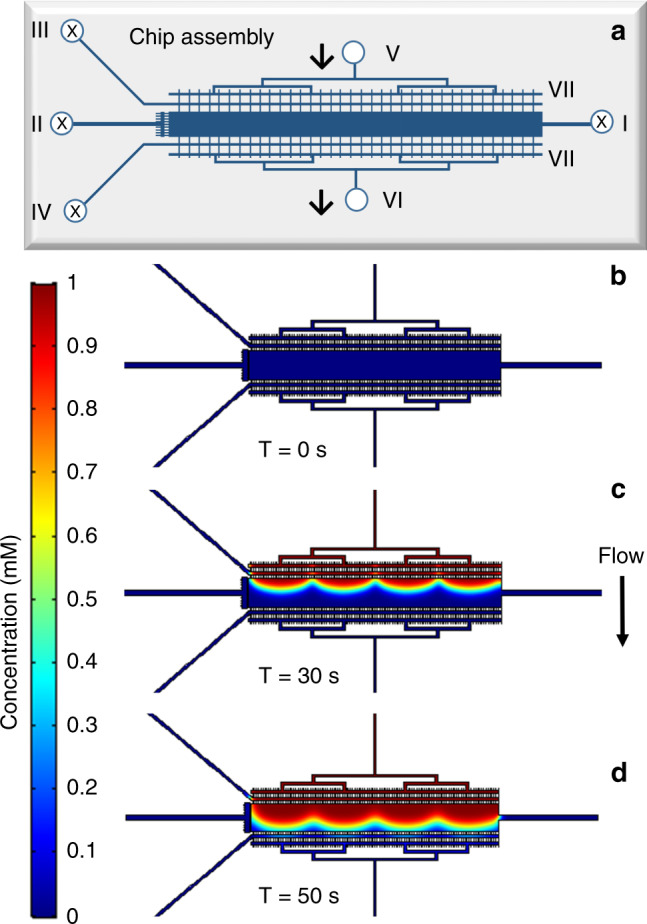
Microfluidic chip with axial cell loading and transversal delivery of the stimulation medium. **a** Schematic diagram of the microfluidic chip, with arrows indicating the direction of the stimulation medium from inlet (V) to outlet (VI); the cell inlet (I) and medium outlet (II), (III) and (IV) are closed during transversal delivery of the stimulation medium; the additional flow distribution channel is denoted by (VII). **b**–**d** COMSOL simulation of the transversal EGF infusion process at 1 µL/min. The cell compartment dimensions were L = 10 mm, W = 1,000 μm, D = 60 μm, and V = 600 nL. The concentration profile of the EGF solution is presented at three time-points: **b** 0 s; **c** 30 s; **d** 50 s

The 2nd microfluidic design encompassed only one cell chamber (Fig. 2a), but supported enhanced flexibility in handling the fluids on the chip. The drawing width of the cell compartment was either W = 500 µm or W = 1,000 μm, and the length L = 10 mm, resulting in a chamber volume of 250 nL or 500 nL, respectively, for an etch depth of 50 µm. Unlike the first, multiplexed design, this device benefitted from multiple inlets (I and V) and outlets (II, III, IV, and VI) for cell loading and fluid delivery. Equidistantly distributed feed/waste and filter channels, relative to the inlet and outlet ports (V) and (VI), enabled uniform medium delivery for transversal cell stimulation. An additional distribution channel (VII), placed between the waste channels and the inlet/outlet ports (V/VI), was incorporated in the chip to further help the delivery of a homogeneous stimulation flow to the cell chambers and prevent cell leakage in the port reservoirs (V/VI).

### COMSOL simulation of cell culture medium infusion in the chip

**Fig. 3:**
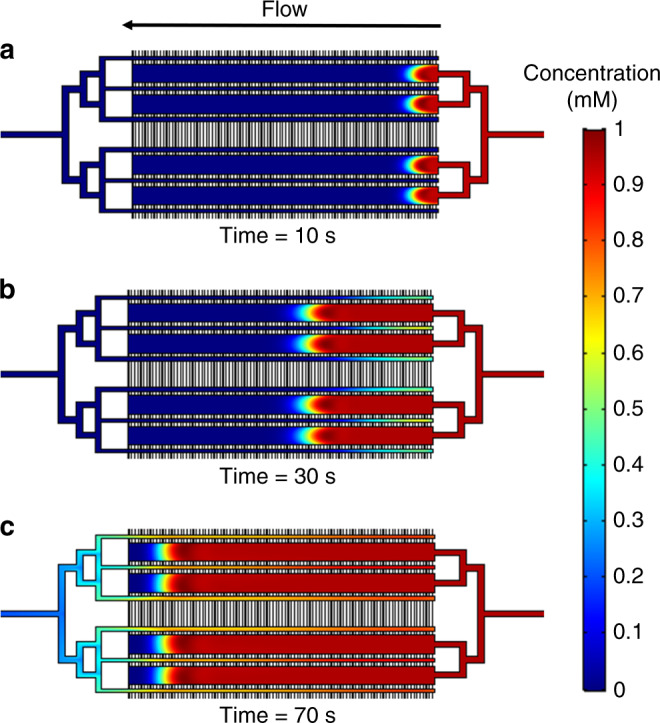
COMSOL simulation of the axial EGF infusing process in a multiplexed chip design. The EGF solution was infused from right to left, at 1 µL/min. The cell compartment dimensions were L = 10 mm, W = 600 μm, D = 50 μm, and V = 300 nL. Red and blue indicate high and low concentrations, respectively. The concentration profile of the EGF solution is presented at three time-points: **a** 10 s; **b** 30 s; **c** 70 s

To test whether the microfluidic layout and flow distribution channels are adequate for uniform delivery of the culture medium to the cell chambers in the singlet and multiplexed chip designs, time-dependent concentration profiles were generated for axial and transversal infusion of the medium using the COMSOL Multiphysics Simulation package. The cell compartment dimensions were chosen to match the actual dimensions of the experimental chips. The transversal filter channels were given a cross-section of W = 50 µm × D = 3 µm, which was considered illustrative of the 25 µm or 75 µm wide and ~3–4 µm deep filter channels that were commonly used in this work. As the cell chamber was etched in the chip substrate and the filter channels in the cover, the filter channels laid on the top of the chambers in the actual experimental chip. The models were first constructed in 2-D, and then converted into 3-D. For simplicity, a uniform depth of 50 μm was assigned to all filter channels and the width of the filters was adjusted to 3 µm to maintain the cross-section of the filter and the chamber filling velocity unchanged. The model was built with the following settings: incompressible laminar flow with no slip on the boundary, water as the material, average mesh density, and all coefficients with default values. The concentration of the stimulant solution (i.e., EGF for SKBR3 cells) was set at 1 unit concentration and the flow rate at 1 µL/min. For a multiplexed chip design, the concentration profiles at three-time points, 10 s, 30 s, and 70 s (i.e., after entering, close-to-middle, and before leaving the cell compartments), are shown in Fig. 3.

Blue and red indicate low and high EGF concentration, respectively. The results show that the four parallel cell chambers have the same flow profile at all time-points, that all cell compartments can be stimulated simultaneously, and, as a result, that the multiplexed chip design has an effective layout for achieving increased cell handling capacity. As shown in the following sections, the multiplexed arrangement with symmetrically-distributed inlet channels also enabled uniform loading and distribution of a homogeneous slurry of cells. However, one obvious drawback of this design is that the infusion is relatively slow along the axial dimension, leading to uneven EGF exposure of cells at different locations, especially toward the end of the chamber that can harbor a dead corner. This was also evidenced by the parabolic flow profile that gradually became flattened towards the end of the chamber (Fig. 3b, c). To accelerate the infusion process and facilitate an even distribution of the EGF stimulant in the cell culture compartment, the second design enabled transversal delivery of the culture medium and stimulant. The results of the simulation for a chamber with W = 1,000 µm are provided in Fig. 2b–d, with the concentration profiles shown after 0 s, 30 s, and 50 s of infusion (i.e., before entering, after entering, and before leaving the cell compartment). The distribution of the EGF solution through hundreds of filter channels greatly facilitated an even distribution of EGF along the chamber length. In both simulations, t = 0 s was the time when the stimulant flow was turned on at the inlets (I) or (V). The flow front reached the cell chamber just before ~10 s in the 1st design and before ~30 s in the 2nd design. For the same flow rate of 1 µL/min, the filling time of the multiplexed chip (4 × 300 nL) and of the 600 nL singlet was, as expected, slightly over ~60 s and ~30 s, respectively. A 250 nL singlet would fill in only ~15 s. The input flow rate of 1 µL/min that was used in the model was a conservative choice for both designs, to minimize the pressure to which the cells would be exposed. Based on the behavior of cells, however, higher flow rates would be tolerated^32^ for achieving fast a uniform composition in the cell compartment. Video simulation files for the axial and transversal infusion of the culture medium through the two microreactor designs are provided in the supplementary material.

### Flow visualization on the chip

**Fig. 4:**
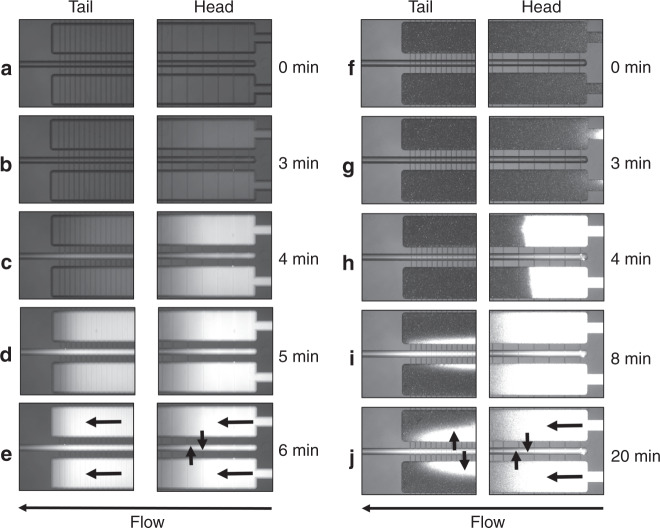
Visualization of the fluid flow with fluorescent rhodamine B dye at the head/tail sections of the multiplexed microfluidic chip. The rhodamine B solution (26 µM) was pumped through the chip at a flow rate of 2 µL/min. The cell compartment dimensions were L = 10 mm, W = 650 μm, D = 55 μm, and V = 360 nL. Bright areas indicate high rhodamine B concentration. **a**–**e** Empty chip; **f**–**j** Chip filled with latex beads (~11 µm). The arrows indicate the direction of the fluid flow in the cell compartment and removal through the filter channels (**e**-head/tail and **j**-head), and back-diffusion from the waste channel into the cell compartment (**j**-tail)

To verify experimentally the utility of the slow-filling axial chip, in which the cells are exposed to a gradient rather than a constant concentration of stimulant, the flow through the multiplexed design with 10 mm long cell reactors was visualized with rhodamine B (26 μM in basic NH_4_HCO_3_ solution, 50 mM). A chip with a filter layout with progressively closer filter channels towards the cell chamber end was used, to facilitate the removal of the culture medium from the chamber tail upon filling with cells. The filling flow rate was also increased to compensate for the slightly larger dimensions of the experimental cell compartments (L = 10 mm, W = 650 μm, D = 55 μm, V = 360 nL) than in the simulation. First, an empty chip was filled with NH_4_HCO_3_ buffer, and rhodamine solution was infused by a syringe pump at 2 μL/min. The filling of the chip was monitored under a microscope, and the fluorescence intensity was recorded in two areas, at the head and the tail of the chamber (Fig. 4a–e). Similarly to the COMSOL simulation, a uniform filling of all chambers was observed, the rhodamine solution reaching the cell chamber head after ~3 min (Fig. 4b) and the tail after ~4 min (Fig. 4c), but a uniform fluorescence along the chamber length was not observable only after an extra 2–3 min (Fig. 4d, e). The 3 min delay to reach the chamber was caused by the dead volume of the tubing that connected the chip to the syringe pump. To mimic the scenario of a cell-packed chip, a 2^nd^ experiment was conducted with a chip filled with latex beads of 10–11 µm in diameter, roughly the same size as the SKBR3 cells (Fig. 4f–j). As in the case of the empty chip, the rhodamine solution reached the cell chamber after ~ 3 min (Fig. 4g), but it was clearly more difficult to infuse uniformly the rhodamine through the fully packed chamber. Even after 8–20 min (Fig. 4i, j), the rhodamine solution could not flood the tail. The observed fluorescence at the tail was due to rhodamine B diffusing back in the cell compartment from the waste channel, after leaking out at the head through the shallow perpendicular filters. We must note, however, that while rhodamine B is a good tracer dye for monitoring fluid transport, it does adsorb on polymeric surfaces, as also evidenced by the bright latex beads at the inlet of the chamber, and this effect much exacerbated the concentration gradient within the cell chamber. Nevertheless, the results underscore the limitations of the axial infusion chip for experimental applications that involve a uniform, time-sensitive cell stimulation step, and stress the need for applying the stimuli in a transversal fashion, as proposed in the 2nd chip design (Fig. 2).

### Cell handling on the chip: loading, stimulation, and lysis

**Fig. 5:**
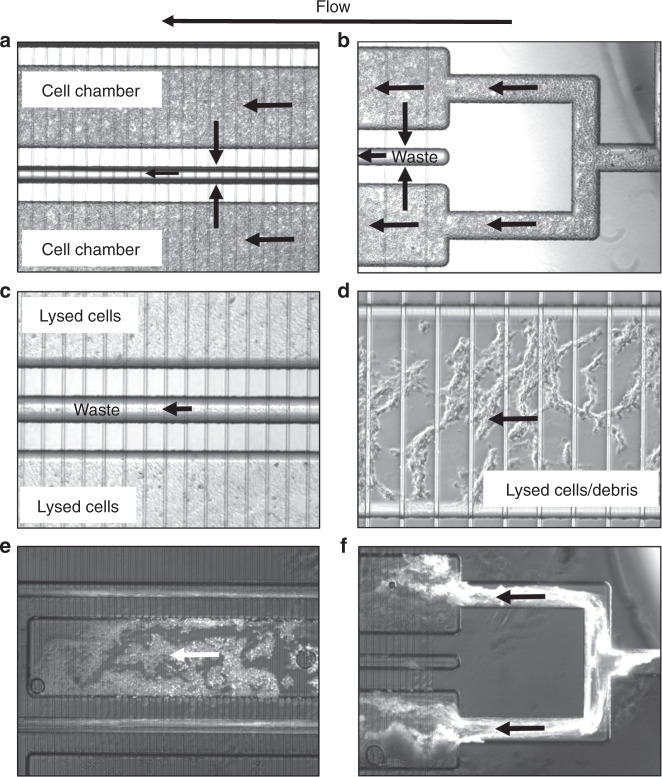
Cell processing on the multiplexed chip. **a** SKBR3 cells, high-density packing in the cell compartment; **b** SKBR3 cells in the inlet area; **c** SKBR3 cell lysis by axial application of the electrical field (2,000 V/cm) through high-density packed cells; **d** SKBR3 cell lysis by axial application of the electrical field (2,000 V/cm) in medium-density packed cells; **e** Lysed HBEC-5i endothelial cells/debris visualized by adsorbed rhodamine B; **f** Morphology of lysed HBEC-5i cells/debris visualized by adsorbed rhodamine B in the cell compartment inlet area. The filter dimensions were W = 12 µm/D = 3 µm for (**a**–**d**) and W = 85 µ/D = 3 µm for (**e**, **f**)

**Fig. 6:**
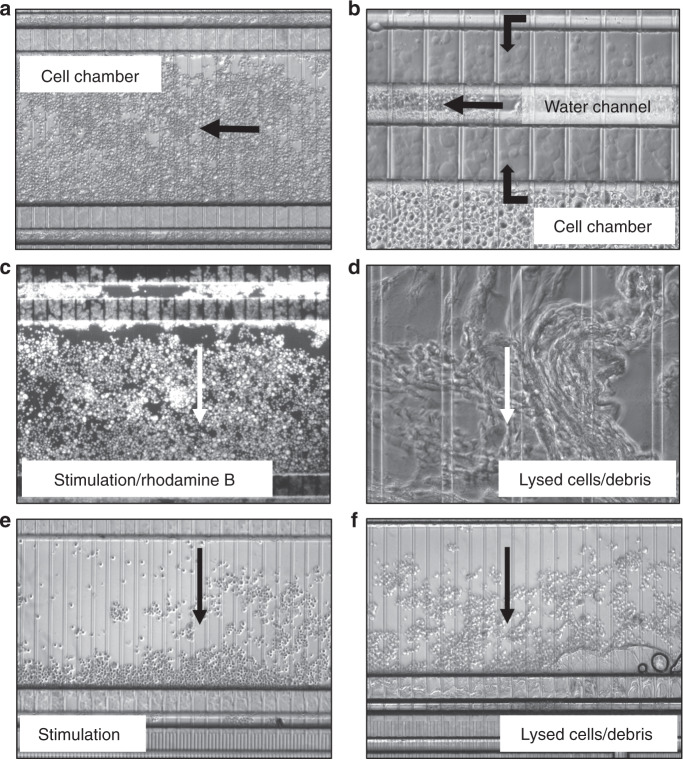
HMC3 microglia cell processing on a chip with transversal delivery of the cell stimulation solution. **a** Cell compartment, medium-density packing; **b** Cell penetration in the filter area; **c** Transversal delivery (~1 min) of a rhodamine B solution (26 µM) in hypotonic solution (NH_4_HCO_3_10 mM/CH_3_CN, 9:1 v/v) for visualizing uniform delivery along the cell chamber length (note also the fluorescent cells that penetrated the filter and leaked in the waste channel, and that adsorbed the dye on their surface); **d** Cell lysis by transversal application of the electrical field (2,000 V/cm) through medium-density packed cells, perfused with hypotonic solution; **e** Transversal delivery of a hypotonic solution through a low-density packed channel; **f** Cell lysis by transversal application of the electrical field (2,000 V/cm) through low-density packed cells, perfused with hypotonic solution. Note in (**c**) and (**e**) the cell packing effect of the transversally delivered solution (from top to bottom), in medium and low-density packed cell compartments. The filter dimensions were W = 85 µm/D = 3 µm

Prior to loading the cells, the chips were filled with PBS to eliminate the air from the cell compartments and the interconnecting channels. The cells, either in PBS or culture medium, were loaded in both microfluidic chips from port (I). The cell density in the loading buffer was critical. At low densities, cell loading was very slow and un-efficient, while at high-density, partial cell lysis and channel clogging was observed. The best conditions involved the preparation of a cell “slurry,” of PCV/medium composition of 1:1 v/v, that was loaded as a block, by gently dispensing the cells in one shot, in the cell compartment. During loading, the cell medium (a few µL) was leaked through the filter channels to ports (II), (III), (IV), (V) and (VI), and collected at the outlet of the tubing connected to these ports. In the transversal stimulation chip, after loading the cells, the tubing connected to ports (I), (II), (III), and (IV) was sealed either with stainless steel rods or with flea wire clips to prevent the loss of medium while infusing from (V) to (VI) during the stimulation step. Figure 5a, b show two cell compartments and the inlet of the multiplexed design, demonstrating the ability to pack the cells uniformly, at high-density. As mammalian cells do not have a rigid cell wall, some cells were able, however, to squeeze into the filter channels and escape in the waste collection channels (see also Fig. 6, in particular the enlarged view of the filter in panel 6b). This resulted in increased back pressure during operation, lysis of some cells, clumping, and potential chip blockage. While this was not always the case, the best way to avoid clogging was to not fully pack the cell compartments, but rather settle for a medium-density packing, such as shown in Fig. 6a. In this case, even if some cells entered the filters, blockage was minimized.

Cells loaded on the chip were further exposed to maintenance medium, infused slowly at <1 µL/min, to prevent disturbing the cell bed. Cell stimulation was performed from ports (I) to (II) in the axial design, and from (V) to (VI) in the transversal design. As expected, transversal infusion of the fluorescent Rhodamine B dye (26 µM) revealed uniform distribution of the stimulation solution along the cell compartment (Fig. 6c). Much higher flow rates are not recommended for infusion, as the pressure exerted on the cells displaces them to the outlet side of the chamber. This was clearly observable at low cell density (Fig. 6e). With axial infusion, however, there was a time-delay until the stimulant reached a homogeneous level in the entire compartment, and, as visualized even with an empty chip (Fig. 4a–e), the mild gradient appeared to persist during the first minutes of infusion. Therefore, based on the downstream biological questions that are pursued with the device, the axial infusion chip would benefit projects focused on studying slow cellular responses to a stimulation, that may occur over hours or days and that would be consequently forgiving of the initial gradient of the stimulant, while the transversal chip would meet the needs of studies that tackle the outcome of fast responses induced in the cell.

Cell lysis was performed on-chip in electrical fields, and in various lysis buffers on- and off-chip. For electrical lysis, the voltage was applied between ports (I) and (II) in the axial stimulation chip, and between ports (V) and (VI) in the transversal chip. The distance between the two electrodes in both designs was roughly the same (2 cm), however, due to the complex chip design, the presence of the large cell chambers, the various interconnecting channels and the shallow filter channels, the field strength in the cell compartments was low in strength, and not uniform. Moreover, the presence of high concentration salts in the culture medium resulted in high electrical currents, Joule heating and bubble generation. DMEM, for example, has at least 6 major salt components with concentration ranging from 0.8 mM to 110 mM. Cell lysis was achievable, however, not uniformly and not instantaneously, but rather over a period of 3–5 min. Figure 5c, d display the SKBR3 cell lysis products in axial chips, in high- or medium-density packed chambers, respectively. The tightly packed chambers (5c) produced a cell “mush” difficult to perfuse with protease and phosphatase inhibitors. The cell debris that was produced when using such lysis conditions was observable in the more loosely packed chambers (see 5d at enhanced magnification). Infusion of rhodamine B from the inlet port (I), and its retention on various lysed cell fragments, highlights the morphology of the debris for HBEC-5i cells (Fig. 5e, f). The release of the cell content rendered the lysis process even more difficult in tightly packed cell compartments. In Fig. 6, with the transversal application of the electrical field, a similar outcome was observed, with no difference in behavior between different types of cells. Figure 6d, f display the cell lysis products generated from medium and loosely packed chambers with HMC3 microglia cells, with an enhanced magnification of the cell debris in 6d. Various levels of success were achieved by switching the cell culture medium after stimulation to buffer compositions that supported chemical lysis, e.g., RIPA that contains ionic detergents such as SDS and sodium deoxycholate, or a dilute NH_4_HCO_3_ (10 mM) buffer system with 5–10 % organic solvent content. Electrical lysis in the latter was easier to perform, but could not be completed within a time-frame of a few seconds either.

As the purpose of cell lysis is to release the cell content for downstream analysis, one must consider not just the time delay between cell stimulation and lysis, but also the type of stresses that the cell is subjected to, during lysis, and the impact of these stresses on the biological processes that are under study and on the ability to detect the components of interest. While electrical cell lysis can be completed within µs-ms for single cells, the time-scale can increase to 3-10 min for large volumes of cells^27,28^. In non-uniform electrical fields, in the presence of high-currents and Joule heating, damage to the cell components is also expected, especially when the conditions persist on the minute-level time-scale. In addition, air bubbles generated at electrodes may enter the cell compartment, adding further challenges to achieving uniform cell processing conditions. Alternative designs that incorporate geometric variations of the chip channels, or chip-embedded electrodes placed in close proximity to each other, may help achieving high electrical field strengths at low voltages to enable effective cell lysis. Nevertheless, such designs come at the expense of possible constraints in cell handling operations or of more elaborate chip fabrication processes, respectively. Therefore, the removal of cells from the chip for lysis in an ultrasonicator device was considered to be a more advantageous option. After stimulation, the cells were quickly (<1 min) back-flushed from the cell compartment with ~100–200 µL hypotonic NH_4_HCO_3_ 10 mM/CH_3_OH (95:5 v/v) solution and collected in an Eppendorf vial. This process was accomplished from port (II) in the 1st design, and ports (III) or (IV) to (I), in the 2nd one. If osmotic shock interferes with the analysis, the cells should be removed from the chip in the stimulation medium. The cells were sonicated in an ice-cold sonicator bath, by applying ~10 × 10 s pulses, and then frozen immediately at −80 °C. Within 1–2 min, almost complete cell lysis was achievable (see Supplemental Fig. 1). The hypotonic buffer used for lysis contained phosphatase and protease inhibitors, to freeze the cell signaling machinery upon lysis and prevent the degradation of proteins by enzymes. The whole procedure was clean and very fast. The time-delay from cell stimulation to inducing lysis was ~1 min, and the whole sampling process, ~3–5 min. Performing quick cell lysis and protein extraction after stimulation is the most critical step for preserving the proteome profile of the biological response to the stimulation. Once the cells are lysed and the biological processes are disengaged, downstream handling of the lysis products poses no additional challenges.

## Discussion

### Biological challenges

To be able to utilize these microfluidic devices for biological research, the questions that are pursued, and the context in which the questions are asked, must be clearly defined. The response of cells to physical (e.g., heat, pressure, radiation) or chemical (e.g., various mitogens, growth factors, hormones) stimuli is very complex. It can be slow or very fast, unfolding on time-scales from minutes to days, and it can involve signaling processes that are transient or sustained. De novo protein synthesis may occur or not. Immediate-early responses (IER) involve critical cellular processes related, for example, to stress or immune response, or neuronal activity, and have essential roles in the regulation of cell cycle, glucose metabolism, growth, proliferation, differentiation, and oncogenic transformation^34,35^. Immediate-early genes (IEGs) are transcriptionally induced within 5–10 min of stimulation (e.g., *CFOS*, *CJUN, CMYC*, *EGR1, EGR2*), and do not involve de novo protein synthesis^34,35^. Their induction occurs via signal transduction pathways such as p38 MAPK, ERK, PI3K, and RhoA-actin^34^, that are activated within seconds or minutes of stimulation, and that involve rapid and/or transient protein phosphorylation processes. These genes, often transcription factors, are involved in controlling the next wave of gene and protein expression. Changes in protein expression, *per se*, would not be expected, however, earlier than ~30 min.

Whether monitoring changes in protein expression or in their PTMs, the experimental design must take, therefore, into account the following factors: the length and intensity of the stimulus that is inducing a change in the measured variable, the time-delay until the onset of the change, and the transient or sustained nature of the change. Hence, the axial infusion microreactor is expected to perform well only for experiments that do not involve fast stimulation experiments, but rather prolonged observation of cell behavior in a particular culture medium (e.g., experiments that require a comparative analysis of cell behavior when exposed to culture medium of different composition; this could be accomplished in multiplexed axial chips with independent chambers that do not communicate with each other). In addition, experiments that do not require a large number of cells and that can be accommodated in short microreactors, could also benefit from the simpler axial infusion design. The transversal cell stimulation microreactor, however, is expected to perform well for monitoring cellular responses that are induced as soon as ~1–2 min after stimulation (the assumption being that a 1 min pulse will be sufficient for ensuring uniform exposure of all cells to the stimulus), and that occur with a transient manifestation of no less than ~5 min (i.e., once induced, will not be affected or will not disappear within the time-frame that is necessary for cell removal from the chip and lysis). Signaling processes that involve fast phosphorylation events will be better explored with the transversal stimulation chip, as long as the dynamics of the phosphorylation/dephosphorylation process does not require second-level accuracy in cell sampling, and as long as the phosphorylated sites can be preserved during lysis. Biological responses that require de novo protein synthesis, progress over long time-scales, or have a sustained output (hours-days), will pose no challenge and can be investigated with either chip designs.

**Fig. 7:**
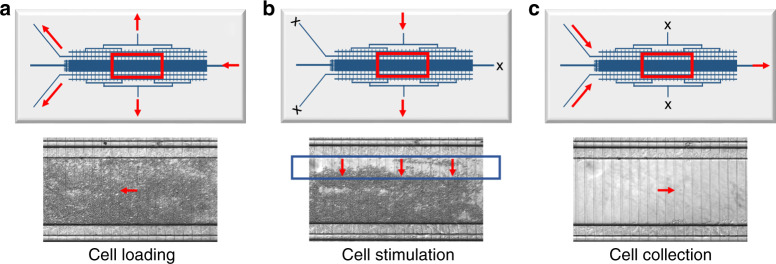
Cell loading, stimulation, and collection from a microfluidic chip with transversal delivery of the EGF solution to a ~500 nL cell chamber. **a** SKBR3 cell loading on the chip; **b** Stimulation with EGF solution (10 nM) infused at ~1 µL/min for ~ 3 min; **c** Empty cell compartment after quick SKBR3 flush-out with ~150–200 µL hypotonic buffer (NH_4_HCO_3_10 mM/CH_3_OH, 95:5 v/v) for off-chip lysis by sonication

**Fig. 8:**
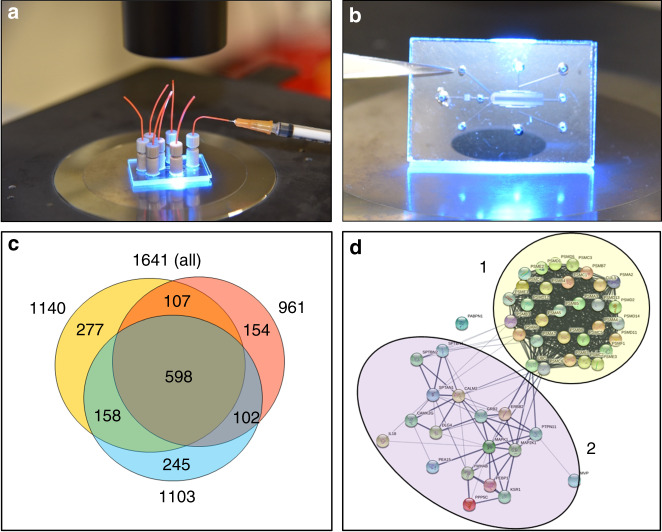
Microfluidic setup and proteomic results of a fast EGF-stimulation experiment of SKBR3 cells. **a**, **b** Pictures of a 1”×1.5” microfluidic chip with transversal delivery of the cell stimulation solution; **c** Venn diagram of protein overlaps between three replicate analyses of SKBR3 cells loaded on the chip, stimulated with EGF (10 nM) for 3 min, and collected for off-chip lysis by sonication; **d** STRING protein-protein interaction diagram of 50 proteins showing the interactions between the proteasome (1) and ERBB2-MAPK-ERK signaling (2)

To evaluate the applicability of the chips to explore challenging cell behaviors that unfold within a few minutes, SKBR3 breast cancer cells, which overexpress HER2 receptors, were loaded on a transversal stimulation chip (Figs. 7a, 8a, b), exposed to 3 min stimulation with EGF (10 nM) at 1 µL/min (Fig. 7b), flushed out quickly with hypotonic buffer (<1 min), and subjected to off-chip sonic cell lysis. Figure 7c provides the image of a clean cell compartment, demonstrating full cell recovery from the chip. Rinsing of the chips with 10% bleach solution enabled multiple usages of the same device. The lysis products generated from three biological replicates of stimulated SKBR3 cells were digested with trypsin and analyzed by nano-HPLC/MS/MS. The goal was to assess whether the cells that were stimulated and recovered from the chip enabled the identification of representative proteins that would allow the study of fast cellular responses to a perturbation. Without any enrichment procedure, ~960–1,100 protein groups (1,650 in total) were identifiable in each biological replicate, of which ~60% were consistently present in all three replicates (Fig. 8c and Supplemental Table 1). Functional analysis with the DAVID software package revealed that all major cellular compartments (nucleus, cytoplasm, membrane, mitochondria, endoplasmic reticulum, Golgi) were represented in the pool of identified proteins. In addition, rich protein clusters could be allocated to specialized compartments and complexes such as the proteasome, centrosome and the peroxisome. While extracellular proteins were not collected from the device, bioinformatics analysis of the data also revealed proteins known to be secreted via the exosomes. On the background of translation, cell–cell adhesion, protein transport and metabolic and redox processes, biological pathways related to proliferation, cell cycle, DNA repair, MAPK, ERBB2, p53, Wnt, NIK/NF-kappaB, and cell surface receptor signaling were just a few among the many processes that were represented in the list. A STRING protein-protein interaction (PPI) diagram (Fig. 8d) illustrates a network of 50 proteins that links the proteasome to MAPK, ERBB, and ERK signaling, the signal transduction pathways that are activated first, via phosphorylation, in response to extracellular stimuli. The cluster regulates a number of biological processes related to proliferation, apoptosis, and response to stress. The identification of such proteins is critical to studies aimed at understanding the intracellular signaling mechanisms that seek capturing phosphorylation events that transmit the signal from the cell surface to the nucleus and induce early-gene expression. On the flip side, with relevance to extracellular signaling, the presence of a large number of proteins (664) that have been previously identified in exosomes expands the scale of signaling processes that could be investigated with the device, which in the case of cancer cells can provide insights into the cellular mechanisms that support growth and metastasis^36^.

The cell stimulation chip provided reproducible results in terms of identifiable protein numbers from a defined number of cells. To further assess the applicability of the device to perform quantitative comparisons between different cell states, we assessed the quality of the data based on the ability to identify and quantify proteins that can be used for normalization, a necessary step that is performed prior to any quantitative comparison. The protein abundances were measured in terms of spectral counts. The normalization set included: (a) endogenous proteins commonly used as controls in biological research (i.e., actin, GAPDH, tubulin), (b) proteins that are part of an endogenous protein barcode that was developed in our laboratory for the normalization of cell cycle proteomics data (i.e., nucleolin, pyruvate kinase)^37^, and (c) standard bovine proteins (i.e., alpha/beta hemoglobin, alpha/beta casein, carbonic anhydrase, alpha-2-HS-glycoprotein) and stable-isotope labeled peptides (i.e., pyruvate kinase peptides), at known concentrations, that were spiked in the cell extracts. While the cell endogenous proteins are best used for normalization, the stable-isotope labeled peptides can also be used as a reference for performing targeted analysis and absolute quantitation. The stability of the spectral count data provided in Supplemental Table 2 demonstrates the quality of the data. With RSD values <30%, the results were consistent with previous experiments that indicated that for semi-quantitative approaches such as spectral counting, changes in protein expression levels above a ~2-fold threshold can be assessed^38^. For accurate quantitation, the implementation of stable-isotope labeling methods is straightforward^39^.

### Technological challenges

Based on estimates of cell size and cell loading capacity per chamber (i.e., 100,000 cells/250 nL according to theoretical calculations, and 50,000–100,000 cells/250 nL according to experimental observations), the total amount of proteins that can be extracted from one 250 nL microfluidic chamber is ~12–25 µg (or, 25–50 µg from a 500 nL chamber). MS analysis was performed from 1 µL injections of 0.5–1 µg/µL protein extract solutions, equivalent to at least ~2000 cells, enabling the identification of ~1000 proteins per LC-MS analysis, a typical outcome for a standard LC/Orbitrap MS set-up. Combined replicates, as often used to increase proteome coverage, led to 1641 protein IDs. To account for loosely packed cell chambers, the cell numbers would have to be further adjusted by multiplication with a correction factor of ~0.8, which would lead to slightly improved detection levels in terms of protein IDs per cell counts. As reported recently, however, LC-MS configurations that support LC operation under ultra-high pressures (900 bars, 75 cm nano-columns, 2 µm C18 particles, 240 min eluent gradients, 1–2 µg injections) can generate a substantial increase in protein IDs (~5,000 from three combined replicates)^40^. The use of high-efficiency separation PLOT columns^41^, of alternative data acquisition parameters, or of multiple ion fragmentation methods, could further extend the number of identified proteins and/or lower the number of necessary cells for analysis.

Our measurements of protein concentrations in cells led to values of 50–100 mg/mL PCV. This was calculated based on Bradford assays for proteins extracted from cells through sonication or nuclear/cytoplasmic fraction separation. Per 100 µL PCV, 5–10 mg soluble proteins were obtained. For SKBR3 cells of an average diameter of 15 µm/cell and a volume of ~1.8 × 10^−12^ L (or 1,800 µm^3^), this amounted to (120–240) × 10^−12^ g/cell (i.e., 120–240 pg/cell, considering 74% maximum theoretical packing density for cells with a spherical geometry; or, up to 240–480 pg/cell, if considering the lower end of the experimental counts of ~20 × 10^6^ cells per 100 µL PCV). The results are in full agreement with recent observations calculated from quantitative immunoblots and cell counts (~120–250 pg/cell in human cell lines)^42^. We must note, however, that the protein content of cells depends not just on the cell type but also on the cell proliferative stage, and that insoluble proteins may be lost during extraction. For an average protein length of 400 amino acids (aa) with an average mass of 110 Da/aa, the total number of protein molecules per cell can be deduced to be ~3.3 × 10^9^ (i.e., (240 × 10^−12^ g/cell)/(400 aa/protein × 110 Da/aa × 1.66 × 10^−24^ g/Da) = 3.3 × 10^9^ proteins/cell). To capitalize on the performance of advanced mass spectrometers, the minimum number of cells necessary for enabling the identification of a few thousands of proteins from only ~0.1–1 µg cell extract is estimated, therefore, to be ~400–4,000. Nevertheless, as the top 1,000 most abundant proteins in cells account for ~80–90% of the total protein content^43^, for detecting low copy number proteins in the presence of their more abundant counterparts, more complex multidimensional separations may be necessary with substantial upscaling in starting cell numbers. In addition, the presence of PTMs can profoundly diminish the ability to detect certain proteins. If the goal of the study is to study the actual PTM-modified proteins and peptides, the amounts needed for analysis could increase considerably, e.g., up to ~100-fold in the case of phosphoproteins^44^. Accurate quantitation will also benefit from the analysis of larger amounts. On the other hand, when only proteins of interest are monitored instead of the whole proteome, targeted detection such as parallel reaction monitoring can improve detection limits ~100-fold.

Altogether, our results indicate that if ~400–4,000 mammalian cells are sufficient for performing in-depth proteomic profiling, 5–50 nL cell culture chambers would be of sufficient size to enable replicate MS analyses from the same cell batch. This would facilitate the design of chips that enable multiplexed experiments, testing of reproducibility, or the use of various stimulation conditions. Handling very small protein amounts may raise however challenges related to sample losses through adsorption on the instrumentation walls. In the protocol that we have developed, the cells are removed from the chip prior to lysing, therefore protein losses from the cells do not represent a concern. Losses through adsorption may affect, however, some components of the stimulation medium that are present in very low, sub-nM concentrations. While proteins adsorb less on glass than on hydrophobic polymeric surfaces^45^, functionalizing the microfluidic channel surfaces may represent an effective solution to minimizing sample adsorption, in case of need, if the coating does not interfere with the behavior of cells. It is important to emphasize, however, that while surface-per-volume increases with a reduction in dimensions, the overall inner surface area of the chip that is exposed to cells or proteins is much less than the areas associated with sample handling during preparation or infusion through capillaries to the mass spectrometer. Therefore, when the analysis involves ng-level protein amounts, the optimization of the downstream processing steps, prior to MS detection, becomes critical.

## Conclusions

In this work, we developed and demonstrated microfluidic devices for enabling studies of cell behavior by using mass spectrometry detection. To the best of our knowledge, this is the first report that investigates the merger of the two technologies for advancing near real-time proteomic profiling of cellular responses to a stimulus. We evaluated the performance of the microfluidic chips in terms of ability to (i) accommodate a sufficient number of cells for enabling comprehensive mass spectrometry analysis, (ii) perform uniform cell stimulation, and (iii) facilitate the fast sampling of the cellular content for preserving the fidelity of the biological response to a perturbation. The chips enabled axial or transversal delivery of the cell stimulation agent. Visualization of fluid manipulations on the chip with fluorescent dyes confirmed the simulation results of the stimulation process. Devices with axial delivery of the stimulation agent will benefit studies of biological processes that: (a) require prolonged cell stimulation and are not responsive to the delay induced by the time needed to generate a homogeneous solution in the cell chamber; (b) are characterized by a biological response that is not affected by a small concentration gradient of the stimulant within the chamber; or, (c) do require an actual concentration gradient of the stimulant to observe the variability in biological response. Conversely, devices with transversal delivery of the cell stimulation agent will benefit studies aimed at: (a) exploring biological processes that are characterized by a quick response to the cell stimulation; (b) necessitate rapid and uniform delivery of the stimulant to the entire cell compartment; and (c) require rapid cell lysis to freeze the cellular response machinery to the applied stimulus. Quick removal of cells from the device for performing sonic lysis proved to be an effective and reproducible approach for minimizing the time-delay (< 1 min) between the stimulation and sampling events. Proteomic profiling from ~2,000 cells demonstrated that the newly devised chips enable a systems-level assessment of the cell behavior, to capture biological events that are critically responsive to a perturbation. The thousands of MS readouts provide rich information reflective of the system’s status and of the dominant mechanisms that are at work within the cell. The use of mass spectrometers with improved sensitivity and detection limits is expected to lead to similar or even better results from the equivalent of only a few hundred cells, a number that is expected to further drop with continuous advances in MS instrumentation, scanning functions, and sample separation strategies.

## Materials and methods

### Materials

SKBR3 human breast cancer cells, HBEC-5i human brain endothelial cells, HMC3 microglia cells, phosphate buffered saline (PBS), and trypsin/EDTA were purchased from the American Tissue Culture Collection (ATCC, Manassas, VA). Cell culture media including McCoy’s 5 A, EMEM, and DMEM/F12 (1:1) were obtained from Life Technologies (Carlsbad, CA), fetal bovine serum (FBS) from Gemini Bio-Products (West Sacramento, CA), Normocin from InvivoGen (San Diego, CA), and human epidermal growth factor (hEGF) from PeproTech (Rocky Hill, NJ). Sequencing grade modified trypsin was purchased from Promega Corporation (Madison, WI). Endothelial cell growth supplement (ECGS), RIPA buffer, urea, dithiothreitol (DTT), acetone, glacial acetic acid, trifluoroacetic acid (TFA), ammonium bicarbonate, phosphatase and protease inhibitor cocktails, ammonium hydroxide, hydrogen peroxide (35%) and protein standards were purchased from Sigma-Aldrich (St. Louis, MO). Carboxylate modified latex (CML) beads, 10–11 µm diameter, were from Invitrogen/Thermo Fisher. SPEC-PT-C18 and SPEC-PT-SCX solid phase extraction pipette tips, and 5 µm/SB-C18 Zorbax particles, were purchased from Agilent Technologies (Santa Clara, CA). Fused silica capillary columns were from Polymicro Technologies (Phoenix, AZ). Heavy forms of synthetic peptides (^13^C/^15^N stable-isotope label of +7 Da incorporated at Leu) were synthesized by New England Peptide (Gardner, MA). Ammonium hydroxide (28–30%) was purchased from Spectrum Chemical (New Brunswick, NJ). Buffered oxide etch (BOE) and chromium etchant were from Transene Company (Danvers, MA), and MF-319 developer from Rohm and Haas (Philadelphia, PA). HPLC-grade acetonitrile and methanol were from Fisher Scientific (Fair Lawn, NJ), and DI water was prepared with a MilliQ Ultrapure water system (Millipore, Bedford, MA).

### Cell culture and processing

The human cell lines were cultured in the growth medium recommended by the manufacturer, at 37 °C in an incubator, with 5% CO_2_ atmosphere: SKBR3 breast cancer cells in McCoy’s 5 A^46,47^, HMC3 microglia in EMEM, and HBEC5i endothelial cells in DMEM/F12. All culture media were supplemented with 10% FBS, Normocin (0.1 mg/mL), and the endothelial cells with ECGS (40 µg/mL). The cells were harvested by trypsinization, washed with cold PBS, resuspended either in PBS or culture medium (1:1 v/v slurry of packed cell volume (PCV):PBS), aspirated into a 1 mL syringe (blunt tip, gauge 26), loaded into the chip by gentle dispensing from the syringe barrel, and equilibrated with culture medium. SKBR3 cells were stimulated with EGF (~10 nM in culture medium) for 3 min. Cell lysis was performed on the chip in electrical fields, in the culture medium or in lysis buffers of various composition [hypotonic NH_4_HCO_3_ (10 mM) with CH_3_OH (5%) or CH_3_CN (10%), or, RIPA buffer at 10X dilution]. Alternatively, the cells were quickly flushed out in ~100–200 µL hypotonic NH_4_HCO_3_ buffer and lysed by sonication (10 bursts of ~10 sec in ice-cold water, with intermittent pausing to avoid sample heating). All lysis buffers contained protease (1%) and phosphatase (2%) inhibitors, and DTT (1 mM). The cell lysates were frozen at −80 °C prior to further processing.

### Tryptic digestion

The frozen cell extracts were thawed, and ammonium bicarbonate (500 mM, 12 µL), urea (0.048 g) and DTT (100 mM, 5 µL) were added to denature/reduce the protein extracts at 58 °C for 1 h (volumes shown are per 100 µL cell extract). To prevent the generation of side-products, protein alkylation after reduction was not performed. The extracts were diluted 10-fold with NH_4_HCO_3_ (50 mM), digested with trypsin at 37 °C overnight (10 µg trypsin per cell extract collected from one microfluidic chamber), and quenched with glacial CH_3_COOH (digestion solution/CH_3_COOH, 100:1, v/v). Stable-isotope-labeled synthetic peptides (10 µL, 5–10 µM) were added to the extract of each cell chamber. The samples were centrifuged at 16,000×*g* (5 min), cleaned-up by using SPEC-PT-C18 and SPEC-PT-SCX solid phase extraction pipette tips, brought close to dryness in a vacuum centrifuge, and resuspended in 50 µL of H_2_O/CH_3_CN/TFA 98:2:0.01 v/v to a final concentration ~0.5–1 μg/μL.

### LC-MS/MS analysis and data processing

The samples were analyzed with an Orbitrap Fusion Lumos mass spectrometer (Thermo Fisher, San Jose, CA), ESI voltage +2.4 kV, by using a Waters nanoAcquity^TM^ UPLC system (Waters, Milford, MA) and an Acquity UPLC Peptide BEH C18 separation column (100 μm i.d. × 10 cm length, 1.7 µm particles) operated at 500 nL/min. Eluents A and B were water and acetonitrile, respectively, each with 0.1% HCOOH. The separation gradient was 110 min long, with B increasing from 3 to 90%. MS acquisition was performed over a range of 400–1,500 m/z, Orbitrap resolution 120,000 at 200 m/z, automatic gain control (AGC) target 400,000, and max. injection time 50 ms; MS2 isolation was in the quadrupole with isolation window 1.6; collision induced dissociation (CID) occurred in the linear trap, at collision energy 35%, activation Q 0.25, precursor intensity threshold 5,000, charge exclusion of z = 1 ions; dynamic exclusion was enabled for 60 s, with low/high mass tolerance 10 ppm; acquisition was top-speed data-dependent mode with most intense priority. The MS raw files were analyzed with the Thermo Proteome Discoverer 1.4 package and the Sequest HT search engine. For protein identifications, a *Homo sapiens* database with 20,197 reviewed/non-redundant protein sequences was downloaded from UniProt (January 2015). The parameters for database search included: 500–5,000 mass range, precursor ion tolerance 10 ppm, fragment ion tolerance 0.6 Da, *b/y/a* ion fragments only, fully tryptic fragments, 2 missed cleavages, min/max peptide length of 6/144 amino acids, and no PTMs allowed. Database search FDRs were calculated with the Target Decoy PSM Validator node based on the Xcorr vs. charge state values, with cut-off settings of 1% (stringent) and 3% (relaxed). Three biological cell replicates were processed on the chip. Protein functional categories were assigned with the DAVID (Database for Annotation, Visualization, and Integrated Discovery)^48^ and STRING (Search Tool for the Retrieval of Interacting Genes/Proteins) bioinformatics platforms^49^.

### Chip fabrication

Chip layouts were designed with AutoCAD (Autodesk, San Rafael, CA) and flow simulation was performed with the COMSOL Multiphysics Modeling Software package (COMSOL, Inc., Burlington, MA). The photomasks were prepared by HTA Photomask (San Jose, CA). Glass substrates of 1.6 mm thick white crown glass, coated with chrome and photoresist, were purchased from Nanofilm (Shelton, CA). The UV light source was from OAI (San Jose, CA). The glass substrates were covered with the photomask, exposed to UV, developed in MF-319 solution, and subjected to chrome removal and glass etching in BOE^50^. The drawing dimensions of the cell reactors were 500 µm or 1000 µm in width (W) and 10 mm in length (L), of the cell inlet/outlet channels of W = 100 µm, and of the lateral waste collection channels of W = 20 µm. Etch depth (D) was ~50–60 μm (RSD~2–3%) for the cell chambers and D~1.5–4 µm (RSD < 10%) for the filter elements. After etching, the cell chamber widths became ~600 µm or ~1,100 µm. Channel dimensions were measured with a Dektak profilometer (Veeco, Plainview, NY). Channel access holes (~1 mm i.d.) were drilled with a rotary tool (Dremel, Racine, WI). After the removal of the photoresist and chrome, the glass substrates were cleaned, hydrolyzed in a boiling mixture of distilled DI water/NH_4_OH (28 %)/H_2_O_2_ (35 %) (2:1:1, v/v), and bonded by gradual heating from room temperature to 550 °C. Connecting ports to the chip were prepared from PEEK unions (Valco Instruments, Houston, TX) secured to the chip with a fast-drying, two component Epoxy adhesive (Loctite E-30CL, Henkel Corp., Westlake, OH). Connecting tubing to the inlet/outlet ports were from PEEK or FEP (VICI, Houston, TX), (0.015–0.02)” i.d. x 1/32” o.d. An in-house built power supply controlled by LabVIEW (National Instruments, Austin, TX) was used to apply voltage to the chip reservoirs via Pt electrodes. Fluid flows and cell behavior on the chip were observed with a Nikon epi-fluorescence microscope (Melville, NY). Prior to use, the chips were rinsed with 70 % ethanol and exposed to UV radiation in the biosafety hood.

## Supplementary information

Simulation of transversal infusion

Simulation of axial infusion

Supplemental figure 1

Supplemental table 1

Supplemental table 2
