# Supplementary figures and images for: Microfluidic reactors for advancing the MS analysis of fast biological responses
Source: Microsyst Nanoeng. 2019 Feb 11;5:7. doi: 10.1038/s41378-019-0048-3 (PMC6369226; doi:10.1038/s41378-019-0048-3)

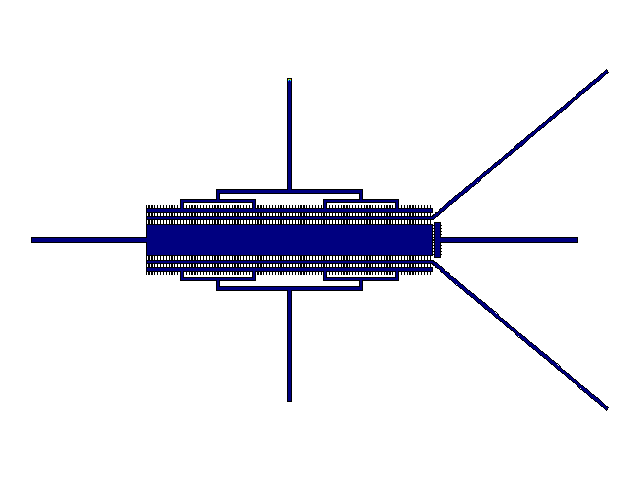

Supplement: Supplementary file 1 — Simulation of transversal infusion [file 41378_2019_48_MOESM1_ESM.gif]

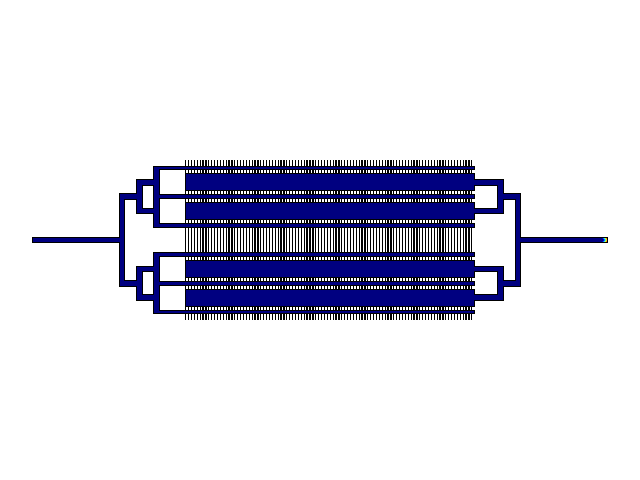

Supplement: Supplementary file 2 — Simulation of axial infusion [file 41378_2019_48_MOESM2_ESM.gif]
