# Supplementary material for: Microfluidic reactors for advancing the MS analysis of fast biological responses
Source: Microsyst Nanoeng. 2019 Feb 11;5:7. doi: 10.1038/s41378-019-0048-3 (PMC6369226; doi:10.1038/s41378-019-0048-3)

SKBR3 cells in 10 mM NH_4HCO_3 solution

(shortly after removing the cells from PBS and introducing them in NH_4HCO_3 solution)

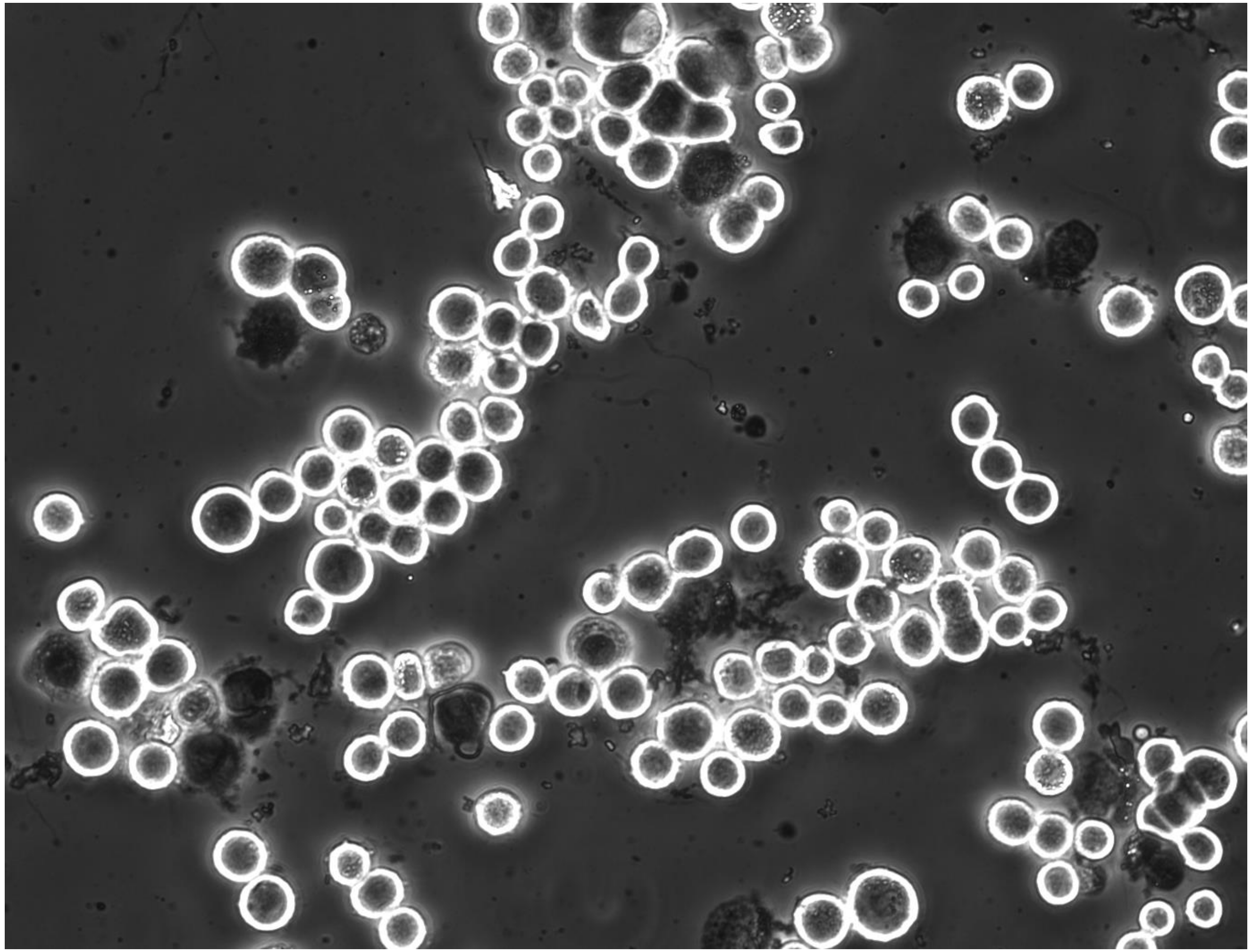

SKBR3 cell sonic lysis in 10 mM NH_4HCO_3 solution (1-2 min)

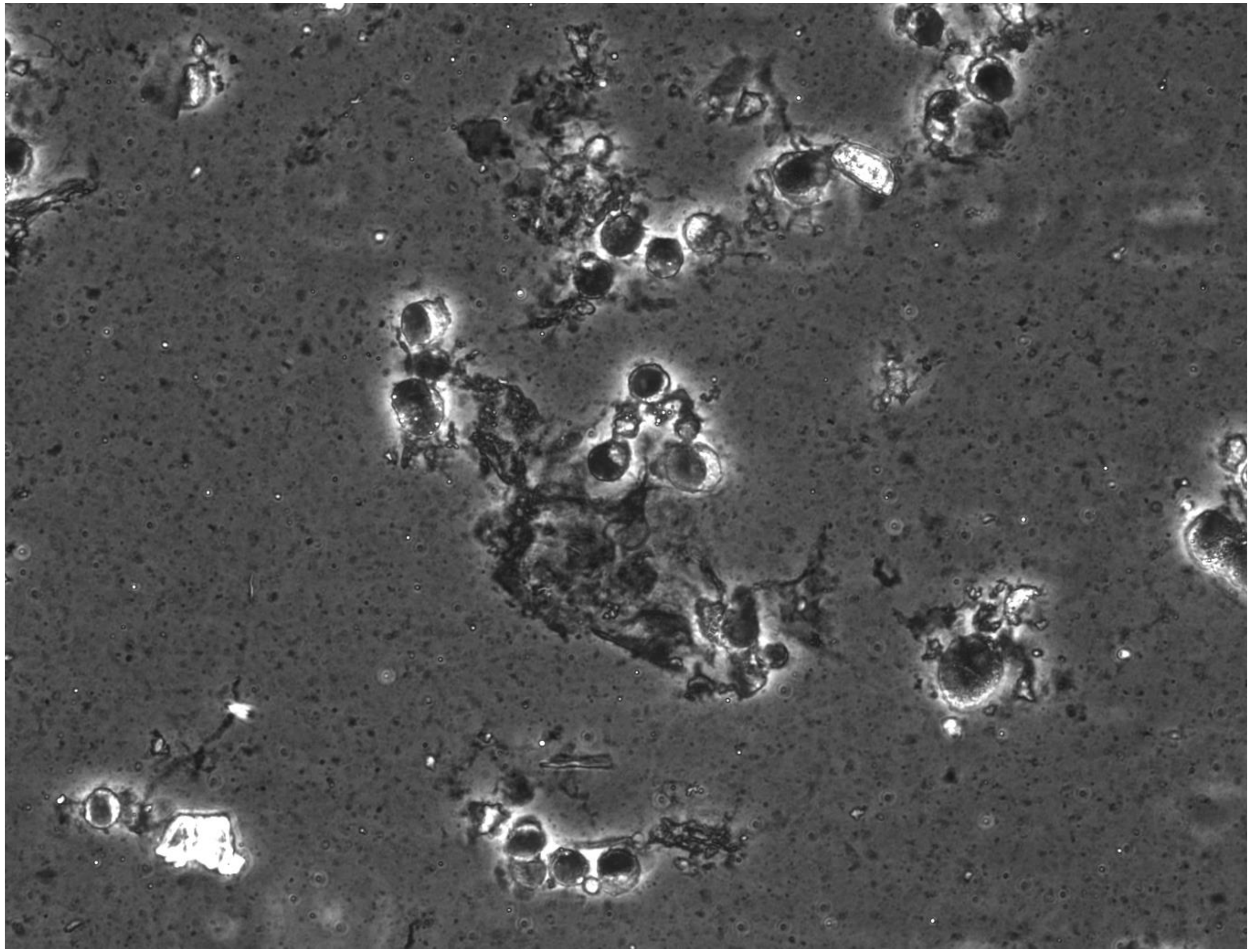

SKBR3 cell sonic lysis in 10 mM NH_4HCO_3 solution (2-3 min)

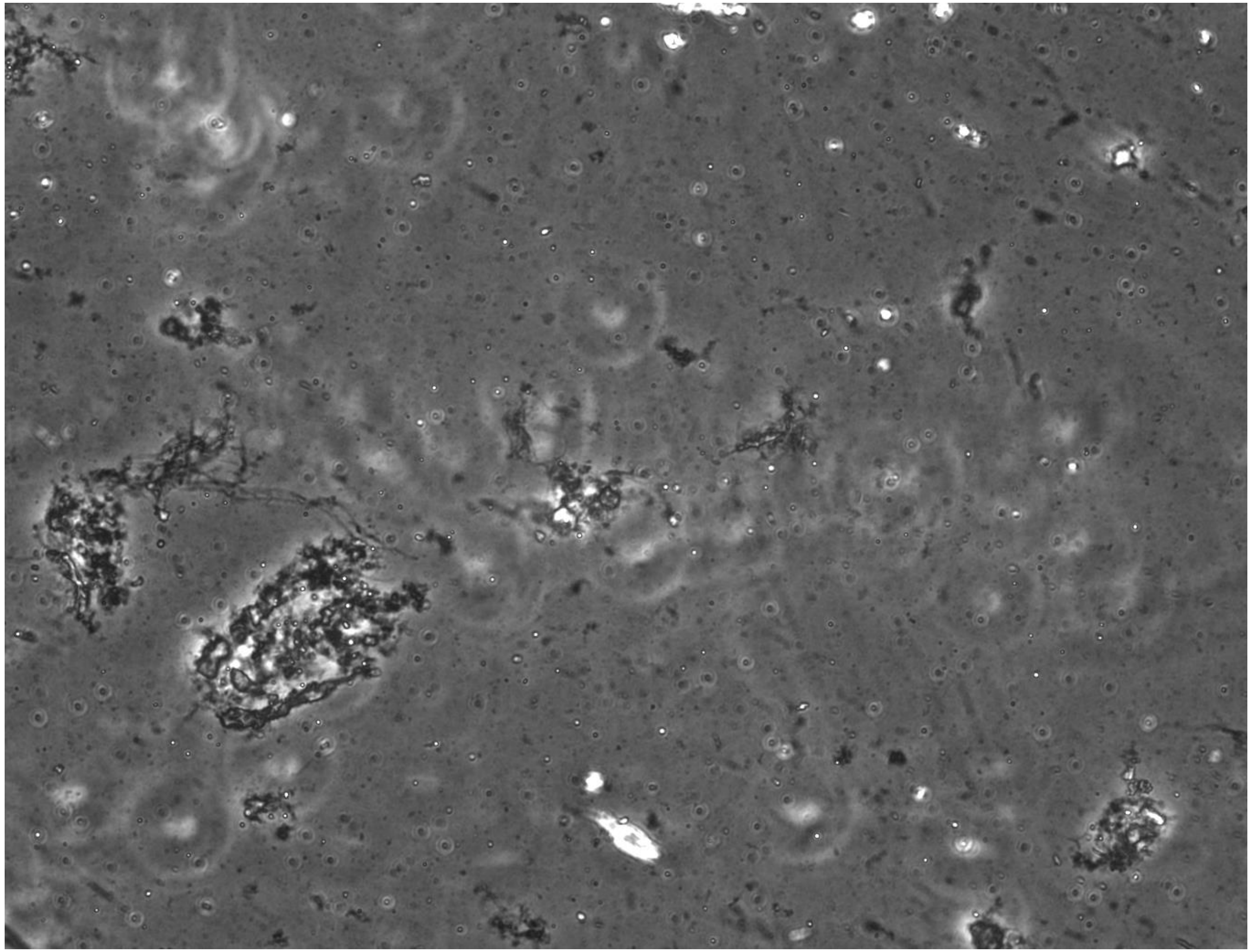

Supplement: Supplementary file 3 — Supplemental figure 1 [file 41378_2019_48_MOESM3_ESM.pdf]
