# Supplementary material for: Microfluidic reactors for advancing the MS analysis of fast biological responses
Source: Microsyst Nanoeng. 2019 Feb 11;5:7. doi: 10.1038/s41378-019-0048-3 (PMC6369226; doi:10.1038/s41378-019-0048-3)
Supplement: Supplementary file 4 — Supplemental table 1 [file 41378_2019_48_MOESM4_ESM.pdf]

| Accession | Description | Score A | Coverage A | # Peptides A | # PSM A | Score B | Coverage B | # Peptides B | # PSM B | Score C | Coverage C | # Peptides C | # PSM C |
|---|---|---|---|---|---|---|---|---|---|---|---|---|---|
| P05387 | 60S acidic ribosomal prot | 35.67 | 69.57 | 4 | 7 | 30.65 | 59.13 | 3 | 6 | 15.23 | 16.52 | 1 | 3 |
| P01966 | Hemoglobin subunit alpha | 35.18 | 46.10 | 4 | 7 | 37.72 | 54.61 | 4 | 7 | 46.19 | 57.45 | 4 | 9 |
| P04406 | Glyceraldehyde-3-phosph | 297.69 | 56.12 | 10 | 55 | 191.59 | 31.64 | 5 | 36 | 270.05 | 40.60 | 8 | 47 |
| P00921 | Carbonic anhydrase 2 - B | 82.93 | 41.70 | 6 | 16 | 71.91 | 50.19 | 7 | 14 | 60.83 | 25.10 | 4 | 11 |
| P05787 | Keratin, type II cytoskele | 92.33 | 45.76 | 13 | 18 | 93.42 | 39.13 | 11 | 17 | 145.80 | 39.96 | 11 | 27 |
| P02070 | Hemoglobin subunit beta | 116.45 | 30.34 | 3 | 23 | 117.81 | 30.34 | 3 | 24 | 128.32 | 30.34 | 3 | 25 |
| P02662 | Alpha-S1-casein - Bos tau | 73.62 | 45.79 | 5 | 14 | 46.37 | 35.51 | 3 | 9 | 32.94 | 22.43 | 3 | 7 |
| Q13268 | Dehydrogenase/reductase | 52.47 | 32.86 | 4 | 11 | 65.49 | 33.21 | 4 | 14 | 99.83 | 33.21 | 4 | 21 |
| P06576 | ATP synthase subunit bet | 64.31 | 37.05 | 11 | 13 | 61.41 | 34.03 | 10 | 13 | 39.64 | 17.39 | 6 | 8 |
| O43852 | Calumenin OS=Homo sap | 29.77 | 35.24 | 5 | 6 | 25.09 | 28.89 | 4 | 5 | 20.62 | 15.56 | 2 | 4 |
| P06733 | Alpha-enolase OS=Homo | 219.44 | 38.02 | 9 | 45 | 173.97 | 37.10 | 9 | 36 | 316.58 | 22.12 | 6 | 65 |
| P30086 | Phosphatidylethanolamine | 9.50 | 24.60 | 2 | 2 | 18.00 | 14.97 | 3 | 4 | 16.25 | 35.29 | 3 | 3 |
| P60842 | Eukaryotic initiation facto | 30.75 | 30.54 | 6 | 6 | 14.71 | 14.78 | 3 | 3 | 42.22 | 33.00 | 8 | 9 |
| Q15847 | Adipogenesis regulatory factor OS=Hc |  | 0.00 |  |  |  | 0.00 |  |  | 6.02 | 40.79 | 1 | 1 |
| Q8IUE6 | Histone H2A type 2-B OS | 11.10 | 40.00 | 2 | 2 | 5.49 | 17.69 | 1 | 1 | 5.62 | 17.69 | 1 | 1 |
| P21796 | Voltage-dependent anion | 24.50 | 33.22 | 5 | 5 | 24.49 | 33.22 | 5 | 5 | 30.06 | 27.92 | 4 | 6 |
| Q9UK76 | Hematological and neurol | 3.97 | 11.69 | 1 | 1 | 4.24 | 11.69 | 1 | 1 | 5.32 | 27.27 | 1 | 1 |
| P08727 | Keratin, type I cytoskeleta | 305.42 | 26.75 | 7 | 58 | 205.65 | 31.00 | 8 | 41 | 349.78 | 21.25 | 5 | 65 |
| P05783 | Keratin, type I cytoskeleta | 94.77 | 37.44 | 10 | 17 | 78.74 | 37.67 | 10 | 14 | 90.60 | 34.65 | 7 | 16 |
| P10809 | 60 kDa heat shock protei | 89.71 | 37.35 | 12 | 17 | 73.92 | 29.32 | 9 | 13 | 99.89 | 32.11 | 11 | 18 |
| Q96KK5 | Histone H2A type 1-H OS | 14.07 | 37.50 | 2 | 3 | 14.17 | 14.84 | 1 | 3 | 9.72 | 14.84 | 1 | 2 |
| P62158 | Calmodulin OS=Homo sa | 24.46 | 36.91 | 4 | 5 | 13.07 | 34.23 | 3 | 3 | 14.11 | 36.91 | 3 | 3 |
| P22626 | Heterogeneous nuclear ri | 29.75 | 19.83 | 4 | 6 | 39.57 | 36.26 | 6 | 8 | 37.31 | 24.08 | 4 | 8 |
| P55327 | Tumor protein D52 OS=H | 38.94 | 36.16 | 6 | 8 | 40.73 | 29.02 | 4 | 8 | 18.52 | 29.02 | 3 | 4 |
| P11142 | Heat shock cognate 71 kI | 36.97 | 23.53 | 8 | 8 | 34.02 | 13.62 | 5 | 7 | 79.47 | 26.78 | 8 | 15 |
| P23528 | Cofilin-1 OS=Homo sapie | 42.02 | 35.54 | 4 | 8 | 23.71 | 35.54 | 4 | 5 | 42.66 | 35.54 | 4 | 8 |
| P12763 | Alpha-2-HS-glycoprotein - | 599.23 | 30.64 | 5 | 102 | 653.17 | 30.64 | 5 | 110 | 659.70 | 35.38 | 6 | 111 |
| O43707 | Alpha-actinin-4 OS=Homo | 69.26 | 23.82 | 13 | 14 | 55.13 | 20.31 | 10 | 11 | 73.04 | 25.36 | 13 | 15 |
| O75223 | Gamma-glutamylcyclotran | 14.32 | 21.28 | 2 | 3 | 9.61 | 22.87 | 2 | 2 | 8.93 | 21.28 | 2 | 2 |
| Q8WXC6 | Myeloma-overexpressed gene 2 protein OS=Homo sapiens GN=MYEOV2 PE |  |  |  |  | 4.14 | 35.09 | 1 | 1 | 4.19 | 35.09 | 1 | 1 |
| P04075 | Fructose-bisphosphate ald | 166.31 | 34.89 | 7 | 33 | 126.24 | 34.89 | 7 | 26 | 141.40 | 24.73 | 4 | 27 |
| P07437 | Tubulin beta chain OS=H | 44.31 | 29.05 | 7 | 9 | 36.04 | 25.90 | 6 | 7 | 42.08 | 22.97 | 5 | 8 |
| P04264 | Keratin, type II cytoskele | 26.34 | 12.58 | 6 | 6 | 56.09 | 24.07 | 10 | 12 | 42.36 | 25.00 | 8 | 9 |
| P68371 | Tubulin beta-4B chain OS | 24.65 | 19.10 | 5 | 5 | 36.34 | 28.54 | 6 | 7 | 36.19 | 16.85 | 4 | 7 |
| P08758 | Annexin A5 OS=Homo sa | 27.11 | 26.25 | 5 | 6 | 24.13 | 21.56 | 4 | 5 | 20.61 | 17.50 | 3 | 4 |
| P0CW22 | 40S ribosomal protein S1 | 9.87 | 32.59 | 2 | 2 | 5.04 | 16.30 | 1 | 1 | 19.70 | 32.59 | 2 | 4 |
| P16152 | Carbonyl reductase [NAD | 24.14 | 21.30 | 4 | 5 | 19.76 | 21.30 | 4 | 4 | 21.19 | 28.16 | 4 | 4 |
| P14618 | Pyruvate kinase PKM OS= | 81.12 | 29.38 | 10 | 17 | 43.95 | 13.94 | 4 | 9 | 66.03 | 26.93 | 10 | 14 |

| | | | | | | | | | | | | | |
|---|---|---|---|---|---|---|---|---|---|---|---|---|---|
| P68363 | Tubulin alpha-1B chain O | 49.87 | 23.95 | 6 | 10 | 44.74 | 23.95 | 6 | 9 | 56.97 | 32.15 | 8 | 12 |
| Q13011 | Delta(3,5)-Delta(2,4)-dier | 10.31 | 12.80 | 2 | 2 | 13.75 | 19.51 | 3 | 3 | 20.84 | 25.30 | 4 | 4 |
| P62894 | Cytochrome c - Bos tauru | 19.63 | 30.77 | 2 | 4 | 5.30 | 16.35 | 1 | 1 | 8.53 | 30.77 | 2 | 2 |
| P31937 | 3-hydroxyisobutyrate deh | 22.93 | 26.19 | 5 | 5 | 14.21 | 14.88 | 3 | 3 | 13.85 | 15.77 | 3 | 3 |
| P07737 | Profilin-1 OS=Homo sapie | 17.54 | 20.00 | 2 | 4 | 12.80 | 31.43 | 3 | 3 | 23.01 | 20.00 | 2 | 5 |
| P04792 | Heat shock protein beta-1 | 18.81 | 31.22 | 4 | 4 | 19.28 | 31.22 | 4 | 4 | 14.86 | 31.22 | 3 | 3 |
| P06703 | Protein S100-A6 OS=Hon | 9.98 | 31.11 | 2 | 2 | | 0.00 | | | | 0.00 | | |
| P68036 | Ubiquitin-conjugating enz | 6.27 | 16.23 | 1 | 1 | 10.65 | 16.23 | 1 | 2 | 10.10 | 30.52 | 2 | 2 |
| P07237 | Protein disulfide-isomeras | 39.29 | 28.15 | 6 | 8 | 32.17 | 13.78 | 4 | 7 | 43.12 | 26.97 | 6 | 8 |
| P78371 | T-complex protein 1 subu | 49.58 | 26.73 | 10 | 10 | 56.03 | 25.98 | 9 | 11 | 44.77 | 23.93 | 8 | 9 |
| P31946 | 14-3-3 protein beta/alpha | 26.13 | 21.14 | 3 | 5 | 29.61 | 21.14 | 3 | 6 | 9.87 | 16.67 | 2 | 2 |
| P20674 | Cytochrome c oxidase sul | 9.60 | 30.00 | 2 | 2 | | 0.00 | | | 4.24 | 10.00 | 1 | 1 |
| P29401 | Transketolase OS=Homo | 38.07 | 23.92 | 7 | 8 | 43.80 | 18.14 | 5 | 8 | 42.63 | 22.15 | 5 | 7 |
| O95994 | Anterior gradient protein | 19.43 | 29.71 | 3 | 4 | 8.98 | 20.00 | 2 | 2 | 5.12 | 9.71 | 1 | 1 |
| P51858 | Hepatoma-derived growtl | 15.91 | 29.58 | 3 | 3 | 16.57 | 18.33 | 3 | 3 | 20.77 | 18.33 | 4 | 4 |
| P26599 | Polypyrimidine tract-bindi | 19.76 | 17.14 | 4 | 4 | 15.48 | 13.18 | 3 | 3 | 11.55 | 11.68 | 2 | 2 |
| P37802 | Transgelin-2 OS=Homo s | 8.71 | 16.08 | 2 | 2 | 13.83 | 22.11 | 3 | 3 | 13.77 | 22.11 | 3 | 3 |
| P09211 | Glutathione S-transferase | 16.38 | 20.00 | 2 | 3 | 21.46 | 29.05 | 3 | 4 | 6.53 | 9.05 | 1 | 1 |
| P35527 | Keratin, type I cytoskelet | 28.79 | 17.82 | 4 | 5 | 50.45 | 26.48 | 6 | 9 | 31.79 | 13.96 | 4 | 6 |
| P67809 | Nuclease-sensitive eleme | 18.15 | 9.88 | 2 | 4 | 14.97 | 18.21 | 3 | 3 | 28.39 | 29.01 | 5 | 6 |
| O14548 | Cytochrome c oxidase subunit 7A-rela | | 0.00 | | | 5.43 | 28.95 | 1 | 1 | | 0.00 | | |
| P62841 | 40S ribosomal protein S1 | 11.62 | 28.28 | 2 | 2 | 10.23 | 13.10 | 1 | 2 | 5.02 | 13.10 | 1 | 1 |
| P08865 | 40S ribosomal protein SA | 14.22 | 17.97 | 3 | 3 | 18.80 | 15.93 | 3 | 4 | 16.84 | 13.56 | 2 | 3 |
| Q16698 | 2,4-dienoyl-CoA reductas | 18.86 | 23.58 | 4 | 4 | 12.69 | 16.12 | 3 | 3 | 19.68 | 15.82 | 4 | 4 |
| P02769 | Serum albumin - Bos taur | 535.66 | 24.88 | 12 | 122 | 525.64 | 20.76 | 10 | 118 | 424.37 | 18.29 | 8 | 94 |
| P61204 | ADP-ribosylation factor 3 | 24.15 | 27.62 | 3 | 5 | 8.14 | 17.68 | 2 | 2 | 4.56 | 7.73 | 1 | 1 |
| P47755 | F-actin-capping protein su | 10.83 | 14.69 | 2 | 2 | 14.07 | 18.53 | 3 | 3 | 4.19 | 5.59 | 1 | 1 |
| Q02790 | Peptidyl-prolyl cis-trans is | 37.69 | 20.26 | 5 | 8 | 27.87 | 24.18 | 6 | 6 | 20.78 | 17.43 | 4 | 4 |
| P61978 | Heterogeneous nuclear ri | 39.99 | 18.14 | 5 | 8 | 29.99 | 15.33 | 4 | 6 | 46.12 | 24.19 | 6 | 9 |
| P60709 | Actin, cytoplasmic 1 OS= | 114.87 | 27.20 | 6 | 23 | 65.17 | 18.93 | 4 | 13 | 149.20 | 27.20 | 6 | 29 |
| Q9UL46 | Proteasome activator con | 30.46 | 27.20 | 4 | 6 | 20.47 | 27.20 | 4 | 4 | 25.95 | 21.34 | 3 | 5 |
| Q15181 | Inorganic pyrophosphatas | 24.74 | 20.42 | 4 | 5 | 18.19 | 13.15 | 3 | 4 | 30.59 | 21.45 | 4 | 6 |
| P61106 | Ras-related protein Rab-1 | 13.01 | 26.98 | 3 | 3 | | 0.00 | | | | 0.00 | | |
| P38646 | Stress-70 protein, mitoch | 57.72 | 20.32 | 10 | 13 | 45.95 | 16.35 | 8 | 10 | 47.10 | 16.35 | 7 | 10 |
| Q9Y316 | Protein MEMO1 OS=Hom | 13.34 | 16.50 | 3 | 3 | 4.84 | 6.40 | 1 | 1 | 13.81 | 22.56 | 3 | 3 |
| Q13228 | Selenium-binding protein | 24.54 | 19.07 | 5 | 6 | 21.57 | 9.11 | 3 | 5 | 18.03 | 12.29 | 4 | 4 |
| Q16881 | Thioredoxin reductase 1, | 35.52 | 15.72 | 6 | 7 | 17.67 | 11.86 | 3 | 3 | 25.82 | 13.71 | 5 | 5 |
| P62081 | 40S ribosomal protein S7 | 11.10 | 26.80 | 2 | 2 | 4.61 | 11.34 | 1 | 1 | 5.90 | 11.34 | 1 | 1 |
| P27348 | 14-3-3 protein theta OS= | 30.58 | 26.53 | 6 | 6 | 34.72 | 21.63 | 5 | 7 | 9.91 | 13.47 | 2 | 2 |

| | | | | | | | | | | | | | |
|---|---|---|---|---|---|---|---|---|---|---|---|---|---|
| P20339 | Ras-related protein Rab-5 | 8.80 | 15.35 | 2 | 2 | | 0.00 | | | 9.20 | 17.67 | 2 | 2 |
| P50151 | Guanine nucleotide-binding protein G( | | 0.00 | | | 4.40 | 26.47 | 1 | 1 | | 0.00 | | |
| P51148 | Ras-related protein Rab-5 | 16.53 | 26.39 | 3 | 3 | 21.24 | 26.39 | 3 | 4 | 15.25 | 26.39 | 3 | 3 |
| O60361 | Putative nucleoside diphc | 25.66 | 26.28 | 2 | 5 | 15.95 | 13.87 | 1 | 3 | 10.99 | 13.87 | 1 | 2 |
| O00264 | Membrane-associated prc | 9.38 | 26.15 | 2 | 2 | 4.63 | 10.26 | 1 | 1 | 5.22 | 10.26 | 1 | 1 |
| Q9NS69 | Mitochondrial import rece | 3.86 | 8.45 | 1 | 1 | 12.74 | 17.61 | 1 | 2 | 5.47 | 17.61 | 1 | 1 |
| Q9H0U4 | Ras-related protein Rab-1 | 13.47 | 17.41 | 2 | 3 | 23.86 | 25.87 | 3 | 5 | 17.49 | 25.87 | 3 | 4 |
| P21266 | Glutathione S-transferase | 13.68 | 19.56 | 3 | 3 | 4.47 | 6.67 | 1 | 1 | 8.71 | 12.00 | 2 | 2 |
| P02655 | Apolipoprotein C-II OS=Homo sapiens | | 0.00 | | | | | | | 5.24 | 25.74 | 1 | 1 |
| P50395 | Rab GDP dissociation inhi | 41.49 | 21.12 | 6 | 8 | 35.96 | 21.12 | 6 | 7 | 30.03 | 13.26 | 5 | 6 |
| P09972 | Fructose-bisphosphate al | 51.95 | 19.51 | 5 | 10 | 21.12 | 10.71 | 2 | 4 | 34.17 | 17.03 | 4 | 7 |
| P61604 | 10 kDa heat shock protei | 9.19 | 25.49 | 2 | 2 | 9.39 | 13.73 | 1 | 2 | 4.76 | 13.73 | 1 | 1 |
| P00167 | Cytochrome b5 OS=Hom | 9.00 | 25.37 | 2 | 2 | 10.58 | 15.67 | 1 | 2 | 5.75 | 15.67 | 1 | 1 |
| P62820 | Ras-related protein Rab-1 | 22.67 | 25.37 | 3 | 5 | 23.50 | 25.37 | 3 | 5 | 22.17 | 25.37 | 3 | 5 |
| P63104 | 14-3-3 protein zeta/delta | 17.22 | 19.59 | 3 | 3 | 35.28 | 20.82 | 3 | 7 | 21.87 | 15.10 | 2 | 4 |
| P08133 | Annexin A6 OS=Homo sa | 26.94 | 13.52 | 6 | 6 | 38.12 | 18.28 | 7 | 8 | 33.95 | 17.09 | 6 | 7 |
| P14927 | Cytochrome b-c1 comple: | 3.83 | 13.51 | 1 | 1 | | 0.00 | | | 9.37 | 25.23 | 2 | 2 |
| Q13404 | Ubiquitin-conjugating enz | 4.37 | 10.88 | 1 | 1 | 4.23 | 14.29 | 1 | 1 | | 0.00 | | |
| Q12905 | Interleukin enhancer-binc | 14.15 | 18.21 | 3 | 3 | 14.35 | 20.00 | 3 | 3 | 24.99 | 20.00 | 3 | 5 |
| P09110 | 3-ketoacyl-CoA thiolase, | 12.82 | 15.09 | 3 | 3 | 20.27 | 20.05 | 4 | 4 | 25.29 | 25.00 | 5 | 5 |
| Q16836 | Hydroxyacyl-coenzyme A | 6.88 | 9.55 | 1 | 1 | 21.04 | 24.84 | 3 | 4 | | 0.00 | | |
| P06744 | Glucose-6-phosphate isor | 39.87 | 15.95 | 5 | 8 | 27.74 | 16.49 | 5 | 6 | 24.65 | 14.52 | 5 | 5 |
| P40926 | Malate dehydrogenase, n | 39.07 | 24.56 | 5 | 8 | 54.81 | 24.56 | 5 | 11 | 52.96 | 24.56 | 5 | 10 |
| P0CG06 | Ig lambda-3 chain C regi | 4.85 | 24.53 | 1 | 1 | | 0.00 | | | | 0.00 | | |
| P52943 | Cysteine-rich protein 2 OS=Homo sap | | 0.00 | | | 6.99 | 15.38 | 1 | 1 | 11.63 | 24.52 | 2 | 2 |
| Q9Y2V2 | Calcium-regulated heat stable protein | | 0.00 | | | | 0.00 | | | 5.84 | 24.49 | 1 | 1 |
| P11021 | 78 kDa glucose-regulatec | 49.50 | 20.34 | 8 | 10 | 59.38 | 17.28 | 8 | 12 | 29.15 | 9.79 | 5 | 6 |
| Q9UKY7 | Protein CDV3 homolog O | 11.28 | 24.42 | 2 | 2 | 12.03 | 18.99 | 1 | 2 | | 0.00 | | |
| Q9Y5U9 | Immediate early respons | 4.68 | 24.39 | 1 | 1 | | 0.00 | | | | | | |
| Q96QR8 | Transcriptional activator | 9.40 | 14.10 | 2 | 2 | 6.36 | 9.94 | 1 | 1 | 12.05 | 20.19 | 2 | 2 |
| P24752 | Acetyl-CoA acetyltransfer | 20.08 | 17.56 | 4 | 4 | 19.37 | 18.03 | 4 | 4 | 4.61 | 3.04 | 1 | 1 |
| Q9UNZ2 | NSFL1 cofactor p47 OS=I | 9.39 | 10.54 | 2 | 2 | 13.30 | 15.41 | 3 | 3 | 9.52 | 10.81 | 2 | 2 |
| P28070 | Proteasome subunit beta | 16.41 | 24.24 | 3 | 3 | 14.59 | 15.15 | 2 | 3 | 11.02 | 16.67 | 2 | 2 |
| P27797 | Calreticulin OS=Homo sa | 19.98 | 23.98 | 4 | 4 | 9.40 | 10.31 | 2 | 2 | 13.94 | 17.27 | 3 | 3 |
| P00558 | Phosphoglycerate kinase | 39.29 | 24.22 | 6 | 8 | 32.97 | 17.27 | 4 | 6 | 39.89 | 17.27 | 4 | 8 |
| P22314 | Ubiquitin-like modifier-act | 51.36 | 16.82 | 10 | 11 | 47.62 | 17.77 | 10 | 10 | 50.28 | 16.16 | 9 | 10 |
| Q99714 | 3-hydroxyacyl-CoA dehyd | 10.81 | 16.48 | 2 | 2 | 16.18 | 16.48 | 2 | 3 | 12.02 | 16.48 | 2 | 2 |
| P25705 | ATP synthase subunit alp | 32.87 | 17.90 | 6 | 7 | 31.90 | 21.70 | 7 | 7 | 28.77 | 16.46 | 5 | 6 |
| P40939 | Trifunctional enzyme sub | 26.35 | 12.71 | 5 | 6 | 21.58 | 9.96 | 5 | 5 | 38.31 | 13.50 | 6 | 8 |

| | | | | | | | | | | | | | |
|---|---|---|---|---|---|---|---|---|---|---|---|---|---|
| P17987 | T-complex protein 1 subu | 28.96 | 17.09 | 5 | 6 | 25.43 | 13.13 | 4 | 5 | 30.34 | 16.37 | 5 | 6 |
| P15531 | Nucleoside diphosphate k | 9.62 | 23.68 | 2 | 2 | | 0.00 | | | | 0.00 | | |
| P25713 | Metallothionein-3 OS=Homo sapiens ( | 0.00 | | | | 3.94 | 23.53 | 1 | 1 | | 0.00 | | |
| Q13185 | Chromobox protein homo | 8.66 | 15.85 | 2 | 2 | 9.46 | 16.39 | 2 | 2 | 15.35 | 23.50 | 3 | 3 |
| Q13405 | 39S ribosomal protein L4 | 9.34 | 16.27 | 2 | 2 | 13.76 | 23.49 | 3 | 3 | 4.93 | 7.83 | 1 | 1 |
| P42765 | 3-ketoacyl-CoA thiolase, | 9.22 | 11.08 | 2 | 2 | 4.85 | 7.05 | 1 | 1 | 4.98 | 5.04 | 1 | 1 |
| P62258 | 14-3-3 protein epsilon OS | 14.84 | 23.14 | 3 | 3 | 5.22 | 7.45 | 1 | 1 | 17.01 | 14.90 | 2 | 3 |
| Q13263 | Transcription intermediar | 33.30 | 13.77 | 6 | 7 | 19.95 | 12.57 | 4 | 4 | 19.69 | 10.90 | 4 | 4 |
| P05388 | 60S acidic ribosomal prot | 23.34 | 23.03 | 4 | 5 | 10.75 | 9.78 | 1 | 2 | 17.09 | 15.14 | 2 | 3 |
| P07355 | Annexin A2 OS=Homo sa | 19.89 | 13.86 | 4 | 4 | 23.23 | 22.71 | 5 | 5 | 25.32 | 14.75 | 4 | 5 |
| P10599 | Thioredoxin OS=Homo sa | 8.02 | 22.86 | 2 | 2 | 3.93 | 12.38 | 1 | 1 | | 0.00 | | |
| P62263 | 40S ribosomal protein S1 | 8.47 | 22.52 | 2 | 2 | 4.37 | 8.61 | 1 | 1 | 14.25 | 22.52 | 3 | 3 |
| P35579 | Myosin-9 OS=Homo sapi | 166.33 | 18.47 | 26 | 33 | 130.17 | 14.95 | 21 | 27 | 143.59 | 17.96 | 23 | 28 |
| Q16718 | NADH dehydrogenase [ubiquinone] 1 | 0.00 | | | | 5.37 | 22.41 | 1 | 1 | | 0.00 | | |
| Q04837 | Single-stranded DNA-binc | 8.88 | 22.30 | 2 | 2 | 9.43 | 22.30 | 2 | 2 | 9.62 | 22.30 | 2 | 2 |
| Q9Y281 | Cofilin-2 OS=Homo sapie | 5.49 | 10.24 | 1 | 1 | 4.50 | 10.24 | 1 | 1 | 14.06 | 22.29 | 2 | 3 |
| P47985 | Cytochrome b-c1 complex | 15.28 | 12.77 | 2 | 3 | 10.80 | 7.66 | 1 | 2 | 9.16 | 17.15 | 2 | 2 |
| P09661 | U2 small nuclear ribonucl | 10.05 | 15.29 | 2 | 2 | 9.88 | 15.29 | 2 | 2 | 10.12 | 12.16 | 2 | 2 |
| Q9UJZ1 | Stomatin-like protein 2, n | 15.30 | 17.70 | 3 | 3 | 14.24 | 17.13 | 3 | 3 | 10.18 | 8.99 | 2 | 2 |
| P29692 | Elongation factor 1-delta | 16.15 | 21.71 | 4 | 4 | 18.10 | 21.71 | 4 | 4 | 10.25 | 13.17 | 2 | 2 |
| Q99497 | Protein DJ-1 OS=Homo s | 14.11 | 21.69 | 2 | 3 | 9.24 | 21.69 | 2 | 2 | 10.20 | 21.69 | 2 | 2 |
| P63220 | 40S ribosomal protein S21 OS=Homo | 0.00 | | | | 5.30 | 21.69 | 1 | 1 | | 0.00 | | |
| O75363 | Breast carcinoma-amplifie | 23.93 | 17.12 | 5 | 5 | 30.94 | 15.41 | 4 | 6 | 13.93 | 7.53 | 3 | 3 |
| P13674 | Prolyl 4-hydroxylase subu | 28.43 | 18.91 | 5 | 6 | 20.40 | 9.93 | 3 | 4 | 4.17 | 2.62 | 1 | 1 |
| P06748 | Nucleophosmin OS=Hom | 19.05 | 21.43 | 4 | 4 | 26.86 | 16.67 | 3 | 5 | 15.15 | 21.43 | 3 | 3 |
| P52907 | F-actin-capping protein su | 8.22 | 12.24 | 2 | 2 | 15.53 | 15.73 | 2 | 3 | 9.60 | 14.69 | 2 | 2 |
| O00193 | Small acidic protein OS=H | 14.81 | 8.74 | 1 | 3 | 4.34 | 8.74 | 1 | 1 | 10.38 | 21.31 | 2 | 2 |
| P51149 | Ras-related protein Rab-7 | 4.33 | 7.73 | 1 | 1 | 4.44 | 6.76 | 1 | 1 | 8.92 | 13.53 | 2 | 2 |
| P61586 | Transforming protein Rho | 10.66 | 21.24 | 2 | 2 | 5.52 | 8.81 | 1 | 1 | 15.77 | 21.24 | 2 | 3 |
| O43678 | NADH dehydrogenase [ul | 4.25 | 21.21 | 1 | 1 | 3.94 | 21.21 | 1 | 1 | 4.47 | 21.21 | 1 | 1 |
| P12004 | Proliferating cell nuclear a | 8.70 | 16.09 | 2 | 2 | 9.93 | 12.26 | 2 | 2 | 5.11 | 7.28 | 1 | 1 |
| P0CG48 | Polyubiquitin-C OS=Homo | 8.68 | 21.02 | 1 | 2 | 4.79 | 21.02 | 1 | 1 | 4.79 | 21.02 | 1 | 1 |
| P60174 | Triosephosphate isomera | 22.45 | 15.38 | 5 | 5 | 21.17 | 20.98 | 5 | 5 | 14.12 | 15.38 | 3 | 3 |
| O75340 | Programmed cell death p | 4.74 | 14.14 | 1 | 1 | 5.16 | 14.14 | 1 | 1 | 8.68 | 20.94 | 2 | 2 |
| P13693 | Translationally-controlled | 5.02 | 12.79 | 1 | 1 | 9.25 | 20.93 | 2 | 2 | 15.62 | 12.79 | 1 | 3 |
| P08729 | Keratin, type II cytoskele | 37.75 | 18.55 | 6 | 8 | 28.08 | 15.99 | 5 | 6 | 30.30 | 18.55 | 6 | 6 |
| P48643 | T-complex protein 1 subu | 11.04 | 8.69 | 2 | 2 | 8.21 | 6.84 | 2 | 2 | 19.24 | 11.65 | 3 | 4 |
| O43399 | Tumor protein D54 OS=H | 9.62 | 20.87 | 2 | 2 | 9.29 | 20.87 | 2 | 2 | 5.14 | 9.22 | 1 | 1 |
| P49327 | Fatty acid synthase OS=H | 202.63 | 13.94 | 20 | 41 | 171.15 | 16.61 | 26 | 36 | 127.40 | 13.14 | 22 | 26 |

| | | | | | | | | | | | | | |
|---|---|---|---|---|---|---|---|---|---|---|---|---|---|
| P49915 | GMP synthase [glutamine | 24.05 | 15.87 | 4 | 5 | 11.38 | 7.07 | 2 | 2 | 18.72 | 9.96 | 4 | 4 |
| P07900 | Heat shock protein HSP 9 | 49.00 | 18.58 | 8 | 11 | 26.72 | 7.79 | 4 | 6 | 45.65 | 17.21 | 7 | 9 |
| P46778 | 60S ribosomal protein L2 | 9.12 | 20.63 | 2 | 2 | 4.94 | 9.38 | 1 | 1 | 10.11 | 20.63 | 2 | 2 |
| O95292 | Vesicle-associated memb | 4.47 | 8.23 | 1 | 1 | 12.28 | 20.58 | 3 | 3 | 4.68 | 6.58 | 1 | 1 |
| O14818 | Proteasome subunit alpha | 9.11 | 11.69 | 2 | 2 | 9.13 | 6.05 | 1 | 2 | 9.54 | 14.92 | 2 | 2 |
| P60660 | Myosin light polypeptide ( | 9.54 | 20.53 | 2 | 2 | 18.15 | 20.53 | 2 | 4 | 8.85 | 20.53 | 2 | 2 |
| P24534 | Elongation factor 1-beta OS=Homo sa | | 0.00 | | | 9.43 | 12.44 | 2 | 2 | 10.02 | 14.67 | 2 | 2 |
| Q15233 | Non-POU domain-contain | 15.17 | 15.50 | 3 | 3 | 5.52 | 4.67 | 1 | 1 | 11.42 | 9.55 | 2 | 2 |
| P98179 | Putative RNA-binding protein 3 OS=Ho | | 0.00 | | | 4.81 | 20.38 | 1 | 1 | | 0.00 | | |
| P61019 | Ras-related protein Rab-2 | 4.09 | 6.13 | 1 | 1 | 4.23 | 6.60 | 1 | 1 | 5.74 | 7.55 | 1 | 1 |
| P50991 | T-complex protein 1 subu | 17.12 | 14.47 | 4 | 4 | 12.77 | 9.65 | 3 | 3 | 23.45 | 17.63 | 5 | 5 |
| P42167 | Lamina-associated polype | 15.70 | 16.08 | 3 | 3 | | 0.00 | | | 9.43 | 3.96 | 2 | 2 |
| Q99798 | Aconitate hydratase, mito | 22.20 | 14.10 | 5 | 5 | 17.92 | 8.08 | 4 | 4 | 17.04 | 7.69 | 4 | 4 |
| P60981 | Destrin OS=Homo sapien | 13.32 | 20.00 | 2 | 3 | 9.52 | 9.70 | 1 | 2 | 4.68 | 9.70 | 1 | 1 |
| Q9Y333 | U6 snRNA-associated Sm-like protein I | | 0.00 | | | | 0.00 | | | 4.18 | 20.00 | 1 | 1 |
| Q9NVJ2 | ADP-ribosylation factor-lik | 4.14 | 7.53 | 1 | 1 | 3.94 | 7.53 | 1 | 1 | 5.09 | 12.37 | 1 | 1 |
| P14866 | Heterogeneous nuclear ri | 18.51 | 13.92 | 4 | 4 | 9.69 | 7.13 | 2 | 2 | 11.10 | 8.66 | 2 | 2 |
| P55072 | Transitional endoplasmic | 37.22 | 15.63 | 7 | 8 | 27.57 | 10.67 | 5 | 6 | 29.64 | 14.89 | 6 | 6 |
| P30101 | Protein disulfide-isomeras | 9.45 | 7.13 | 2 | 2 | 10.08 | 6.14 | 2 | 2 | 28.59 | 19.80 | 6 | 6 |
| Q16891 | MICOS complex subunit M | 13.56 | 6.60 | 3 | 3 | 12.18 | 6.07 | 3 | 3 | 14.94 | 10.82 | 3 | 3 |
| Q7KZF4 | Staphylococcal nuclease d | 61.64 | 19.78 | 9 | 12 | 41.77 | 12.86 | 6 | 8 | 27.07 | 10.55 | 4 | 5 |
| P26885 | Peptidyl-prolyl cis-trans isomerase FKE | | 0.00 | | | | 0.00 | | | 5.22 | 19.72 | 1 | 1 |
| Q9BUP0 | EF-hand domain-containi | 11.35 | 12.55 | 2 | 2 | 4.33 | 7.11 | 1 | 1 | 4.56 | 7.11 | 1 | 1 |
| P12532 | Creatine kinase U-type, m | 25.70 | 19.66 | 4 | 5 | 5.88 | 5.04 | 1 | 1 | 16.51 | 11.27 | 2 | 3 |
| P61513 | 60S ribosomal protein L3 | 9.26 | 19.57 | 1 | 2 | | 0.00 | | | 4.16 | 19.57 | 1 | 1 |
| Q6DRA6 | Putative histone H2B type | 4.46 | 9.15 | 1 | 1 | | 0.00 | | | 4.13 | 10.37 | 1 | 1 |
| P58107 | Epiplakin OS=Homo sapie | 107.82 | 19.51 | 17 | 21 | 64.15 | 12.73 | 9 | 12 | 60.72 | 9.63 | 9 | 12 |
| P45880 | Voltage-dependent anion | 11.61 | 7.82 | 1 | 2 | 9.62 | 12.59 | 2 | 2 | 15.08 | 14.63 | 2 | 3 |
| O75874 | Isocitrate dehydrogenase | 9.20 | 7.97 | 2 | 2 | 8.53 | 6.76 | 2 | 2 | 13.62 | 12.80 | 3 | 3 |
| Q9HB71 | Calcyclin-binding protein | 8.59 | 19.30 | 2 | 2 | 6.08 | 10.53 | 1 | 1 | 16.28 | 19.30 | 2 | 3 |
| P29966 | Myristoylated alanine-rich | 11.52 | 19.28 | 2 | 2 | | 0.00 | | | 12.30 | 19.28 | 2 | 2 |
| Q06323 | Proteasome activator con | 20.39 | 19.28 | 3 | 4 | 9.64 | 13.65 | 2 | 2 | 10.30 | 13.65 | 2 | 2 |
| P41208 | Centrin-2 OS=Homo sapiens GN=CET | | 0.00 | | | | 0.00 | | | 5.41 | 19.19 | 1 | 1 |
| Q15084 | Protein disulfide-isomeras | 8.86 | 6.82 | 2 | 2 | 23.41 | 16.14 | 4 | 5 | 26.34 | 19.09 | 5 | 5 |
| Q13155 | Aminoacyl tRNA synthase complex-inte | | 0.00 | | | 4.81 | 7.81 | 1 | 1 | 6.74 | 11.25 | 1 | 1 |
| P28074 | Proteasome subunit beta | 13.85 | 19.01 | 3 | 3 | | 0.00 | | | 8.34 | 11.03 | 2 | 2 |
| Q8N6T3 | ADP-ribosylation factor G | 10.27 | 14.04 | 2 | 2 | 4.47 | 3.69 | 1 | 1 | 16.50 | 8.62 | 2 | 3 |
| O43598 | 2'-deoxynucleoside 5'-pho | 5.34 | 18.97 | 1 | 1 | | 0.00 | | | | 0.00 | | |
| Q9UBW8 | COP9 signalosome compl | 9.41 | 10.91 | 2 | 2 | | 0.00 | | | 5.19 | 8.00 | 1 | 1 |

| | | | | | | | | | | | | | |
|---|---|---|---|---|---|---|---|---|---|---|---|---|---|
| P08195 | 4F2 cell-surface antigen h | 10.32 | 6.83 | 2 | 2 | 12.73 | 8.10 | 3 | 3 | 26.60 | 11.59 | 4 | 5 |
| P08107 | Heat shock 70 kDa protei | 20.64 | 12.32 | 4 | 4 | 43.14 | 18.56 | 6 | 8 | 46.84 | 18.88 | 7 | 9 |
| P02545 | Prelamin-A/C OS=Homo s | 32.18 | 17.17 | 7 | 7 | 33.57 | 15.21 | 6 | 7 | 38.63 | 16.87 | 7 | 8 |
| P35270 | Sepiapterin reductase OS | 9.62 | 13.03 | 2 | 2 | 9.41 | 13.03 | 2 | 2 | 9.32 | 13.03 | 2 | 2 |
| P35232 | Prohibitin OS=Homo sapi | 3.91 | 5.15 | 1 | 1 | 15.96 | 13.60 | 2 | 3 | 8.94 | 11.40 | 2 | 2 |
| P15374 | Ubiquitin carboxyl-termin | 7.74 | 13.04 | 2 | 2 | 12.69 | 11.74 | 2 | 3 | 4.33 | 6.96 | 1 | 1 |
| P07339 | Cathepsin D OS=Homo sa | 17.76 | 15.53 | 4 | 4 | 17.46 | 16.02 | 4 | 4 | 13.84 | 11.89 | 3 | 3 |
| O00299 | Chloride intracellular char | 10.97 | 18.67 | 2 | 2 | 10.70 | 18.67 | 2 | 2 | 10.47 | 18.67 | 2 | 2 |
| O75947 | ATP synthase subunit d, r | 10.19 | 9.94 | 1 | 2 | 4.41 | 9.94 | 1 | 1 | 9.30 | 18.63 | 2 | 2 |
| Q9H3K6 | BolA-like protein 2 OS=H | 3.88 | 18.60 | 1 | 1 | 4.77 | 18.60 | 1 | 1 | 5.13 | 18.60 | 1 | 1 |
| P31939 | Bifunctional purine biosyr | 17.92 | 13.18 | 4 | 4 | 19.49 | 11.15 | 4 | 4 | 14.63 | 9.63 | 3 | 3 |
| P63173 | 60S ribosomal protein L38 OS=Homo | 0.00 | | | | 9.02 | 18.57 | 1 | 2 | | 0.00 | | |
| P13645 | Keratin, type I cytoskeleta | 56.32 | 18.49 | 7 | 10 | 37.13 | 17.47 | 6 | 7 | 21.49 | 11.13 | 3 | 4 |
| Q99653 | Calcineurin B homologous | 3.95 | 8.21 | 1 | 1 | 9.63 | 10.26 | 1 | 2 | | 0.00 | | |
| Q96FQ6 | Protein S100-A16 OS=Ho | 4.91 | 18.45 | 1 | 1 | 4.36 | 18.45 | 1 | 1 | 8.75 | 18.45 | 1 | 2 |
| Q9Y3D7 | Mitochondrial import inne | 5.02 | 18.40 | 1 | 1 | 4.63 | 18.40 | 1 | 1 | | 0.00 | | |
| P18754 | Regulator of chromosome | 10.71 | 11.40 | 2 | 2 | | 0.00 | | | 17.83 | 18.29 | 3 | 3 |
| P14174 | Macrophage migration inl | 4.16 | 18.26 | 1 | 1 | 4.72 | 18.26 | 1 | 1 | 4.34 | 18.26 | 1 | 1 |
| O95336 | 6-phosphogluconolactona | 5.83 | 8.14 | 1 | 1 | | 0.00 | | | 9.70 | 18.22 | 2 | 2 |
| O95833 | Chloride intracellular channel protein 3 | 0.00 | | | | 8.88 | 18.22 | 2 | 2 | | 0.00 | | |
| P43490 | Nicotinamide phosphoribo | 30.87 | 18.13 | 5 | 6 | 11.84 | 7.13 | 2 | 2 | 27.19 | 15.07 | 4 | 5 |
| Q8WXX5 | DnaJ homolog subfamily | 4.73 | 7.69 | 1 | 1 | 5.26 | 10.38 | 1 | 1 | 10.73 | 18.08 | 2 | 2 |
| Q99623 | Prohibitin-2 OS=Homo sa | 19.08 | 18.06 | 4 | 4 | 14.15 | 14.72 | 3 | 3 | 10.69 | 10.70 | 2 | 2 |
| O43752 | Syntaxin-6 OS=Homo sap | 5.14 | 9.80 | 1 | 1 | | 0.00 | | | 4.96 | 8.24 | 1 | 1 |
| P16435 | NADPH--cytochrome P450 | 21.63 | 9.01 | 3 | 4 | 9.64 | 5.91 | 2 | 2 | 24.61 | 11.82 | 4 | 5 |
| Q92597 | Protein NDRG1 OS=Homo | 9.42 | 8.12 | 2 | 2 | 8.41 | 7.61 | 2 | 2 | 9.82 | 10.41 | 2 | 2 |
| P25787 | Proteasome subunit alpha type-2 OS= | 0.00 | | | | 9.48 | 17.95 | 2 | 2 | 10.25 | 17.95 | 2 | 2 |
| Q15369 | Transcription elongation f | 5.17 | 17.86 | 1 | 1 | 9.95 | 17.86 | 1 | 2 | 10.73 | 17.86 | 1 | 2 |
| P34932 | Heat shock 70 kDa protei | 38.45 | 13.81 | 7 | 8 | 24.65 | 11.90 | 5 | 5 | 17.62 | 6.79 | 3 | 3 |
| P83916 | Chromobox protein homo | 4.42 | 8.65 | 1 | 1 | 4.53 | 8.65 | 1 | 1 | 9.09 | 17.84 | 2 | 2 |
| O00429 | Dynamin-1-like protein O | 26.43 | 14.81 | 5 | 5 | 24.26 | 10.46 | 4 | 5 | 10.22 | 5.16 | 2 | 2 |
| P05091 | Aldehyde dehydrogenase | 14.85 | 9.86 | 3 | 3 | 18.08 | 11.80 | 4 | 4 | 24.69 | 12.77 | 4 | 5 |
| Q07021 | Complement component | 9.93 | 17.73 | 2 | 2 | 4.49 | 7.09 | 1 | 1 | 4.67 | 7.09 | 1 | 1 |
| P18669 | Phosphoglycerate mutase | 15.78 | 17.72 | 2 | 3 | | 0.00 | | | 10.90 | 7.09 | 1 | 2 |
| P31942 | Heterogeneous nuclear ri | 4.40 | 4.91 | 1 | 1 | 14.07 | 17.63 | 3 | 3 | 4.96 | 4.91 | 1 | 1 |
| Q08257 | Quinone oxidoreductase OS=Homo sa | 0.00 | | | | 5.02 | 8.21 | 1 | 1 | 5.93 | 9.42 | 1 | 1 |
| P27695 | DNA-(apurinic or apyrimic | 9.03 | 10.69 | 2 | 2 | 9.57 | 12.26 | 2 | 2 | 19.64 | 17.61 | 3 | 4 |
| P18859 | ATP synthase-coupling fa | 4.39 | 17.59 | 1 | 1 | 5.12 | 17.59 | 1 | 1 | 9.56 | 17.59 | 1 | 2 |
| P12814 | Alpha-actinin-1 OS=Homo | 33.48 | 10.76 | 6 | 7 | 13.89 | 4.93 | 3 | 3 | 40.22 | 12.67 | 7 | 8 |

| | | | | | | | | | | | | | |
|---|---|---|---|---|---|---|---|---|---|---|---|---|---|
| P00966 | Argininosuccinate synthase OS=Homo | 0.00 | | | 40.37 | 17.48 | 4 | 8 | 19.36 | 11.89 | 3 | 4 |
| P82909 | 28S ribosomal protein S3 | 4.65 | 17.48 | 1 | 1 | 4.16 | 17.48 | 1 | 1 | 4.49 | 17.48 | 1 | 1 |
| Q96KP4 | Cytosolic non-specific dip | 4.07 | 2.95 | 1 | 1 | 13.53 | 9.89 | 3 | 3 | 13.74 | 10.53 | 3 | 3 |
| P09651 | Heterogeneous nuclear ri | 10.44 | 4.57 | 2 | 2 | 18.21 | 12.90 | 4 | 4 | 28.00 | 17.20 | 4 | 6 |
| Q9NRV9 | Heme-binding protein 1 ( | 8.51 | 17.46 | 2 | 2 | 5.17 | 8.99 | 1 | 1 | 5.40 | 8.99 | 1 | 1 |
| O75369 | Filamin-B OS=Homo sapi | 73.58 | 11.53 | 16 | 16 | 60.79 | 8.22 | 12 | 13 | 62.33 | 9.92 | 11 | 12 |
| P00761 | Trypsin OS=Sus scrofa PI | 31.68 | 17.32 | 2 | 6 | 24.00 | 8.66 | 1 | 4 | 16.11 | 17.32 | 2 | 3 |
| P46940 | Ras GTPase-activating-lik | 55.10 | 11.59 | 11 | 11 | 32.11 | 7.18 | 6 | 6 | 50.80 | 9.72 | 9 | 10 |
| P99999 | Cytochrome c OS=Homo sapiens GN= | 0.00 | | | | 0.00 | | | 4.26 | 17.14 | 1 | 1 |
| P20042 | Eukaryotic translation init | 4.38 | 6.61 | 1 | 1 | 4.05 | 4.50 | 1 | 1 | 5.50 | 6.01 | 1 | 1 |
| Q9Y6U3 | Adseverin OS=Homo sap | 19.70 | 9.51 | 4 | 4 | 25.03 | 11.33 | 5 | 5 | 20.06 | 10.49 | 4 | 4 |
| Q8NCW5 | NAD(P)H-hydrate epimer | 12.16 | 17.01 | 3 | 3 | 4.35 | 6.25 | 1 | 1 | 4.41 | 6.60 | 1 | 1 |
| Q15836 | Vesicle-associated memb | 5.49 | 17.00 | 1 | 1 | 4.58 | 17.00 | 1 | 1 | 4.42 | 17.00 | 1 | 1 |
| P62937 | Peptidyl-prolyl cis-trans is | 13.90 | 16.97 | 2 | 3 | 8.70 | 16.97 | 2 | 2 | 4.24 | 9.09 | 1 | 1 |
| Q8N4Q1 | Mitochondrial intermembrane space in | 0.00 | | | | 0.00 | | | 4.86 | 16.90 | 1 | 1 |
| P08238 | Heat shock protein HSP 9 | 61.46 | 16.71 | 8 | 14 | 36.94 | 10.50 | 5 | 8 | 26.95 | 10.77 | 6 | 6 |
| Q9Y5J7 | Mitochondrial import inner membrane | 0.00 | | | | 0.00 | | | 4.91 | 16.85 | 1 | 1 |
| P63244 | Guanine nucleotide-bindir | 4.94 | 5.05 | 1 | 1 | 14.71 | 16.72 | 3 | 3 | 10.03 | 12.62 | 2 | 2 |
| Q9Y4L1 | Hypoxia up-regulated pro | 34.26 | 11.11 | 6 | 7 | 34.87 | 9.81 | 5 | 6 | 25.05 | 9.21 | 5 | 5 |
| O75347 | Tubulin-specific chaperon | 11.04 | 16.67 | 2 | 2 | 4.10 | 13.89 | 1 | 1 | 11.06 | 16.67 | 2 | 2 |
| P01767 | Ig heavy chain V-III region BUT OS=H | 0.00 | | | | 0.00 | | | 4.57 | 16.52 | 1 | 1 |
| P36873 | Serine/threonine-protein | 8.17 | 11.15 | 2 | 2 | 13.24 | 9.60 | 2 | 3 | 4.86 | 5.26 | 1 | 1 |
| P30044 | Peroxiredoxin-5, mitochor | 4.64 | 8.41 | 1 | 1 | 9.17 | 16.36 | 2 | 2 | | 0.00 | | |
| Q9BPX5 | Actin-related protein 2/3 | 3.89 | 7.84 | 1 | 1 | 4.26 | 8.50 | 1 | 1 | 4.13 | 8.50 | 1 | 1 |
| Q15019 | Septin-2 OS=Homo sapie | 4.67 | 4.43 | 1 | 1 | 9.05 | 11.63 | 2 | 2 | 8.36 | 9.14 | 2 | 2 |
| Q9H910 | Hematological and neurol | 4.34 | 8.95 | 1 | 1 | 4.20 | 7.37 | 1 | 1 | | 0.00 | | |
| P28072 | Proteasome subunit beta | 6.12 | 16.32 | 1 | 1 | | 0.00 | | | | 0.00 | | |
| P12956 | X-ray repair cross-comple | 9.42 | 5.42 | 2 | 2 | 5.13 | 2.30 | 1 | 1 | 22.17 | 13.14 | 5 | 5 |
| P07476 | Involucrin OS=Homo sapiens GN=IVL | 0.00 | | | 4.88 | 3.42 | 1 | 1 | 29.05 | 16.24 | 5 | 6 |
| P62140 | Serine/threonine-protein | 8.78 | 11.01 | 2 | 2 | 13.24 | 9.48 | 2 | 3 | 4.86 | 5.20 | 1 | 1 |
| P02656 | Apolipoprotein C-III OS= | 5.07 | 16.16 | 1 | 1 | 4.72 | 16.16 | 1 | 1 | 5.32 | 16.16 | 1 | 1 |
| Q04323 | UBX domain-containing protein 1 OS= | 0.00 | | | 4.78 | 9.76 | 1 | 1 | 5.31 | 6.40 | 1 | 1 |
| Q9H299 | SH3 domain-binding gluta | 5.23 | 16.13 | 1 | 1 | 4.70 | 16.13 | 1 | 1 | 5.02 | 16.13 | 1 | 1 |
| P62316 | Small nuclear ribonucleoprotein Sm D2 | 0.00 | | | 4.29 | 15.25 | 1 | 1 | 4.44 | 16.10 | 1 | 1 |
| P48735 | Isocitrate dehydrogenase | 25.13 | 15.93 | 5 | 5 | 14.77 | 9.73 | 3 | 3 | 9.70 | 6.64 | 2 | 2 |
| P13804 | Electron transfer flavopro | 4.67 | 5.71 | 1 | 1 | 4.63 | 5.41 | 1 | 1 | 4.15 | 4.80 | 1 | 1 |
| P06396 | Gelsolin OS=Homo sapier | 26.95 | 13.55 | 6 | 6 | 15.53 | 7.29 | 3 | 3 | 20.12 | 10.36 | 4 | 4 |
| P09429 | High mobility group prote | 5.68 | 8.84 | 1 | 1 | 9.27 | 15.81 | 2 | 2 | 4.54 | 6.98 | 1 | 1 |
| A8MWD9 | Putative small nuclear rib | 4.36 | 15.79 | 1 | 1 | 4.01 | 15.79 | 1 | 1 | | 0.00 | | |

| | | | | | | | | | | | | | |
|---|---|---|---|---|---|---|---|---|---|---|---|---|---|
| Q6IBS0 | Twinfilin-2 OS=Homo sap | 13.62 | 15.76 | 3 | 3 | 5.01 | 5.44 | 1 | 1 | 9.59 | 10.89 | 2 | 2 |
| P13010 | X-ray repair cross-comple | 14.83 | 9.70 | 3 | 3 | 19.05 | 9.02 | 3 | 4 | 4.11 | 3.28 | 1 | 1 |
| P42166 | Lamina-associated polype | 15.07 | 10.23 | 3 | 3 | | 0.00 | | | 13.78 | 5.48 | 3 | 3 |
| P60953 | Cell division control prote | 14.16 | 15.71 | 2 | 3 | 9.55 | 8.90 | 1 | 2 | 10.68 | 8.90 | 1 | 2 |
| P14550 | Alcohol dehydrogenase [I | 13.69 | 11.38 | 3 | 3 | 18.78 | 15.69 | 4 | 4 | 4.85 | 5.85 | 1 | 1 |
| P52565 | Rho GDP-dissociation inh | 14.37 | 15.69 | 3 | 3 | 4.44 | 7.35 | 1 | 1 | 9.91 | 15.20 | 2 | 2 |
| Q9Y2T7 | Y-box-binding protein 2 ( | 22.33 | 15.66 | 3 | 5 | 3.93 | 4.67 | 1 | 1 | 13.91 | 8.79 | 2 | 3 |
| P02666 | Beta-casein - Bos taurus | 20.92 | 15.63 | 2 | 4 | 26.04 | 15.63 | 2 | 5 | 20.53 | 15.63 | 2 | 4 |
| Q15907 | Ras-related protein Rab-1 | 4.20 | 5.96 | 1 | 1 | 9.32 | 15.60 | 2 | 2 | 5.19 | 9.63 | 1 | 1 |
| P63241 | Eukaryotic translation init | 21.92 | 15.58 | 2 | 4 | 17.05 | 15.58 | 2 | 3 | 27.94 | 15.58 | 2 | 5 |
| Q00169 | Phosphatidylinositol trans | 4.78 | 7.04 | 1 | 1 | 4.35 | 7.04 | 1 | 1 | 10.36 | 8.52 | 1 | 2 |
| Q9H6S3 | Epidermal growth factor r | 20.35 | 10.49 | 3 | 4 | 19.26 | 10.21 | 3 | 4 | 30.26 | 15.52 | 5 | 6 |
| P42677 | 40S ribosomal protein S2 | 3.86 | 15.48 | 1 | 1 | | 0.00 | | | | 0.00 | | |
| P22695 | Cytochrome b-c1 complex | 15.26 | 11.70 | 3 | 3 | 17.42 | 11.48 | 3 | 4 | 10.97 | 7.51 | 2 | 2 |
| P61457 | Pterin-4-alpha-carbinolamine dehydrat | | 0.00 | | | 4.51 | 15.38 | 1 | 1 | 4.90 | 15.38 | 1 | 1 |
| O75396 | Vesicle-trafficking protein | 9.37 | 15.35 | 2 | 2 | 4.56 | 8.84 | 1 | 1 | 13.93 | 15.35 | 2 | 3 |
| Q9NYL9 | Tropomodulin-3 OS=Hom | 5.10 | 5.97 | 1 | 1 | 8.14 | 9.94 | 2 | 2 | 5.53 | 5.40 | 1 | 1 |
| P31949 | Protein S100-A11 OS=Homo sapiens ( | | 0.00 | | | 10.88 | 15.24 | 1 | 2 | | 0.00 | | |
| Q08380 | Galectin-3-binding protein | 27.28 | 15.21 | 5 | 6 | 4.35 | 2.39 | 1 | 1 | 5.85 | 3.25 | 1 | 1 |
| P62306 | Small nuclear ribonucleoprotein F OS= | | 0.00 | | | | | | | 4.76 | 15.12 | 1 | 1 |
| O76003 | Glutaredoxin-3 OS=Homo | 8.63 | 7.46 | 2 | 2 | | 0.00 | | | 5.15 | 7.46 | 1 | 1 |
| P09960 | Leukotriene A-4 hydrolase | 17.99 | 10.31 | 4 | 4 | 8.72 | 4.42 | 2 | 2 | 23.37 | 10.47 | 4 | 5 |
| P40227 | T-complex protein 1 subu | 24.00 | 14.88 | 4 | 5 | 28.64 | 14.88 | 4 | 6 | 14.58 | 7.91 | 2 | 3 |
| P02663 | Alpha-S2-casein [Contain | 20.38 | 14.86 | 2 | 4 | 11.63 | 9.91 | 1 | 2 | 18.09 | 9.91 | 1 | 3 |
| Q01105 | Protein SET OS=Homo sa | 19.19 | 11.03 | 2 | 4 | 11.03 | 10.00 | 2 | 2 | 10.39 | 10.00 | 2 | 2 |
| Q99807 | Ubiquinone biosynthesis protein COQ7 | | 0.00 | | | 4.79 | 14.75 | 1 | 1 | | 0.00 | | |
| Q9Y5L4 | Mitochondrial import inne | 5.21 | 14.74 | 1 | 1 | | 0.00 | | | 5.32 | 14.74 | 1 | 1 |
| P09525 | Annexin A4 OS=Homo sa | 14.13 | 14.73 | 3 | 3 | 8.98 | 10.03 | 2 | 2 | 4.91 | 5.02 | 1 | 1 |
| Q86U42 | Polyadenylate-binding protein 2 OS=H | | 0.00 | | | | 0.00 | | | 5.25 | 14.71 | 1 | 1 |
| Q15121 | Astrocytic phosphoprotein PEA-15 OS= | | 0.00 | | | | 0.00 | | | 4.58 | 14.62 | 1 | 1 |
| Q15691 | Microtubule-associated pr | 4.09 | 4.48 | 1 | 1 | 5.42 | 10.07 | 1 | 1 | | 0.00 | | |
| P25325 | 3-mercaptopyruvate sulfurtransferase | | 0.00 | | | 9.97 | 14.48 | 2 | 2 | 5.10 | 9.09 | 1 | 1 |
| P00338 | L-lactate dehydrogenase | 30.77 | 9.64 | 2 | 6 | 25.56 | 9.64 | 2 | 5 | 20.70 | 14.46 | 3 | 4 |
| P32322 | Pyrroline-5-carboxylate re | 12.27 | 9.09 | 2 | 3 | 8.52 | 10.97 | 2 | 2 | | 0.00 | | |
| Q9HCY8 | Protein S100-A14 OS=Ho | 5.01 | 14.42 | 1 | 1 | 5.53 | 14.42 | 1 | 1 | 4.55 | 14.42 | 1 | 1 |
| P58546 | Myotrophin OS=Homo sa | 6.24 | 14.41 | 1 | 1 | 10.75 | 14.41 | 1 | 2 | 5.01 | 14.41 | 1 | 1 |
| P23396 | 40S ribosomal protein S3 | 8.96 | 14.40 | 2 | 2 | 13.33 | 14.40 | 2 | 3 | 4.98 | 9.05 | 1 | 1 |
| Q14103 | Heterogeneous nuclear ri | 9.68 | 9.01 | 2 | 2 | 14.46 | 9.30 | 2 | 3 | 9.15 | 9.30 | 2 | 2 |
| Q15365 | Poly(rC)-binding protein 1 | 9.58 | 5.62 | 1 | 2 | 10.22 | 14.33 | 2 | 2 | 9.93 | 5.62 | 1 | 2 |

| | | | | | | | | | | | | | |
|---|---|---|---|---|---|---|---|---|---|---|---|---|---|
| P13995 | Bifunctional methylenetet | 8.85 | 14.29 | 2 | 2 | | 0.00 | | | | 0.00 | | |
| P21281 | V-type proton ATPase sul | 5.16 | 2.74 | 1 | 1 | 4.57 | 4.31 | 1 | 1 | 14.79 | 11.55 | 3 | 3 |
| P15559 | NAD(P)H dehydrogenase | 5.22 | 6.57 | 1 | 1 | 16.24 | 14.23 | 2 | 3 | 21.67 | 14.23 | 2 | 4 |
| O00629 | Importin subunit alpha-3 OS=Homo sa | 0.00 | | | | 15.28 | 11.52 | 3 | 3 | 21.12 | 10.94 | 3 | 4 |
| P50402 | Emerin OS=Homo sapien | 8.85 | 14.17 | 2 | 2 | | 0.00 | | | 4.81 | 5.91 | 1 | 1 |
| Q13283 | Ras GTPase-activating pr | 14.60 | 10.94 | 3 | 3 | 16.30 | 7.08 | 2 | 3 | 19.43 | 14.16 | 4 | 4 |
| P62333 | 26S protease regulatory s | 10.23 | 10.54 | 2 | 2 | 5.24 | 7.71 | 1 | 1 | 4.18 | 3.60 | 1 | 1 |
| Q9P2E9 | Ribosome-binding proteir | 52.44 | 11.91 | 9 | 11 | 43.53 | 10.07 | 8 | 9 | 41.67 | 7.73 | 5 | 8 |
| Q92905 | COP9 signalosome compl | 5.88 | 8.38 | 1 | 1 | | 0.00 | | | 10.91 | 5.69 | 1 | 2 |
| O95139 | NADH dehydrogenase [ubiquinone] 1 | 0.00 | | | | | 0.00 | | | 4.18 | 14.06 | 1 | 1 |
| P05386 | 60S acidic ribosomal prot | 3.94 | 14.04 | 1 | 1 | | 0.00 | | | | 0.00 | | |
| P51991 | Heterogeneous nuclear ri | 5.06 | 5.82 | 1 | 1 | 3.91 | 4.23 | 1 | 1 | 15.12 | 9.79 | 2 | 3 |
| Q9Y5M8 | Signal recognition particle | 9.45 | 14.02 | 2 | 2 | 9.28 | 14.02 | 2 | 2 | 5.57 | 7.01 | 1 | 1 |
| Q9Y3F4 | Serine-threonine kinase r | 9.78 | 8.86 | 2 | 2 | 14.87 | 10.29 | 2 | 3 | 5.32 | 5.14 | 1 | 1 |
| P05023 | Sodium/potassium-transp | 34.47 | 10.75 | 6 | 7 | 13.95 | 5.28 | 3 | 3 | 25.90 | 5.87 | 3 | 5 |
| P05198 | Eukaryotic translation init | 3.92 | 3.81 | 1 | 1 | | 0.00 | | | 9.53 | 10.16 | 2 | 2 |
| O75390 | Citrate synthase, mitocho | 13.37 | 6.65 | 2 | 3 | 4.17 | 3.43 | 1 | 1 | 14.14 | 13.95 | 3 | 3 |
| O95168 | NADH dehydrogenase [ul | 4.13 | 13.95 | 1 | 1 | | 0.00 | | | | 0.00 | | |
| Q9NZ45 | CDGSH iron-sulfur domai | 4.39 | 13.89 | 1 | 1 | | 0.00 | | | 4.53 | 13.89 | 1 | 1 |
| P28331 | NADH-ubiquinone oxidore | 23.43 | 11.55 | 5 | 5 | 8.21 | 4.54 | 2 | 2 | 24.57 | 11.28 | 5 | 5 |
| Q07020 | 60S ribosomal protein L1 | 8.63 | 13.83 | 2 | 2 | 4.69 | 6.91 | 1 | 1 | 8.64 | 13.83 | 2 | 2 |
| P55209 | Nucleosome assembly pr | 9.39 | 9.21 | 2 | 2 | 4.67 | 4.35 | 1 | 1 | 9.49 | 8.95 | 2 | 2 |
| Q15388 | Mitochondrial import rece | 11.46 | 13.79 | 1 | 2 | 5.65 | 13.79 | 1 | 1 | 5.24 | 13.79 | 1 | 1 |
| P07954 | Fumarate hydratase, mit | 15.82 | 10.78 | 3 | 3 | 13.47 | 9.22 | 3 | 3 | 15.88 | 8.04 | 2 | 3 |
| P16989 | Y-box-binding protein 3 ( | 23.05 | 13.71 | 3 | 5 | 3.93 | 4.57 | 1 | 1 | 18.74 | 13.71 | 3 | 4 |
| O14907 | Tax1-binding protein 3 OS=Homo sap | 0.00 | | | | 3.95 | 13.71 | 1 | 1 | 4.41 | 13.71 | 1 | 1 |
| P25815 | Protein S100-P OS=Hom | 4.27 | 13.68 | 1 | 1 | 4.19 | 13.68 | 1 | 1 | | 0.00 | | |
| Q9Y5Z4 | Heme-binding protein 2 ( | 4.33 | 7.32 | 1 | 1 | 8.90 | 13.66 | 2 | 2 | 4.31 | 6.34 | 1 | 1 |
| P31943 | Heterogeneous nuclear ri | 28.74 | 13.59 | 4 | 6 | 8.24 | 9.58 | 2 | 2 | 16.12 | 9.80 | 3 | 3 |
| Q9NP72 | Ras-related protein Rab-1 | 4.08 | 6.31 | 1 | 1 | 4.06 | 7.28 | 1 | 1 | 4.31 | 6.31 | 1 | 1 |
| O96008 | Mitochondrial import rece | 8.68 | 13.57 | 2 | 2 | | 0.00 | | | 4.36 | 4.16 | 1 | 1 |
| Q13509 | Tubulin beta-3 chain OS= | 24.36 | 13.56 | 4 | 5 | 20.11 | 10.89 | 3 | 4 | 14.71 | 10.89 | 3 | 3 |
| Q16204 | Coiled-coil domain-contai | 13.82 | 10.55 | 3 | 3 | 5.52 | 4.01 | 1 | 1 | 15.39 | 6.96 | 3 | 3 |
| O75531 | Barrier-to-autointegration factor OS=H | 0.00 | | | | 4.27 | 13.48 | 1 | 1 | | 0.00 | | |
| P62241 | 40S ribosomal protein S8 | 8.84 | 13.46 | 2 | 2 | 4.76 | 6.25 | 1 | 1 | 4.82 | 6.25 | 1 | 1 |
| P35908 | Keratin, type II cytoskele | 4.88 | 1.88 | 1 | 1 | 27.73 | 7.82 | 4 | 6 | 18.49 | 7.51 | 4 | 4 |
| Q00796 | Sorbitol dehydrogenase ( | 13.11 | 13.45 | 3 | 3 | | 0.00 | | | | 0.00 | | |
| Q04917 | 14-3-3 protein eta OS=H | 9.85 | 7.72 | 1 | 2 | 5.10 | 7.72 | 1 | 1 | 14.77 | 13.41 | 2 | 3 |
| P41250 | Glycine--tRNA ligase OS= | 30.01 | 13.40 | 5 | 6 | 10.21 | 5.55 | 2 | 2 | 19.86 | 11.64 | 4 | 4 |

| | | | | | | | | | | | | | |
|---|---|---|---|---|---|---|---|---|---|---|---|---|---|
| Q9H444 | Charged multivesicular bo | 4.25 | 6.25 | 1 | 1 | 4.71 | 7.14 | 1 | 1 | | 0.00 | | |
| P11177 | Pyruvate dehydrogenase | 8.03 | 8.91 | 2 | 2 | 9.29 | 8.91 | 2 | 2 | 5.11 | 4.46 | 1 | 1 |
| P61981 | 14-3-3 protein gamma OS | 10.99 | 13.36 | 2 | 2 | 10.51 | 13.36 | 2 | 2 | 8.87 | 13.36 | 2 | 2 |
| P62993 | Growth factor receptor-bo | 4.77 | 13.36 | 1 | 1 | | 0.00 | | | | 0.00 | | |
| Q9UJS0 | Calcium-binding mitochor | 20.95 | 10.81 | 4 | 4 | 19.76 | 10.52 | 4 | 4 | 9.93 | 5.19 | 2 | 2 |
| P18124 | 60S ribosomal protein L7 | 4.64 | 5.65 | 1 | 1 | | 0.00 | | | 4.58 | 7.66 | 1 | 1 |
| P49411 | Elongation factor Tu, mito | 4.91 | 3.98 | 1 | 1 | 13.31 | 10.62 | 3 | 3 | 17.29 | 10.18 | 3 | 4 |
| Q9Y2Q3 | Glutathione S-transferase | 8.87 | 13.27 | 2 | 2 | 8.89 | 13.27 | 2 | 2 | 9.49 | 13.27 | 2 | 2 |
| Q14257 | Reticulocalbin-2 OS=Hom | 10.26 | 7.26 | 1 | 2 | 5.61 | 5.99 | 1 | 1 | 5.43 | 7.26 | 1 | 1 |
| P05455 | Lupus La protein OS=Hor | 17.99 | 13.24 | 4 | 4 | 8.20 | 6.86 | 2 | 2 | 4.35 | 3.43 | 1 | 1 |
| P19338 | Nucleolin OS=Homo sapi | 44.44 | 13.24 | 7 | 9 | 41.70 | 11.27 | 6 | 8 | 36.85 | 10.85 | 4 | 7 |
| P35244 | Replication protein A 14 kDa subunit O | | 0.00 | | | | | | | 4.57 | 13.22 | 1 | 1 |
| Q9BWF3 | RNA-binding protein 4 OS | 4.89 | 4.40 | 1 | 1 | 4.32 | 4.40 | 1 | 1 | 5.32 | 8.79 | 1 | 1 |
| Q9NX55 | Huntingtin-interacting pro | 4.76 | 13.18 | 1 | 1 | 4.23 | 13.18 | 1 | 1 | | 0.00 | | |
| P06702 | Protein S100-A9 OS=Hom | 8.22 | 13.16 | 1 | 2 | | 0.00 | | | | 0.00 | | |
| Q9Y224 | UPF0568 protein C14orf1 | 3.84 | 6.56 | 1 | 1 | | 0.00 | | | 5.14 | 6.56 | 1 | 1 |
| Q8IY81 | pre-rRNA processing prot | 9.57 | 5.08 | 2 | 2 | 18.83 | 10.04 | 4 | 4 | 15.89 | 8.03 | 3 | 3 |
| Q6P1L8 | 39S ribosomal protein L1 | 4.94 | 13.10 | 1 | 1 | 5.20 | 13.10 | 1 | 1 | | 0.00 | | |
| Q8N5G0 | Small integral membrane protein 20 O | | 0.00 | | | 5.04 | 13.10 | 1 | 1 | | 0.00 | | |
| Q9NR45 | Sialic acid synthase OS=H | 8.84 | 5.01 | 1 | 2 | 4.21 | 3.90 | 1 | 1 | 4.69 | 4.18 | 1 | 1 |
| P54105 | Methylosome subunit pICln OS=Homo | | 0.00 | | | 5.79 | 13.08 | 1 | 1 | | 0.00 | | |
| P62854 | 40S ribosomal protein S2 | 3.92 | 13.04 | 1 | 1 | | 0.00 | | | | | | |
| P31150 | Rab GDP dissociation inhi | 28.89 | 12.98 | 4 | 5 | 27.41 | 12.98 | 4 | 5 | 25.61 | 12.98 | 4 | 5 |
| P62942 | Peptidyl-prolyl cis-trans is | 9.76 | 12.96 | 1 | 2 | | 0.00 | | | 4.25 | 12.96 | 1 | 1 |
| O43390 | Heterogeneous nuclear ri | 12.52 | 7.90 | 3 | 3 | 8.95 | 4.42 | 2 | 2 | 8.86 | 4.74 | 2 | 2 |
| P53396 | ATP-citrate synthase OS= | 32.14 | 10.17 | 7 | 7 | 34.01 | 8.81 | 6 | 7 | 18.84 | 5.81 | 4 | 4 |
| P28066 | Proteasome subunit alpha | 4.50 | 4.98 | 1 | 1 | 4.62 | 4.98 | 1 | 1 | 4.97 | 7.88 | 1 | 1 |
| Q9GZT3 | SRA stem-loop-interacting RNA-bindin | | 0.00 | | | 4.92 | 12.84 | 1 | 1 | | 0.00 | | |
| Q9Y2Q9 | 28S ribosomal protein S2 | 5.14 | 12.83 | 1 | 1 | 5.22 | 12.83 | 1 | 1 | | 0.00 | | |
| P43686 | 26S protease regulatory s | 4.78 | 4.78 | 1 | 1 | 5.77 | 7.89 | 1 | 1 | 11.87 | 7.89 | 1 | 2 |
| Q8N5N7 | 39S ribosomal protein L5 | 4.31 | 12.66 | 1 | 1 | 4.02 | 12.66 | 1 | 1 | | 0.00 | | |
| Q9H3Z4 | DnaJ homolog subfamily C member 5 | | 0.00 | | | 5.32 | 12.63 | 1 | 1 | | 0.00 | | |
| P62805 | Histone H4 OS=Homo sa | 9.54 | 12.62 | 2 | 2 | | 0.00 | | | 5.36 | 12.62 | 1 | 1 |
| O76070 | Gamma-synuclein OS=Ho | 4.67 | 12.60 | 1 | 1 | 3.95 | 12.60 | 1 | 1 | | | | |
| O43776 | Asparagine--tRNA ligase, | 17.79 | 9.31 | 3 | 4 | | 0.00 | | | 18.56 | 10.40 | 3 | 4 |
| O96005 | Cleft lip and palate transr | 4.93 | 3.29 | 1 | 1 | 5.34 | 3.29 | 1 | 1 | 10.98 | 9.27 | 2 | 2 |
| Q14247 | Src substrate cortactin OS | 16.79 | 12.55 | 3 | 3 | 9.46 | 6.73 | 2 | 2 | 19.53 | 6.73 | 2 | 4 |
| Q13158 | FAS-associated death domain protein | | 0.00 | | | 5.44 | 12.50 | 1 | 1 | | 0.00 | | |
| Q8TCD5 | 5'(3')-deoxyribonucleotidase, cytosolic | | 0.00 | | | 4.76 | 12.44 | 1 | 1 | 5.22 | 12.44 | 1 | 1 |

| | | | | | | | | | | | | | |
|---|---|---|---|---|---|---|---|---|---|---|---|---|---|
| Q9NX24 | H/ACA ribonucleoprotein | 5.50 | 12.42 | 1 | 1 | 9.29 | 12.42 | 1 | 2 | 5.44 | 12.42 | 1 | 1 |
| O75312 | Zinc finger protein ZPR1 OS=Homo sa | 0.00 | | | | 4.43 | 3.05 | 1 | 1 | 10.09 | 9.37 | 2 | 2 |
| O60739 | Eukaryotic translation init | 4.02 | 12.39 | 1 | 1 | | 0.00 | | | | 0.00 | | |
| Q96LD1 | Zeta-sarcoglycan OS=Hoı | 5.61 | 12.37 | 1 | 1 | | 0.00 | | | | 0.00 | | |
| P13639 | Elongation factor 2 OS=H | 30.37 | 8.62 | 4 | 6 | 29.80 | 9.09 | 4 | 6 | 50.82 | 11.31 | 6 | 10 |
| P68104 | Elongation factor 1-alpha | 11.82 | 7.14 | 2 | 2 | 9.99 | 12.12 | 2 | 2 | 6.12 | 6.93 | 1 | 1 |
| P04040 | Catalase OS=Homo sapiens GN=CAT | 0.00 | | | | 9.87 | 8.35 | 2 | 2 | 4.12 | 3.98 | 1 | 1 |
| P00387 | NADH-cytochrome b5 rec | 5.40 | 5.98 | 1 | 1 | 9.42 | 12.29 | 2 | 2 | 9.29 | 12.29 | 2 | 2 |
| P49368 | T-complex protein 1 subu | 13.90 | 10.09 | 3 | 3 | 9.04 | 5.87 | 2 | 2 | | 0.00 | | |
| Q9NRF9 | DNA polymerase epsilon subunit 3 OS | 0.00 | | | | | 0.00 | | | 4.70 | 12.24 | 1 | 1 |
| P50990 | T-complex protein 1 subu | 17.14 | 10.04 | 4 | 4 | 8.91 | 4.93 | 2 | 2 | 13.59 | 7.48 | 3 | 3 |
| P28482 | Mitogen-activated proteir | 9.34 | 12.22 | 2 | 2 | | 0.00 | | | 5.10 | 4.17 | 1 | 1 |
| Q13561 | Dynactin subunit 2 OS=H | 9.39 | 8.23 | 2 | 2 | 3.93 | 3.99 | 1 | 1 | 5.39 | 4.49 | 1 | 1 |
| Q14011 | Cold-inducible RNA-bindir | 4.81 | 12.21 | 1 | 1 | 4.07 | 12.21 | 1 | 1 | 5.26 | 12.21 | 1 | 1 |
| Q96FW1 | Ubiquitin thioesterase OTUB1 OS=Hor | 0.00 | | | | 9.64 | 5.54 | 1 | 2 | 12.80 | 12.18 | 2 | 3 |
| Q9UQ80 | Proliferation-associated p | 4.25 | 3.30 | 1 | 1 | 8.64 | 7.87 | 2 | 2 | 14.38 | 8.88 | 2 | 3 |
| Q9H4A4 | Aminopeptidase B OS=Hc | 8.76 | 4.62 | 2 | 2 | 9.06 | 4.77 | 2 | 2 | 4.43 | 2.77 | 1 | 1 |
| P20290 | Transcription factor BTF3 OS=Homo s | 0.00 | | | | | 0.00 | | | 4.60 | 12.14 | 1 | 1 |
| P06493 | Cyclin-dependent kinase | 4.83 | 6.73 | 1 | 1 | 4.25 | 5.39 | 1 | 1 | 9.02 | 12.12 | 2 | 2 |
| P48147 | Prolyl endopeptidase OS= | 18.73 | 9.86 | 4 | 4 | 4.92 | 2.39 | 1 | 1 | 15.04 | 7.75 | 3 | 3 |
| P04181 | Ornithine aminotransferas | 9.90 | 4.56 | 1 | 2 | 5.67 | 4.56 | 1 | 1 | 10.92 | 12.07 | 2 | 2 |
| Q96DA6 | Mitochondrial import inner membrane | 0.00 | | | | | 0.00 | | | 4.17 | 12.07 | 1 | 1 |
| P52597 | Heterogeneous nuclear ri | 15.29 | 12.05 | 3 | 3 | 15.33 | 12.05 | 3 | 3 | 15.92 | 7.95 | 2 | 3 |
| P04844 | Dolichyl-diphosphooligosa | 22.39 | 9.35 | 4 | 5 | 13.52 | 7.13 | 3 | 3 | 13.60 | 7.61 | 3 | 3 |
| P62330 | ADP-ribosylation factor 6 | 4.09 | 12.00 | 1 | 1 | | 0.00 | | | | 0.00 | | |
| Q99733 | Nucleosome assembly pro | 16.22 | 12.00 | 2 | 3 | | 0.00 | | | 10.64 | 12.00 | 2 | 2 |
| Q9NRX4 | 14 kDa phosphohistidine phosphatase | 0.00 | | | | 4.15 | 12.00 | 1 | 1 | | 0.00 | | |
| Q15717 | ELAV-like protein 1 OS=Homo sapiens | 0.00 | | | | 3.96 | 4.29 | 1 | 1 | 10.11 | 11.96 | 2 | 2 |
| Q14116 | Interleukin-18 OS=Homo sapiens GN= | 0.00 | | | | | 0.00 | | | 5.79 | 11.92 | 1 | 1 |
| P39023 | 60S ribosomal protein L3 | 5.12 | 5.96 | 1 | 1 | 10.82 | 6.45 | 2 | 2 | 15.30 | 11.91 | 3 | 3 |
| O60814 | Histone H2B type 1-K OS | 5.10 | 11.90 | 1 | 1 | 5.13 | 11.90 | 1 | 1 | 5.20 | 11.90 | 1 | 1 |
| P05026 | Sodium/potassium-transp | 10.00 | 8.25 | 1 | 2 | 9.73 | 11.88 | 2 | 2 | 16.64 | 8.25 | 1 | 3 |
| P49591 | Serine--tRNA ligase, cytoplasmic OS=I | 0.00 | | | | 4.77 | 5.45 | 1 | 1 | 9.35 | 6.42 | 2 | 2 |
| Q9UJU6 | Drebrin-like protein OS=H | 10.31 | 8.37 | 2 | 2 | 4.95 | 3.72 | 1 | 1 | 9.32 | 7.21 | 2 | 2 |
| P09382 | Galectin-1 OS=Homo sap | 4.34 | 11.85 | 1 | 1 | 4.09 | 11.85 | 1 | 1 | | 0.00 | | |
| P61289 | Proteasome activator con | 3.90 | 5.91 | 1 | 1 | 4.97 | 5.91 | 1 | 1 | 4.81 | 5.91 | 1 | 1 |
| P31040 | Succinate dehydrogenase | 15.07 | 11.75 | 3 | 3 | 14.42 | 11.75 | 3 | 3 | 11.02 | 6.63 | 2 | 2 |
| P16403 | Histone H1.2 OS=Homo s | 4.35 | 5.63 | 1 | 1 | 8.05 | 11.74 | 2 | 2 | 13.95 | 11.74 | 2 | 3 |
| Q96AG4 | Leucine-rich repeat-containing protein | 0.00 | | | | | 0.00 | | | 11.57 | 11.73 | 2 | 2 |

| | | | | | | | | | | | | | |
|---|---|---|---|---|---|---|---|---|---|---|---|---|---|
| Q9UI09 | NADH dehydrogenase [ul | 4.23 | 11.72 | 1 | 1 | | 0.00 | | | | 0.00 | | |
| Q15056 | Eukaryotic translation init | 5.63 | 11.69 | 1 | 1 | | 0.00 | | | | 0.00 | | |
| Q96QK1 | Vacuolar protein sorting-a | 16.67 | 5.65 | 3 | 4 | 18.44 | 5.65 | 3 | 4 | 15.29 | 6.16 | 3 | 3 |
| P17858 | ATP-dependent 6-phosph | 19.44 | 7.18 | 3 | 4 | 13.56 | 7.56 | 3 | 3 | 18.74 | 9.36 | 4 | 4 |
| Q92598 | Heat shock protein 105 k | 24.98 | 7.93 | 4 | 5 | 18.34 | 7.58 | 4 | 4 | 26.58 | 8.04 | 4 | 5 |
| P17980 | 26S protease regulatory s | 12.93 | 11.62 | 3 | 3 | 4.93 | 4.33 | 1 | 1 | 18.60 | 11.62 | 3 | 4 |
| Q9NR31 | GTP-binding protein SAR1 | 4.70 | 11.62 | 1 | 1 | | 0.00 | | | 4.30 | 11.62 | 1 | 1 |
| P23434 | Glycine cleavage system | 5.32 | 11.56 | 1 | 1 | 4.01 | 11.56 | 1 | 1 | 5.17 | 11.56 | 1 | 1 |
| Q12931 | Heat shock protein 75 kD | 13.70 | 9.66 | 3 | 3 | 12.72 | 3.84 | 2 | 3 | 9.76 | 3.84 | 2 | 2 |
| P30040 | Endoplasmic reticulum re | 12.68 | 11.49 | 2 | 3 | 4.27 | 5.75 | 1 | 1 | 4.64 | 5.75 | 1 | 1 |
| Q14376 | UDP-glucose 4-epimerase | 17.58 | 11.49 | 2 | 3 | 5.64 | 5.46 | 1 | 1 | 11.31 | 11.49 | 2 | 2 |
| Q7Z4W1 | L-xylulose reductase OS= | 5.90 | 11.48 | 1 | 1 | | 0.00 | | | 6.92 | 11.48 | 1 | 1 |
| O75937 | DnaJ homolog subfamily C member 8 | | 0.00 | | | | 0.00 | | | 5.53 | 11.46 | 1 | 1 |
| Q9NX63 | MICOS complex subunit M | 8.37 | 11.45 | 2 | 2 | | 0.00 | | | 4.39 | 6.61 | 1 | 1 |
| Q9H4M9 | EH domain-containing pro | 13.71 | 11.42 | 3 | 3 | 9.68 | 8.99 | 2 | 2 | 4.93 | 2.81 | 1 | 1 |
| Q9NQ55 | Suppressor of SWI4 1 ho | 5.10 | 7.82 | 1 | 1 | 4.29 | 3.59 | 1 | 1 | | 0.00 | | |
| P10109 | Adrenodoxin, mitochondrial OS=Homo | | 0.00 | | | | 0.00 | | | 4.42 | 11.41 | 1 | 1 |
| P21291 | Cysteine and glycine-rich | 4.54 | 11.40 | 1 | 1 | 4.59 | 11.40 | 1 | 1 | 4.64 | 11.40 | 1 | 1 |
| P24666 | Low molecular weight ph | 9.40 | 11.39 | 1 | 2 | 5.57 | 11.39 | 1 | 1 | 8.88 | 11.39 | 1 | 2 |
| O75489 | NADH dehydrogenase [ul | 3.93 | 4.92 | 1 | 1 | 4.39 | 6.44 | 1 | 1 | 4.48 | 4.92 | 1 | 1 |
| Q9NTK5 | Obg-like ATPase 1 OS=H | 12.94 | 11.36 | 3 | 3 | 4.24 | 3.79 | 1 | 1 | 4.23 | 3.79 | 1 | 1 |
| Q14166 | Tubulin--tyrosine ligase-li | 3.93 | 2.02 | 1 | 1 | | 0.00 | | | 15.11 | 9.32 | 3 | 3 |
| O75367 | Core histone macro-H2A. | 5.57 | 5.91 | 1 | 1 | 5.28 | 5.91 | 1 | 1 | 15.20 | 11.29 | 2 | 3 |
| Q96C86 | m7GpppX diphosphatase | 4.21 | 5.64 | 1 | 1 | 5.07 | 5.64 | 1 | 1 | 5.63 | 5.64 | 1 | 1 |
| P20839 | Inosine-5'-monophosphat | 11.19 | 5.06 | 1 | 2 | 4.20 | 2.33 | 1 | 1 | 12.29 | 8.95 | 2 | 2 |
| P62847 | 40S ribosomal protein S2 | 4.07 | 11.28 | 1 | 1 | | 0.00 | | | | 0.00 | | |
| Q15366 | Poly(rC)-binding protein 2 | 9.58 | 5.48 | 1 | 2 | 4.99 | 5.48 | 1 | 1 | 14.10 | 11.23 | 2 | 3 |
| Q5JTJ3 | Cytochrome c oxidase ass | 4.07 | 11.20 | 1 | 1 | | 0.00 | | | | 0.00 | | |
| P62899 | 60S ribosomal protein L31 OS=Homo | | 0.00 | | | 3.97 | 11.20 | 1 | 1 | | 0.00 | | |
| Q9UI30 | Multifunctional methyltransferase subu | | 0.00 | | | 4.54 | 11.20 | 1 | 1 | 5.29 | 11.20 | 1 | 1 |
| Q9NQP4 | Prefoldin subunit 4 OS=H | 4.17 | 11.19 | 1 | 1 | 4.41 | 11.19 | 1 | 1 | | 0.00 | | |
| P38606 | V-type proton ATPase cat | 18.46 | 7.78 | 3 | 4 | 10.44 | 6.32 | 2 | 2 | 12.21 | 6.32 | 2 | 2 |
| Q9Y6M9 | NADH dehydrogenase [ubiquinone] 1 | | 0.00 | | | | 0.00 | | | 4.13 | 11.17 | 1 | 1 |
| O14745 | Na(+)/H(+) exchange re | 16.63 | 7.26 | 1 | 3 | 13.98 | 11.17 | 2 | 3 | 10.36 | 7.26 | 1 | 2 |
| Q01518 | Adenylyl cyclase-associat | 13.89 | 11.16 | 3 | 3 | 5.06 | 3.79 | 1 | 1 | 4.34 | 3.79 | 1 | 1 |
| Q13501 | Sequestosome-1 OS=Homo sapiens G | | 0.00 | | | 5.29 | 3.41 | 1 | 1 | 11.39 | 11.14 | 2 | 2 |
| Q14157 | Ubiquitin-associated prote | 19.09 | 8.28 | 4 | 4 | 4.55 | 1.75 | 1 | 1 | 17.88 | 6.44 | 4 | 4 |
| O14602 | Eukaryotic translation init | 4.07 | 11.11 | 1 | 1 | | 0.00 | | | | 0.00 | | |
| P00491 | Purine nucleoside phosph | 7.95 | 11.07 | 2 | 2 | 4.54 | 5.88 | 1 | 1 | 4.20 | 5.88 | 1 | 1 |

| | | | | | | | | | | | | | |
|---|---|---|---|---|---|---|---|---|---|---|---|---|---|
| Q99832 | T-complex protein 1 subu | 21.60 | 11.05 | 3 | 4 | 16.59 | 8.47 | 2 | 3 | 16.74 | 6.63 | 2 | 3 |
| P36578 | 60S ribosomal protein L4 | 7.87 | 8.43 | 2 | 2 | 8.37 | 5.85 | 2 | 2 | 4.35 | 5.15 | 1 | 1 |
| Q7L5L3 | Glycerophosphodiester ph | 11.14 | 6.60 | 1 | 2 | 6.10 | 6.60 | 1 | 1 | 10.16 | 11.01 | 2 | 2 |
| P52209 | 6-phosphogluconate dehy | 9.00 | 8.07 | 2 | 2 | 10.36 | 7.04 | 2 | 2 | 6.13 | 3.93 | 1 | 1 |
| P0C0S5 | Histone H2A.Z OS=Homo sapiens GN= | | 0.00 | | | 4.08 | 10.94 | 1 | 1 | 4.19 | 10.94 | 1 | 1 |
| Q9Y5S9 | RNA-binding protein 8A OS=Homo sap | | 0.00 | | | | 0.00 | | | 4.13 | 10.92 | 1 | 1 |
| P62424 | 60S ribosomal protein L7a | 8.98 | 10.90 | 2 | 2 | 9.33 | 10.90 | 2 | 2 | | 0.00 | | |
| Q04760 | Lactoylglutathione lyase ( | 5.14 | 10.87 | 1 | 1 | 5.92 | 10.87 | 1 | 1 | 5.90 | 10.87 | 1 | 1 |
| Q9HCN8 | Stromal cell-derived factor 2-like prote | | 0.00 | | | | 0.00 | | | 5.26 | 10.86 | 1 | 1 |
| O15212 | Prefoldin subunit 6 OS=Homo sapiens | | 0.00 | | | | 0.00 | | | 4.77 | 10.85 | 1 | 1 |
| P18206 | Vinculin OS=Homo sapier | 27.52 | 8.47 | 5 | 5 | 20.43 | 6.17 | 4 | 4 | 25.41 | 6.79 | 4 | 5 |
| P50897 | Palmitoyl-protein thioeste | 8.40 | 10.78 | 2 | 2 | 4.92 | 5.88 | 1 | 1 | 5.07 | 5.88 | 1 | 1 |
| P61923 | Coatomer subunit zeta-1 OS=Homo sa | | 0.00 | | | | 0.00 | | | 4.72 | 10.73 | 1 | 1 |
| O75817 | Ribonuclease P protein su | 4.20 | 10.71 | 1 | 1 | | | | | | | | |
| P62829 | 60S ribosomal protein L2 | 4.13 | 10.71 | 1 | 1 | | 0.00 | | | 4.58 | 10.71 | 1 | 1 |
| O95433 | Activator of 90 kDa heat | 4.23 | 4.14 | 1 | 1 | | 0.00 | | | 4.82 | 6.51 | 1 | 1 |
| P61803 | Dolichyl-diphosphooligosa | 4.06 | 10.62 | 1 | 1 | | 0.00 | | | | | | |
| P15170 | Eukaryotic peptide chain | 8.57 | 5.61 | 2 | 2 | 8.94 | 7.41 | 2 | 2 | | 0.00 | | |
| Q13630 | GDP-L-fucose synthase O | 4.11 | 4.67 | 1 | 1 | 3.99 | 5.92 | 1 | 1 | | 0.00 | | |
| P20700 | Lamin-B1 OS=Homo sapi | 18.85 | 10.58 | 4 | 4 | 9.52 | 4.95 | 2 | 2 | 9.09 | 5.12 | 2 | 2 |
| Q14444 | Caprin-1 OS=Homo sapiens GN=CAPR | | 0.00 | | | | 0.00 | | | 13.43 | 10.58 | 3 | 3 |
| P00390 | Glutathione reductase, m | 9.40 | 10.54 | 2 | 2 | | 0.00 | | | 6.00 | 6.51 | 1 | 1 |
| P19105 | Myosin regulatory light ch | 4.97 | 10.53 | 1 | 1 | 5.07 | 10.53 | 1 | 1 | 5.28 | 10.53 | 1 | 1 |
| P12268 | Inosine-5'-monophosphat | 4.70 | 2.92 | 1 | 1 | | 0.00 | | | 13.55 | 10.51 | 3 | 3 |
| O00422 | Histone deacetylase comp | 4.39 | 10.46 | 1 | 1 | | 0.00 | | | 4.85 | 10.46 | 1 | 1 |
| Q9P2J5 | Leucine--tRNA ligase, cyt | 26.25 | 9.18 | 6 | 6 | 12.77 | 4.00 | 3 | 3 | 25.76 | 6.80 | 4 | 5 |
| P62888 | 60S ribosomal protein L3 | 4.28 | 10.43 | 1 | 1 | | 0.00 | | | | 0.00 | | |
| Q16851 | UTP--glucose-1-phosphat | 9.78 | 6.89 | 2 | 2 | 5.12 | 3.94 | 1 | 1 | 9.81 | 7.48 | 2 | 2 |
| Q07812 | Apoptosis regulator BAX ( | 4.71 | 10.42 | 1 | 1 | | 0.00 | | | 4.54 | 10.42 | 1 | 1 |
| Q02878 | 60S ribosomal protein L6 | 4.03 | 4.51 | 1 | 1 | | 0.00 | | | 4.49 | 5.90 | 1 | 1 |
| P62995 | Transformer-2 protein ho | 9.31 | 4.86 | 1 | 2 | | 0.00 | | | 5.23 | 5.56 | 1 | 1 |
| P36542 | ATP synthase subunit gar | 5.27 | 10.40 | 1 | 1 | | 0.00 | | | | 0.00 | | |
| Q9GZL7 | Ribosome biogenesis prot | 4.94 | 4.49 | 1 | 1 | | 0.00 | | | 5.16 | 5.91 | 1 | 1 |
| P05452 | Tetranectin OS=Homo sa | 4.84 | 10.40 | 1 | 1 | 4.56 | 10.40 | 1 | 1 | 4.16 | 10.40 | 1 | 1 |
| P35998 | 26S protease regulatory s | 4.67 | 4.39 | 1 | 1 | 8.68 | 6.00 | 2 | 2 | 9.55 | 4.39 | 1 | 2 |
| O14828 | Secretory carrier-associat | 9.71 | 4.61 | 1 | 2 | 10.09 | 4.61 | 1 | 2 | 19.37 | 10.37 | 2 | 4 |
| P17900 | Ganglioside GM2 activator OS=Homo | | 0.00 | | | 4.67 | 10.36 | 1 | 1 | | 0.00 | | |
| Q86X59 | Putative uncharacterized protein C17o | | 0.00 | | | 4.94 | 10.36 | 1 | 1 | | 0.00 | | |
| P39019 | 40S ribosomal protein S1 | 5.07 | 10.34 | 1 | 1 | | 0.00 | | | | 0.00 | | |

| | | | | | | | | | | | | | |
|---|---|---|---|---|---|---|---|---|---|---|---|---|---|
| Q9BRT2 | Ubiquinol-cytochrome-c reductase con | | 0.00 | | | | 0.00 | | | 4.54 | 10.32 | 1 | 1 |
| P00568 | Adenylate kinase isoenzyme 1 OS=Ho | | 0.00 | | | | 0.00 | | | 4.62 | 10.31 | 1 | 1 |
| Q99426 | Tubulin-folding cofactor B | 4.57 | 10.25 | 1 | 1 | | 0.00 | | | | 0.00 | | |
| Q7Z2W9 | 39S ribosomal protein L21, mitochond | | 0.00 | | | | 0.00 | | | 4.10 | 10.24 | 1 | 1 |
| P49748 | Very long-chain specific a | 14.32 | 10.23 | 3 | 3 | 3.94 | 3.66 | 1 | 1 | 4.46 | 3.66 | 1 | 1 |
| O75439 | Mitochondrial-processing | 10.59 | 10.22 | 2 | 2 | | 0.00 | | | 11.22 | 10.22 | 2 | 2 |
| O95169 | NADH dehydrogenase [ul | 4.48 | 10.22 | 1 | 1 | | 0.00 | | | 4.57 | 10.22 | 1 | 1 |
| Q6XQN6 | Nicotinate phosphoribosy | 9.57 | 7.62 | 2 | 2 | 4.52 | 2.60 | 1 | 1 | | 0.00 | | |
| Q15370 | Transcription elongation factor B polyp | | 0.00 | | | 4.16 | 10.17 | 1 | 1 | | 0.00 | | |
| P11766 | Alcohol dehydrogenase cl | 4.72 | 4.28 | 1 | 1 | 5.08 | 5.88 | 1 | 1 | 4.51 | 4.28 | 1 | 1 |
| P60900 | Proteasome subunit alpha | 3.85 | 4.88 | 1 | 1 | 4.15 | 5.28 | 1 | 1 | | 0.00 | | |
| P35268 | 60S ribosomal protein L22 OS=Homo | | 0.00 | | | 3.93 | 10.16 | 1 | 1 | | 0.00 | | |
| O60869 | Endothelial differentiation | 4.74 | 10.14 | 1 | 1 | 9.13 | 10.14 | 1 | 2 | | 0.00 | | |
| P61966 | AP-1 complex subunit sigma-1A OS=H | | 0.00 | | | 4.64 | 10.13 | 1 | 1 | 5.19 | 10.13 | 1 | 1 |
| P40763 | Signal transducer and act | 24.98 | 8.44 | 4 | 5 | 10.61 | 3.77 | 2 | 2 | 14.53 | 5.45 | 3 | 3 |
| Q00610 | Clathrin heavy chain 1 OS | 47.42 | 8.48 | 8 | 9 | 41.21 | 5.97 | 5 | 8 | 37.30 | 7.46 | 6 | 7 |
| O75436 | Vacuolar protein sorting-a | 8.70 | 10.09 | 2 | 2 | | 0.00 | | | | 0.00 | | |
| Q9Y508 | E3 ubiquitin-protein ligase | 9.37 | 10.09 | 1 | 2 | | 0.00 | | | 9.49 | 10.09 | 1 | 2 |
| O43684 | Mitotic checkpoint protein | 4.00 | 5.79 | 1 | 1 | 3.95 | 4.27 | 1 | 1 | 4.29 | 4.27 | 1 | 1 |
| O95747 | Serine/threonine-protein | 9.02 | 6.26 | 2 | 2 | 15.47 | 10.06 | 3 | 3 | 11.45 | 7.40 | 2 | 2 |
| Q03154 | Aminoacylase-1 OS=Hom | 4.46 | 4.41 | 1 | 1 | 4.70 | 5.64 | 1 | 1 | | 0.00 | | |
| P00367 | Glutamate dehydrogenas | 4.45 | 3.41 | 1 | 1 | 13.46 | 10.04 | 3 | 3 | 4.37 | 3.41 | 1 | 1 |
| P20810 | Calpastatin OS=Homo sa | 14.76 | 10.03 | 3 | 3 | 9.54 | 6.64 | 2 | 2 | 5.80 | 3.39 | 1 | 1 |
| Q6GMV3 | Putative peptidyl-tRNA hydrolase PTRH | | 0.00 | | | 3.95 | 10.00 | 1 | 1 | 4.36 | 10.00 | 1 | 1 |
| Q12849 | G-rich sequence factor 1 | 5.59 | 4.17 | 1 | 1 | 6.01 | 4.17 | 1 | 1 | 4.96 | 5.83 | 1 | 1 |
| O60506 | Heterogeneous nuclear ri | 9.88 | 5.14 | 2 | 2 | 9.82 | 3.37 | 2 | 2 | 25.75 | 8.99 | 3 | 5 |
| P26440 | Isovaleryl-CoA dehydroge | 4.30 | 4.02 | 1 | 1 | | 0.00 | | | 5.12 | 5.91 | 1 | 1 |
| P19013 | Keratin, type II cytoskele | 13.37 | 7.68 | 3 | 3 | 13.79 | 7.30 | 3 | 3 | 9.46 | 3.00 | 1 | 2 |
| P35249 | Replication factor C subu | 3.84 | 4.41 | 1 | 1 | | 0.00 | | | 6.03 | 5.51 | 1 | 1 |
| P11413 | Glucose-6-phosphate 1-d | 9.54 | 6.02 | 2 | 2 | 4.19 | 2.91 | 1 | 1 | 9.40 | 6.99 | 2 | 2 |
| P32969 | 60S ribosomal protein L9 OS=Homo s | | 0.00 | | | 5.48 | 9.90 | 1 | 1 | 5.24 | 9.90 | 1 | 1 |
| P61088 | Ubiquitin-conjugating enzyme E2 N OS | | 0.00 | | | 4.05 | 9.87 | 1 | 1 | | 0.00 | | |
| P00711 | Alpha-lactalbumin - Bos t | 5.20 | 9.86 | 1 | 1 | | 0.00 | | | 5.55 | 9.86 | 1 | 1 |
| P14314 | Glucosidase 2 subunit bet | 8.61 | 6.25 | 2 | 2 | 8.23 | 6.25 | 2 | 2 | 14.48 | 9.85 | 3 | 3 |
| Q96EY4 | Translation machinery-as | 9.13 | 9.85 | 1 | 2 | 4.36 | 9.85 | 1 | 1 | 4.19 | 9.85 | 1 | 1 |
| Q9BX68 | Histidine triad nucleotide- | 3.85 | 9.82 | 1 | 1 | | 0.00 | | | | 0.00 | | |
| P56537 | Eukaryotic translation init | 4.76 | 9.80 | 1 | 1 | | 0.00 | | | 4.26 | 9.80 | 1 | 1 |
| P46782 | 40S ribosomal protein S5 | 4.48 | 9.80 | 1 | 1 | 10.27 | 9.80 | 1 | 2 | 5.50 | 9.80 | 1 | 1 |
| Q96EK6 | Glucosamine 6-phosphate N-acetyltran | | 0.00 | | | | 0.00 | | | 4.57 | 9.78 | 1 | 1 |

| | | | | | | | | | | | | | |
|---|---|---|---|---|---|---|---|---|---|---|---|---|---|
| Q99471 | Prefoldin subunit 5 OS=H | 4.33 | 9.74 | 1 | 1 | 4.45 | 9.74 | 1 | 1 | 4.24 | 9.74 | 1 | 1 |
| P25685 | DnaJ homolog subfamily B member 1 | 0.00 | | | | 8.51 | 9.71 | 2 | 2 | | 0.00 | | |
| P14625 | Endoplasmin OS=Homo s | 22.42 | 7.35 | 4 | 5 | 19.76 | 8.34 | 4 | 4 | 24.36 | 8.34 | 4 | 5 |
| P11233 | Ras-related protein Ral-A | 5.83 | 9.71 | 1 | 1 | 4.95 | 9.71 | 1 | 1 | 5.25 | 9.71 | 1 | 1 |
| P00374 | Dihydrofolate reductase ( | 5.56 | 9.63 | 1 | 1 | | 0.00 | | | 5.47 | 9.63 | 1 | 1 |
| P23921 | Ribonucleoside-diphospha | 12.22 | 7.70 | 2 | 2 | 15.62 | 9.60 | 3 | 3 | 27.23 | 9.60 | 3 | 5 |
| P42704 | Leucine-rich PPR motif-co | 27.59 | 7.39 | 6 | 6 | 14.45 | 3.59 | 3 | 3 | 10.12 | 2.15 | 2 | 2 |
| Q15149 | Plectin OS=Homo sapiens | 59.02 | 4.53 | 12 | 12 | 55.35 | 4.21 | 11 | 11 | 87.35 | 6.30 | 16 | 18 |
| Q9NQG5 | Regulation of nuclear pre | 8.22 | 9.51 | 2 | 2 | 4.08 | 4.60 | 1 | 1 | 9.18 | 9.51 | 2 | 2 |
| O00762 | Ubiquitin-conjugating enz | 4.29 | 9.50 | 1 | 1 | 4.70 | 9.50 | 1 | 1 | | 0.00 | | |
| Q9Y490 | Talin-1 OS=Homo sapien | 49.59 | 6.14 | 8 | 10 | 31.26 | 4.88 | 6 | 6 | 48.40 | 6.45 | 8 | 9 |
| P02668 | Kappa-casein [Contains: ( | 5.30 | 9.47 | 1 | 1 | 5.56 | 9.47 | 1 | 1 | 5.06 | 9.47 | 1 | 1 |
| O43488 | Aflatoxin B1 aldehyde red | 4.89 | 4.18 | 1 | 1 | 7.97 | 9.47 | 2 | 2 | 9.06 | 9.47 | 2 | 2 |
| P53004 | Biliverdin reductase A OS | 8.21 | 9.46 | 2 | 2 | | 0.00 | | | | 0.00 | | |
| Q8WUM4 | Programmed cell death 6 | 18.87 | 6.80 | 4 | 4 | 25.52 | 5.99 | 3 | 5 | 13.83 | 6.34 | 3 | 3 |
| P47914 | 60S ribosomal protein L29 OS=Homo | 0.00 | | | | | 0.00 | | | 4.15 | 9.43 | 1 | 1 |
| Q92552 | 28S ribosomal protein S27, mitochond | 0.00 | | | | 9.60 | 9.42 | 2 | 2 | 4.43 | 3.62 | 1 | 1 |
| Q16576 | Histone-binding protein R | 9.50 | 9.41 | 2 | 2 | 9.96 | 9.41 | 2 | 2 | | 0.00 | | |
| P57735 | Ras-related protein Rab-25 OS=Homo | 0.00 | | | | | 0.00 | | | 4.91 | 9.39 | 1 | 1 |
| Q9ULC4 | Malignant T-cell-amplified sequence 1 | 0.00 | | | | | 0.00 | | | 4.23 | 9.39 | 1 | 1 |
| Q9Y3B7 | 39S ribosomal protein L1 | 4.15 | 9.38 | 1 | 1 | | 0.00 | | | 5.36 | 9.38 | 1 | 1 |
| O14980 | Exportin-1 OS=Homo sap | 14.08 | 4.20 | 3 | 3 | 9.01 | 2.71 | 2 | 2 | 16.15 | 5.70 | 3 | 3 |
| P30042 | ES1 protein homolog, mitochondrial O | 0.00 | | | | 4.72 | 9.33 | 1 | 1 | | 0.00 | | |
| O75348 | V-type proton ATPase sul | 3.98 | 9.32 | 1 | 1 | | 0.00 | | | | 0.00 | | |
| Q9UNM6 | 26S proteasome non-ATPase regulato | 0.00 | | | | | 0.00 | | | 9.14 | 9.31 | 2 | 2 |
| Q9H1Z9 | Tetraspanin-10 OS=Homo | 4.79 | 9.30 | 1 | 1 | | 0.00 | | | | 0.00 | | |
| Q12906 | Interleukin enhancer-binc | 4.98 | 2.01 | 1 | 1 | 10.25 | 5.48 | 2 | 2 | 5.92 | 3.80 | 1 | 1 |
| P07195 | L-lactate dehydrogenase | 5.13 | 4.49 | 1 | 1 | 4.03 | 4.79 | 1 | 1 | 9.73 | 9.28 | 2 | 2 |
| Q9UGI8 | Testin OS=Homo sapiens | 9.44 | 9.26 | 2 | 2 | | 0.00 | | | 5.27 | 5.23 | 1 | 1 |
| O43708 | Maleylacetoacetate isome | 4.04 | 9.26 | 1 | 1 | | 0.00 | | | | 0.00 | | |
| P55786 | Puromycin-sensitive amin | 5.11 | 2.94 | 1 | 1 | 13.41 | 6.31 | 3 | 3 | 4.33 | 1.85 | 1 | 1 |
| Q9HCC0 | Methylcrotonoyl-CoA carb | 12.33 | 9.24 | 3 | 3 | 8.19 | 6.04 | 2 | 2 | 4.24 | 3.20 | 1 | 1 |
| O60682 | Musculin OS=Homo sapie | 3.87 | 9.22 | 1 | 1 | | 0.00 | | | | 0.00 | | |
| Q8N163 | Cell cycle and apoptosis r | 16.45 | 9.21 | 3 | 3 | 11.41 | 5.96 | 2 | 2 | 5.11 | 3.58 | 1 | 1 |
| P31948 | Stress-induced-phosphop | 15.43 | 3.68 | 1 | 3 | 8.50 | 6.81 | 2 | 2 | 19.83 | 9.21 | 3 | 4 |
| O15258 | Protein RER1 OS=Homo sapiens GN= | 0.00 | | | | 4.22 | 9.18 | 1 | 1 | | 0.00 | | |
| P13798 | Acylamino-acid-releasing | 4.95 | 3.14 | 1 | 1 | 4.21 | 2.05 | 1 | 1 | 13.92 | 7.10 | 3 | 3 |
| Q9Y678 | Coatomer subunit gamma | 14.25 | 6.86 | 3 | 3 | 13.52 | 4.35 | 2 | 3 | 4.18 | 2.06 | 1 | 1 |
| Q13555 | Calcium/calmodulin-depe | 8.69 | 5.73 | 2 | 2 | 4.43 | 3.41 | 1 | 1 | 9.46 | 6.81 | 2 | 2 |

| | | | | | | | | | | | | | |
|---|---|---|---|---|---|---|---|---|---|---|---|---|---|
| Q12792 | Twinfilin-1 OS=Homo sap | 9.05 | 9.14 | 2 | 2 | 4.81 | 5.43 | 1 | 1 | 4.91 | 5.43 | 1 | 1 |
| Q92945 | Far upstream element-bir | 13.34 | 6.61 | 3 | 3 | 13.04 | 5.06 | 2 | 3 | 9.25 | 5.63 | 2 | 2 |
| O43583 | Density-regulated protein OS=Homo s | 0.00 | | | | 4.17 | 9.09 | 1 | 1 | | 0.00 | | |
| Q9UHV9 | Prefoldin subunit 2 OS=Homo sapiens | 0.00 | | | | 3.93 | 9.09 | 1 | 1 | 4.13 | 9.09 | 1 | 1 |
| P30050 | 60S ribosomal protein L12 OS=Homo | 0.00 | | | | | 0.00 | | | 9.55 | 9.09 | 1 | 2 |
| P46783 | 40S ribosomal protein S1 | 3.95 | 8.48 | 1 | 1 | | 0.00 | | | 5.04 | 9.09 | 1 | 1 |
| P00441 | Superoxide dismutase [Cu-Zn] OS=Hc | 0.00 | | | | 4.06 | 9.09 | 1 | 1 | 4.26 | 9.09 | 1 | 1 |
| P82979 | SAP domain-containing ri | 4.62 | 9.05 | 1 | 1 | | 0.00 | | | | 0.00 | | |
| P14651 | Homeobox protein Hox-B3 OS=Homo | 0.00 | | | | 4.78 | 9.05 | 1 | 1 | | 0.00 | | |
| Q9BY77 | Polymerase delta-interact | 4.08 | 4.51 | 1 | 1 | 5.12 | 4.51 | 1 | 1 | | 0.00 | | |
| O00410 | Importin-5 OS=Homo sap | 24.11 | 7.75 | 4 | 5 | 9.69 | 3.74 | 2 | 2 | 19.04 | 4.38 | 3 | 4 |
| Q7Z406 | Myosin-14 OS=Homo sap | 28.12 | 4.81 | 6 | 6 | 19.35 | 3.31 | 4 | 4 | 34.57 | 5.06 | 7 | 7 |
| Q5TFE4 | 5'-nucleotidase domain-co | 4.08 | 4.62 | 1 | 1 | 8.87 | 9.01 | 2 | 2 | 5.50 | 4.40 | 1 | 1 |
| O95782 | AP-2 complex subunit alp | 5.10 | 2.76 | 1 | 1 | 14.77 | 6.24 | 3 | 3 | 15.36 | 6.24 | 3 | 3 |
| Q13084 | 39S ribosomal protein L2 | 12.23 | 8.98 | 1 | 2 | 5.80 | 8.98 | 1 | 1 | 5.06 | 8.98 | 1 | 1 |
| Q9BZE1 | 39S ribosomal protein L37, mitochond | 0.00 | | | | 4.17 | 3.78 | 1 | 1 | 5.02 | 5.20 | 1 | 1 |
| P04632 | Calpain small subunit 1 O | 5.60 | 8.96 | 1 | 1 | | 0.00 | | | 10.27 | 8.96 | 1 | 2 |
| P51570 | Galactokinase OS=Homo | 4.92 | 4.08 | 1 | 1 | 11.03 | 8.93 | 2 | 2 | | 0.00 | | |
| Q86SX6 | Glutaredoxin-related protein 5, mitoch | 0.00 | | | | | 0.00 | | | 5.25 | 8.92 | 1 | 1 |
| P84095 | Rho-related GTP-binding | 5.63 | 8.90 | 1 | 1 | | 0.00 | | | 5.45 | 8.90 | 1 | 1 |
| O75934 | Pre-mRNA-splicing factor | 4.42 | 8.89 | 1 | 1 | | 0.00 | | | | 0.00 | | |
| Q13442 | 28 kDa heat- and acid-sta | 4.55 | 8.84 | 1 | 1 | 4.76 | 8.84 | 1 | 1 | | 0.00 | | |
| P07910 | Heterogeneous nuclear ri | 5.44 | 5.23 | 1 | 1 | 9.43 | 8.82 | 2 | 2 | 9.66 | 8.82 | 2 | 2 |
| Q9Y6A4 | Cilia- and flagella-associa | 4.03 | 8.81 | 1 | 1 | | 0.00 | | | | 0.00 | | |
| Q13907 | Isopentenyl-diphosphate | 3.86 | 8.81 | 1 | 1 | | 0.00 | | | | 0.00 | | |
| Q9Y2B0 | Protein canopy homolog 2 | 4.00 | 8.79 | 1 | 1 | 4.34 | 8.79 | 1 | 1 | 4.44 | 8.79 | 1 | 1 |
| P51398 | 28S ribosomal protein S29, mitochond | 0.00 | | | | | 0.00 | | | 10.96 | 8.79 | 2 | 2 |
| Q9BY32 | Inosine triphosphate pyro | 4.81 | 8.76 | 1 | 1 | 4.69 | 8.76 | 1 | 1 | 8.38 | 8.76 | 1 | 2 |
| P34949 | Mannose-6-phosphate iso | 4.08 | 4.96 | 1 | 1 | 4.09 | 3.78 | 1 | 1 | 4.21 | 3.78 | 1 | 1 |
| P18621 | 60S ribosomal protein L1 | 5.14 | 8.70 | 1 | 1 | 4.40 | 8.70 | 1 | 1 | 10.46 | 8.70 | 1 | 2 |
| O60936 | Nucleolar protein 3 OS=Homo sapiens | 0.00 | | | | 4.73 | 8.68 | 1 | 1 | | 0.00 | | |
| P04083 | Annexin A1 OS=Homo sapiens GN=AN | 0.00 | | | | | 0.00 | | | 8.42 | 8.67 | 2 | 2 |
| P84098 | 60S ribosomal protein L1 | 5.66 | 8.67 | 1 | 1 | 5.12 | 8.67 | 1 | 1 | 4.73 | 8.67 | 1 | 1 |
| P12277 | Creatine kinase B-type OS | 10.54 | 8.66 | 2 | 2 | 4.55 | 4.20 | 1 | 1 | 4.49 | 4.20 | 1 | 1 |
| Q15404 | Ras suppressor protein 1 | 5.41 | 8.66 | 1 | 1 | 7.16 | 8.66 | 1 | 1 | 5.96 | 8.66 | 1 | 1 |
| Q92499 | ATP-dependent RNA helic | 10.13 | 5.27 | 2 | 2 | 17.88 | 8.65 | 4 | 4 | 9.44 | 5.27 | 2 | 2 |
| Q8NBT2 | Kinetochore protein Spc24 OS=Homo | 0.00 | | | | 4.11 | 8.63 | 1 | 1 | 5.25 | 8.63 | 1 | 1 |
| Q9UEU0 | Vesicle transport through | 4.55 | 8.62 | 1 | 1 | | 0.00 | | | | 0.00 | | |
| O15498 | Synaptobrevin homolog Y | 4.06 | 8.59 | 1 | 1 | 4.14 | 8.59 | 1 | 1 | | 0.00 | | |

| | | | | | | | | | | | | | |
|---|---|---|---|---|---|---|---|---|---|---|---|---|---|
| Q14974 | Importin subunit beta-1 ( | 24.76 | 8.56 | 5 | 5 | 12.95 | 4.91 | 3 | 3 | 11.23 | 3.88 | 2 | 2 |
| Q96HC4 | PDZ and LIM domain pro | 14.43 | 8.56 | 2 | 3 | 5.51 | 5.37 | 1 | 1 | | 0.00 | | |
| Q9BY43 | Charged multivesicular body protein 4 | 0.00 | | | | | 0.00 | | | 4.22 | 8.56 | 1 | 1 |
| P84103 | Serine/arginine-rich splici | 3.99 | 8.54 | 1 | 1 | | 0.00 | | | | 0.00 | | |
| P38919 | Eukaryotic initiation facto | 10.43 | 8.52 | 2 | 2 | 5.35 | 3.89 | 1 | 1 | 9.79 | 8.52 | 2 | 2 |
| P63267 | Actin, gamma-enteric sm | 13.87 | 8.51 | 2 | 3 | 13.95 | 8.51 | 2 | 3 | 18.72 | 8.51 | 2 | 4 |
| O00151 | PDZ and LIM domain protein 1 OS=Hc | 0.00 | | | | 4.99 | 8.51 | 1 | 1 | | 0.00 | | |
| P53618 | Coatomer subunit beta O | 19.46 | 6.61 | 3 | 4 | 9.82 | 3.78 | 2 | 2 | 5.29 | 1.89 | 1 | 1 |
| P54578 | Ubiquitin carboxyl-termin | 5.74 | 4.86 | 1 | 1 | 14.05 | 8.50 | 3 | 3 | 6.21 | 4.86 | 1 | 1 |
| Q8WVQ1 | Soluble calcium-activated | 9.08 | 8.48 | 2 | 2 | 5.23 | 4.49 | 1 | 1 | 5.73 | 4.49 | 1 | 1 |
| Q9BQA1 | Methylosome protein 50 OS=Homo sa | 0.00 | | | | 4.45 | 4.09 | 1 | 1 | 9.59 | 8.48 | 2 | 2 |
| P60033 | CD81 antigen OS=Homo | 6.16 | 8.47 | 1 | 1 | | 0.00 | | | | 0.00 | | |
| Q14749 | Glycine N-methyltransfera | 4.87 | 8.47 | 1 | 1 | | 0.00 | | | | 0.00 | | |
| Q9HD15 | Steroid receptor RNA acti | 4.02 | 8.47 | 1 | 1 | | 0.00 | | | | 0.00 | | |
| P52566 | Rho GDP-dissociation inhibitor 2 OS=H | 0.00 | | | | | 0.00 | | | 5.10 | 8.46 | 1 | 1 |
| Q9UL25 | Ras-related protein Rab-21 OS=Homo | 0.00 | | | | | 0.00 | | | 4.27 | 8.44 | 1 | 1 |
| A6NKF9 | Putative Golgi pH regulator C OS=Hom | 0.00 | | | | | 0.00 | | | 5.39 | 8.44 | 1 | 1 |
| O00148 | ATP-dependent RNA helic | 4.44 | 3.28 | 1 | 1 | 4.35 | 3.28 | 1 | 1 | 5.06 | 5.15 | 1 | 1 |
| Q9UHY7 | Enolase-phosphatase E1 OS=Homo sa | 0.00 | | | | | 0.00 | | | 5.43 | 8.43 | 1 | 1 |
| P11388 | DNA topoisomerase 2-alp | 29.26 | 4.51 | 5 | 6 | 23.00 | 4.25 | 5 | 5 | 31.46 | 6.60 | 5 | 6 |
| Q9Y3C1 | Nucleolar protein 16 OS=Homo sapier | 0.00 | | | | 4.06 | 8.43 | 1 | 1 | | 0.00 | | |
| P78347 | General transcription fact | 13.67 | 3.91 | 2 | 3 | 14.52 | 4.21 | 2 | 3 | 9.91 | 3.71 | 2 | 2 |
| P13861 | cAMP-dependent protein | 15.78 | 8.42 | 2 | 3 | | 0.00 | | | 5.39 | 4.21 | 1 | 1 |
| P22087 | rRNA 2'-O-methyltransfer | 3.86 | 8.41 | 1 | 1 | | 0.00 | | | | 0.00 | | |
| Q96E11 | Ribosome-recycling facto | 5.38 | 8.40 | 1 | 1 | | 0.00 | | | | 0.00 | | |
| O00487 | 26S proteasome non-ATPase regulato | 0.00 | | | | 5.00 | 8.39 | 1 | 1 | | 0.00 | | |
| P62195 | 26S protease regulatory s | 10.02 | 8.37 | 2 | 2 | | 0.00 | | | 10.67 | 8.37 | 2 | 2 |
| P22061 | Protein-L-isoaspartate(D- | 4.28 | 8.37 | 1 | 1 | | 0.00 | | | | 0.00 | | |
| Q00839 | Heterogeneous nuclear ri | 11.29 | 5.70 | 2 | 2 | 15.46 | 8.36 | 3 | 3 | 11.62 | 5.70 | 2 | 2 |
| P30049 | ATP synthase subunit delta, mitochon | 0.00 | | | | 4.22 | 8.33 | 1 | 1 | 4.24 | 8.33 | 1 | 1 |
| P06133 | UDP-glucuronosyltransfer | 5.58 | 8.33 | 1 | 1 | | 0.00 | | | | 0.00 | | |
| P40121 | Macrophage-capping prot | 8.76 | 8.33 | 2 | 2 | 4.01 | 4.60 | 1 | 1 | 4.17 | 4.60 | 1 | 1 |
| P60228 | Eukaryotic translation init | 19.05 | 8.31 | 2 | 4 | 15.16 | 8.31 | 2 | 3 | 15.65 | 8.31 | 2 | 3 |
| Q96S44 | TP53-regulating kinase OS=Homo sap | 0.00 | | | | | 0.00 | | | 10.56 | 8.30 | 1 | 2 |
| Q8IYU8 | Calcium uptake protein 2, | 7.98 | 8.29 | 2 | 2 | 4.03 | 3.92 | 1 | 1 | | 0.00 | | |
| P83731 | 60S ribosomal protein L2 | 4.30 | 8.28 | 1 | 1 | 8.59 | 8.28 | 1 | 2 | 4.48 | 8.28 | 1 | 1 |
| Q9NXV6 | CDKN2A-interacting protein OS=Homo | 0.00 | | | | 5.56 | 3.62 | 1 | 1 | 10.40 | 8.28 | 2 | 2 |
| Q5T2N8 | ATPase family AAA doma | 5.31 | 4.87 | 1 | 1 | | 0.00 | | | 4.59 | 3.41 | 1 | 1 |
| P54725 | UV excision repair proteir | 6.14 | 8.26 | 1 | 1 | 5.04 | 8.26 | 1 | 1 | | 0.00 | | |

| | | | | | | | | | | | | | |
|---|---|---|---|---|---|---|---|---|---|---|---|---|---|
| Q02818 | Nucleobindin-1 OS=Homo | 4.85 | 5.42 | 1 | 1 | 4.70 | 2.82 | 1 | 1 | | 0.00 | | |
| Q9H2W6 | 39S ribosomal protein L4( | 4.91 | 8.24 | 1 | 1 | | 0.00 | | | | 0.00 | | |
| P17174 | Aspartate aminotransfera | 9.03 | 8.23 | 2 | 2 | | 0.00 | | | 9.28 | 8.23 | 2 | 2 |
| O95757 | Heat shock 70 kDa protei | 14.24 | 4.17 | 2 | 3 | 18.90 | 5.96 | 3 | 4 | | 0.00 | | |
| Q8TF63 | Dendritic cell nuclear protein 1 OS=Hc | | 0.00 | | | | 0.00 | | | 4.29 | 8.20 | 1 | 1 |
| Q15287 | RNA-binding protein with | 5.61 | 4.92 | 1 | 1 | 9.55 | 8.20 | 2 | 2 | 4.68 | 4.92 | 1 | 1 |
| P23246 | Splicing factor, proline- a | 13.67 | 8.20 | 3 | 3 | 4.46 | 3.25 | 1 | 1 | 14.14 | 8.20 | 3 | 3 |
| Q16643 | Drebrin OS=Homo sapier | 5.41 | 2.77 | 1 | 1 | 4.31 | 3.08 | 1 | 1 | 5.15 | 2.31 | 1 | 1 |
| P10768 | S-formylglutathione hydro | 6.69 | 8.16 | 1 | 1 | 5.28 | 8.16 | 1 | 1 | 4.29 | 8.16 | 1 | 1 |
| Q9BT78 | COP9 signalosome compl | 5.43 | 4.19 | 1 | 1 | 9.20 | 8.13 | 2 | 2 | 4.85 | 4.19 | 1 | 1 |
| Q16401 | 26S proteasome non-ATP | 10.65 | 8.13 | 2 | 2 | 10.45 | 8.13 | 2 | 2 | 5.42 | 4.37 | 1 | 1 |
| Q92804 | TATA-binding protein-ass | 9.33 | 8.11 | 2 | 2 | 4.99 | 5.74 | 1 | 1 | | 0.00 | | |
| Q15257 | Serine/threonine-protein | 4.85 | 8.10 | 1 | 1 | | 0.00 | | | | 0.00 | | |
| P19388 | DNA-directed RNA polyme | 4.36 | 8.10 | 1 | 1 | | 0.00 | | | 4.61 | 8.10 | 1 | 1 |
| Q96I99 | Succinyl-CoA ligase [GDP | 4.84 | 4.63 | 1 | 1 | | 0.00 | | | 9.72 | 8.10 | 2 | 2 |
| P07477 | Trypsin-1 OS=Homo sapi | 4.07 | 8.10 | 1 | 1 | 9.69 | 8.10 | 1 | 2 | 5.37 | 8.10 | 1 | 1 |
| O15173 | Membrane-associated progesterone re | | 0.00 | | | | 0.00 | | | 5.18 | 8.07 | 1 | 1 |
| O00231 | 26S proteasome non-ATP | 4.22 | 3.32 | 1 | 1 | 3.92 | 4.74 | 1 | 1 | | 0.00 | | |
| Q9P2T1 | GMP reductase 2 OS=Ho | 5.09 | 8.05 | 1 | 1 | | 0.00 | | | 5.20 | 8.05 | 1 | 1 |
| Q9Y263 | Phospholipase A-2-activa | 8.71 | 5.53 | 2 | 2 | 4.26 | 2.39 | 1 | 1 | 5.13 | 2.52 | 1 | 1 |
| Q06830 | Peroxiredoxin-1 OS=Homo sapiens GN | | 0.00 | | | | 0.00 | | | 4.35 | 8.04 | 1 | 1 |
| P11586 | C-1-tetrahydrofolate synt | 18.33 | 6.31 | 4 | 4 | 14.37 | 5.13 | 3 | 3 | 14.57 | 5.13 | 3 | 3 |
| P33991 | DNA replication licensing factor MCM4 | | 0.00 | | | 4.10 | 2.20 | 1 | 1 | 15.17 | 8.00 | 3 | 3 |
| P09874 | Poly [ADP-ribose] polyme | 4.96 | 3.06 | 1 | 1 | 10.10 | 3.16 | 2 | 2 | 17.65 | 7.89 | 4 | 4 |
| P48047 | ATP synthase subunit O, | 5.17 | 7.98 | 1 | 1 | 9.92 | 7.98 | 1 | 2 | | 0.00 | | |
| P13796 | Plastin-2 OS=Homo sapie | 3.85 | 2.55 | 1 | 1 | 9.16 | 5.58 | 2 | 2 | 4.15 | 2.39 | 1 | 1 |
| Q15005 | Signal peptidase complex | 3.91 | 7.96 | 1 | 1 | | 0.00 | | | 4.47 | 7.96 | 1 | 1 |
| Q15363 | Transmembrane emp24 c | 4.94 | 7.96 | 1 | 1 | | 0.00 | | | | 0.00 | | |
| P62191 | 26S protease regulatory s | 15.78 | 7.95 | 2 | 3 | 5.33 | 4.55 | 1 | 1 | 4.99 | 3.41 | 1 | 1 |
| P19623 | Spermidine synthase OS= | 10.11 | 7.95 | 1 | 2 | 4.28 | 7.95 | 1 | 1 | 4.70 | 7.95 | 1 | 1 |
| Q14697 | Neutral alpha-glucosidase | 8.74 | 4.66 | 2 | 2 | | 0.00 | | | 17.55 | 5.08 | 3 | 4 |
| Q15029 | 116 kDa U5 small nuclea | 24.50 | 6.07 | 3 | 5 | 5.18 | 1.75 | 1 | 1 | 19.92 | 7.92 | 4 | 4 |
| Q9Y383 | Putative RNA-binding pro | 12.78 | 7.91 | 2 | 3 | 4.38 | 3.83 | 1 | 1 | | 0.00 | | |
| P51553 | Isocitrate dehydrogenase | 6.27 | 7.89 | 1 | 1 | 6.95 | 7.89 | 1 | 1 | 6.70 | 7.89 | 1 | 1 |
| Q8N6H7 | ADP-ribosylation factor G | 5.89 | 3.45 | 1 | 1 | 9.43 | 3.45 | 1 | 2 | 4.95 | 4.41 | 1 | 1 |
| O95232 | Luc7-like protein 3 OS=H | 8.95 | 7.87 | 2 | 2 | 4.44 | 4.17 | 1 | 1 | 4.56 | 4.17 | 1 | 1 |
| P62913 | 60S ribosomal protein L1 | 3.89 | 7.87 | 1 | 1 | | 0.00 | | | | 0.00 | | |
| P61221 | ATP-binding cassette sub | 8.48 | 5.34 | 2 | 2 | 4.00 | 2.50 | 1 | 1 | 4.99 | 2.67 | 1 | 1 |
| Q8NC51 | Plasminogen activator inh | 12.45 | 7.84 | 2 | 3 | 5.00 | 3.92 | 1 | 1 | 9.98 | 7.84 | 2 | 2 |

| | | | | | | | | | | | | | |
|---|---|---|---|---|---|---|---|---|---|---|---|---|---|
| A6NCN2 | Putative keratin-87 protei | 5.81 | 7.84 | 1 | 1 | | 0.00 | | | 5.36 | 7.84 | 1 | 1 |
| O60934 | Nibrin OS=Homo sapiens | 4.74 | 2.25 | 1 | 1 | 4.79 | 2.25 | 1 | 1 | 5.16 | 3.32 | 1 | 1 |
| Q9H1E3 | Nuclear ubiquitous casein and cyclin-d | | 0.00 | | | 5.90 | 7.82 | 1 | 1 | 5.71 | 7.82 | 1 | 1 |
| Q13257 | Mitotic spindle assembly checkpoint pr | | 0.00 | | | 4.08 | 7.80 | 1 | 1 | | 0.00 | | |
| P49720 | Proteasome subunit beta type-3 OS=H | | 0.00 | | | 4.27 | 7.80 | 1 | 1 | 4.14 | 7.80 | 1 | 1 |
| Q9Y446 | Plakophilin-3 OS=Homo s | 13.77 | 5.90 | 3 | 3 | 8.89 | 3.89 | 2 | 2 | 18.43 | 7.78 | 4 | 4 |
| Q12874 | Splicing factor 3A subunit | 9.03 | 4.19 | 1 | 2 | 9.07 | 7.78 | 2 | 2 | 11.44 | 4.19 | 1 | 2 |
| P02786 | Transferrin receptor prote | 12.35 | 5.66 | 3 | 3 | 9.19 | 3.95 | 2 | 2 | | 0.00 | | |
| O75822 | Eukaryotic translation init | 4.39 | 7.75 | 1 | 1 | | 0.00 | | | 4.96 | 7.75 | 1 | 1 |
| P19367 | Hexokinase-1 OS=Homo | 14.05 | 5.23 | 3 | 3 | 17.69 | 6.43 | 4 | 4 | 15.30 | 3.93 | 2 | 3 |
| P07814 | Bifunctional glutamate/pr | 19.94 | 5.29 | 4 | 4 | 16.52 | 4.17 | 3 | 3 | 14.76 | 4.76 | 3 | 3 |
| Q9NY33 | Dipeptidyl peptidase 3 OS | 5.63 | 3.26 | 1 | 1 | 5.17 | 3.26 | 1 | 1 | 9.72 | 4.48 | 2 | 2 |
| P36957 | Dihydrolipoyllysine-residu | 4.51 | 3.09 | 1 | 1 | 11.77 | 7.73 | 2 | 2 | 6.61 | 4.64 | 1 | 1 |
| Q09666 | Neuroblast differentiation | 77.83 | 4.74 | 15 | 16 | 40.44 | 2.70 | 7 | 9 | 75.07 | 5.74 | 15 | 16 |
| Q9HC07 | Transmembrane protein | 5.26 | 7.72 | 1 | 1 | | 0.00 | | | | 0.00 | | |
| Q70J99 | Protein unc-13 homolog | 13.62 | 4.68 | 3 | 3 | 13.46 | 5.14 | 3 | 3 | 13.74 | 4.68 | 3 | 3 |
| Q8NE71 | ATP-binding cassette sub | 12.60 | 3.43 | 2 | 3 | 13.03 | 5.68 | 3 | 3 | 18.06 | 5.92 | 3 | 4 |
| Q96S94 | Cyclin-L2 OS=Homo sapiens GN=CCN | | 0.00 | | | | 0.00 | | | 4.99 | 7.69 | 1 | 1 |
| Q9GZZ1 | N-alpha-acetyltransferase 50 OS=Hom | | 0.00 | | | | 0.00 | | | 4.55 | 7.69 | 1 | 1 |
| Q08J23 | tRNA (cytosine(34)-C(5)) | 8.21 | 4.43 | 2 | 2 | 4.90 | 3.26 | 1 | 1 | 5.12 | 2.35 | 1 | 1 |
| Q01130 | Serine/arginine-rich splici | 4.93 | 7.69 | 1 | 1 | 9.62 | 7.69 | 1 | 2 | 5.40 | 7.69 | 1 | 1 |
| P31947 | 14-3-3 protein sigma OS=Homo sapie | | 0.00 | | | | 0.00 | | | 11.32 | 7.66 | 1 | 2 |
| Q8TEA8 | D-tyrosyl-tRNA(Tyr) deac | 5.08 | 7.66 | 1 | 1 | 4.90 | 7.66 | 1 | 1 | 9.48 | 7.66 | 1 | 2 |
| Q9NQT5 | Exosome complex compo | 5.96 | 7.64 | 1 | 1 | 4.12 | 7.64 | 1 | 1 | | 0.00 | | |
| P19404 | NADH dehydrogenase [ul | 4.27 | 7.63 | 1 | 1 | | 0.00 | | | | 0.00 | | |
| P55795 | Heterogeneous nuclear ri | 13.36 | 3.79 | 1 | 3 | | 0.00 | | | 10.03 | 7.57 | 2 | 2 |
| Q32MZ4 | Leucine-rich repeat flightl | 14.41 | 7.55 | 3 | 3 | 4.67 | 1.73 | 1 | 1 | 4.38 | 1.73 | 1 | 1 |
| Q14498 | RNA-binding protein 39 C | 5.54 | 3.21 | 1 | 1 | 4.68 | 3.21 | 1 | 1 | 10.54 | 7.55 | 2 | 2 |
| P09234 | U1 small nuclear ribonucl | 3.88 | 7.55 | 1 | 1 | | 0.00 | | | | 0.00 | | |
| Q01813 | ATP-dependent 6-phosph | 11.46 | 5.10 | 2 | 2 | 15.19 | 7.53 | 3 | 3 | 9.51 | 5.10 | 2 | 2 |
| Q13409 | Cytoplasmic dynein 1 inte | 4.76 | 4.23 | 1 | 1 | | 0.00 | | | 4.29 | 3.29 | 1 | 1 |
| P15311 | Ezrin OS=Homo sapiens ( | 13.36 | 7.51 | 3 | 3 | 5.53 | 2.56 | 1 | 1 | 5.80 | 2.56 | 1 | 1 |
| O43809 | Cleavage and polyadenyla | 4.47 | 7.49 | 1 | 1 | | 0.00 | | | | 0.00 | | |
| O95861 | 3'(2'),5'-bisphosphate nu | 4.86 | 7.47 | 1 | 1 | 10.24 | 7.47 | 1 | 2 | | 0.00 | | |
| Q6ZRV2 | Protein FAM83H OS=Hon | 13.59 | 4.58 | 3 | 3 | 4.17 | 1.53 | 1 | 1 | 10.05 | 2.88 | 2 | 2 |
| P27824 | Calnexin OS=Homo sapie | 4.05 | 2.70 | 1 | 1 | 4.00 | 2.70 | 1 | 1 | 9.42 | 4.73 | 2 | 2 |
| Q86UE4 | Protein LYRIC OS=Homo sapiens GN= | | 0.00 | | | | 0.00 | | | 9.89 | 7.39 | 2 | 2 |
| Q8WVX9 | Fatty acyl-CoA reductase | 16.62 | 7.38 | 3 | 4 | | 0.00 | | | | 0.00 | | |
| O60664 | Perilipin-3 OS=Homo sapiens GN=PLI | | 0.00 | | | 9.93 | 7.37 | 2 | 2 | 9.77 | 7.37 | 2 | 2 |

| | | | | | | | | | | | | | |
|---|---|---|---|---|---|---|---|---|---|---|---|---|---|
| P11310 | Medium-chain specific ac | 4.44 | 3.09 | 1 | 1 | 4.16 | 4.28 | 1 | 1 | 4.31 | 3.09 | 1 | 1 |
| Q15102 | Platelet-activating factor acetylhydrola | | 0.00 | | | 4.03 | 7.36 | 1 | 1 | | 0.00 | | |
| P63208 | S-phase kinase-associated protein 1 O | | 0.00 | | | 4.23 | 7.36 | 1 | 1 | | 0.00 | | |
| Q9Y294 | Histone chaperone ASF1A | 4.47 | 7.35 | 1 | 1 | | | | | 4.90 | 7.35 | 1 | 1 |
| O00287 | Regulatory factor X-associated protein | | 0.00 | | | 4.46 | 7.35 | 1 | 1 | | 0.00 | | |
| Q96S19 | UPF0585 protein C16orf13 OS=Homo | | 0.00 | | | | 0.00 | | | 4.12 | 7.35 | 1 | 1 |
| Q9UBQ5 | Eukaryotic translation initiation factor | | 0.00 | | | 5.71 | 7.34 | 1 | 1 | 5.08 | 7.34 | 1 | 1 |
| Q86VQ1 | Glucocorticoid-induced tra | 4.86 | 7.31 | 1 | 1 | | 0.00 | | | | 0.00 | | |
| O15145 | Actin-related protein 2/3 | 4.87 | 7.30 | 1 | 1 | 3.96 | 7.30 | 1 | 1 | | 0.00 | | |
| P23786 | Carnitine O-palmitoyltransferase 2, mi | | 0.00 | | | 4.78 | 3.34 | 1 | 1 | 10.46 | 7.29 | 2 | 2 |
| P22234 | Multifunctional protein AD | 3.81 | 3.06 | 1 | 1 | 3.93 | 3.06 | 1 | 1 | 4.78 | 4.24 | 1 | 1 |
| P30043 | Flavin reductase (NADPH | 4.17 | 7.28 | 1 | 1 | 4.09 | 7.28 | 1 | 1 | 5.16 | 7.28 | 1 | 1 |
| P30519 | Heme oxygenase 2 OS=Homo sapiens | | 0.00 | | | | 0.00 | | | 4.31 | 7.28 | 1 | 1 |
| P25789 | Proteasome subunit alpha type-4 OS= | | 0.00 | | | 4.04 | 7.28 | 1 | 1 | | 0.00 | | |
| Q96MX6 | WD repeat-containing protein 92 OS= | | 0.00 | | | | 0.00 | | | 4.80 | 7.28 | 1 | 1 |
| O75083 | WD repeat-containing pro | 5.33 | 2.64 | 1 | 1 | 5.25 | 2.64 | 1 | 1 | 10.49 | 7.26 | 2 | 2 |
| Q96A33 | Coiled-coil domain-contai | 9.02 | 7.25 | 2 | 2 | | 0.00 | | | 4.99 | 3.52 | 1 | 1 |
| P30084 | Enoyl-CoA hydratase, mit | 5.03 | 7.24 | 1 | 1 | 5.75 | 7.24 | 1 | 1 | 5.14 | 7.24 | 1 | 1 |
| P11216 | Glycogen phosphorylase, | 16.23 | 7.24 | 3 | 3 | 5.17 | 1.90 | 1 | 1 | 9.63 | 4.27 | 2 | 2 |
| O75431 | Metaxin-2 OS=Homo sapiens GN=MTX2 PE=1 SV=1 - [MTX2_HUMAN] | | | | | | 0.00 | | | 4.42 | 7.22 | 1 | 1 |
| Q99848 | Probable rRNA-processing protein EBP | | 0.00 | | | | 0.00 | | | 5.09 | 7.19 | 1 | 1 |
| P49407 | Beta-arrestin-1 OS=Homo sapiens GN | | 0.00 | | | | 0.00 | | | 5.76 | 7.18 | 1 | 1 |
| Q9UGT4 | Sushi domain-containing | 14.38 | 4.14 | 2 | 3 | 9.77 | 4.74 | 2 | 2 | 19.69 | 7.18 | 3 | 4 |
| Q9BQ67 | Glutamate-rich WD repea | 4.34 | 3.59 | 1 | 1 | | 0.00 | | | 9.75 | 7.17 | 2 | 2 |
| O00159 | Unconventional myosin-Ic | 24.27 | 7.15 | 4 | 5 | 9.83 | 3.48 | 2 | 2 | 8.55 | 1.79 | 1 | 2 |
| Q9Y2C2 | Uronyl 2-sulfotransferase | 4.73 | 7.14 | 1 | 1 | | 0.00 | | | | 0.00 | | |
| P13667 | Protein disulfide-isomeras | 19.06 | 7.13 | 3 | 4 | 14.72 | 7.13 | 3 | 3 | 4.58 | 2.33 | 1 | 1 |
| P55060 | Exportin-2 OS=Homo sap | 10.43 | 1.75 | 1 | 2 | 18.75 | 5.66 | 3 | 4 | 19.99 | 4.84 | 3 | 4 |
| Q02252 | Methylmalonate-semialdehyde dehydr | | 0.00 | | | | 0.00 | | | 8.81 | 7.10 | 2 | 2 |
| Q58FF8 | Putative heat shock prote | 13.02 | 7.09 | 2 | 3 | 4.82 | 3.94 | 1 | 1 | 5.02 | 3.94 | 1 | 1 |
| P00403 | Cytochrome c oxidase subunit 2 OS=H | | 0.00 | | | | 0.00 | | | 4.75 | 7.05 | 1 | 1 |
| Q15437 | Protein transport protein | 15.81 | 7.04 | 3 | 3 | 4.02 | 1.83 | 1 | 1 | 10.30 | 4.17 | 2 | 2 |
| Q13435 | Splicing factor 3B subunit | 15.30 | 4.92 | 2 | 3 | 5.75 | 2.12 | 1 | 1 | 5.00 | 1.68 | 1 | 1 |
| Q96C23 | Aldose 1-epimerase OS=H | 4.79 | 7.02 | 1 | 1 | | 0.00 | | | 5.49 | 7.02 | 1 | 1 |
| P30520 | Adenylosuccinate synthet | 8.85 | 7.02 | 2 | 2 | 4.72 | 4.61 | 1 | 1 | 4.51 | 4.61 | 1 | 1 |
| Q86V81 | THO complex subunit 4 O | 5.60 | 7.00 | 1 | 1 | 5.31 | 7.00 | 1 | 1 | 5.25 | 7.00 | 1 | 1 |
| O96019 | Actin-like protein 6A OS=Homo sapien | | 0.00 | | | 4.94 | 6.99 | 1 | 1 | 5.29 | 6.99 | 1 | 1 |
| Q9H7E9 | UPF0488 protein C8orf33 OS=Homo s | | 0.00 | | | | 0.00 | | | 4.30 | 6.99 | 1 | 1 |
| Q9BUF5 | Tubulin beta-6 chain OS= | 10.18 | 6.95 | 2 | 2 | 4.47 | 3.14 | 1 | 1 | 4.79 | 3.14 | 1 | 1 |

| | | | | | | | | | | | | | |
|---|---|---|---|---|---|---|---|---|---|---|---|---|---|
| Q9C0K7 | STE20-related kinase adapter protein | | 0.00 | | | | 0.00 | | | 5.07 | 6.94 | 1 | 1 |
| O43175 | D-3-phosphoglycerate de | 4.12 | 2.81 | 1 | 1 | | 0.00 | | | 5.00 | 4.13 | 1 | 1 |
| P16930 | Fumarylacetoacetase OS=Homo sapie | | 0.00 | | | | 0.00 | | | 8.88 | 6.92 | 2 | 2 |
| Q96E39 | RNA binding motif protein, X-linked-lik | | 0.00 | | | 4.11 | 3.33 | 1 | 1 | 4.14 | 3.59 | 1 | 1 |
| Q9Y230 | RuvB-like 2 OS=Homo sa | 8.98 | 6.91 | 2 | 2 | 4.68 | 3.46 | 1 | 1 | 4.21 | 3.46 | 1 | 1 |
| Q6ZVX7 | F-box only protein 50 OS=Homo sapie | | 0.00 | | | 5.21 | 6.91 | 1 | 1 | 5.20 | 6.91 | 1 | 1 |
| P40925 | Malate dehydrogenase, c | 8.18 | 6.89 | 2 | 2 | | 0.00 | | | | 0.00 | | |
| Q9GZS3 | WD repeat-containing pro | 4.81 | 6.89 | 1 | 1 | 4.13 | 6.89 | 1 | 1 | 4.61 | 6.89 | 1 | 1 |
| P82930 | 28S ribosomal protein S3 | 4.91 | 6.88 | 1 | 1 | | 0.00 | | | | 0.00 | | |
| Q6UW68 | Transmembrane protein : | 4.26 | 6.88 | 1 | 1 | | 0.00 | | | | 0.00 | | |
| P45974 | Ubiquitin carboxyl-termin | 9.13 | 3.96 | 2 | 2 | 12.13 | 5.13 | 2 | 3 | 8.68 | 5.13 | 2 | 2 |
| Q99436 | Proteasome subunit beta | 4.98 | 6.86 | 1 | 1 | 4.90 | 6.86 | 1 | 1 | 5.09 | 6.86 | 1 | 1 |
| P61313 | 60S ribosomal protein L15 OS=Homo | | 0.00 | | | 4.68 | 6.86 | 1 | 1 | | 0.00 | | |
| Q9UKL4 | Gap junction delta-2 protein OS=Hom | | 0.00 | | | 3.95 | 6.85 | 1 | 1 | | 0.00 | | |
| O95716 | Ras-related protein Rab-3D OS=Homo | | 0.00 | | | | 0.00 | | | 4.55 | 6.85 | 1 | 1 |
| Q99614 | Tetratricopeptide repeat | 5.10 | 6.85 | 1 | 1 | 5.09 | 6.85 | 1 | 1 | 5.24 | 6.85 | 1 | 1 |
| P17612 | cAMP-dependent protein | 5.15 | 6.84 | 1 | 1 | | 0.00 | | | 5.23 | 6.84 | 1 | 1 |
| Q13813 | Spectrin alpha chain, non | 13.97 | 2.22 | 3 | 3 | 20.60 | 3.11 | 4 | 4 | 19.58 | 3.07 | 4 | 4 |
| P37837 | Transaldolase OS=Homo | 3.92 | 3.56 | 1 | 1 | | 0.00 | | | 4.25 | 3.26 | 1 | 1 |
| Q8WXS5 | Voltage-dependent calcium channel ga | | 0.00 | | | | 0.00 | | | 5.64 | 6.82 | 1 | 1 |
| P31327 | Carbamoyl-phosphate syr | 23.19 | 6.80 | 5 | 5 | 14.24 | 3.60 | 3 | 3 | 13.84 | 3.27 | 3 | 3 |
| Q9UHX1 | Poly(U)-binding-splicing f | 4.65 | 3.94 | 1 | 1 | | 0.00 | | | 4.61 | 2.86 | 1 | 1 |
| Q13838 | Spliceosome RNA helicase | 8.93 | 6.78 | 2 | 2 | 4.35 | 3.27 | 1 | 1 | | 0.00 | | |
| P33316 | Deoxyuridine 5'-triphosph | 4.17 | 6.75 | 1 | 1 | | 0.00 | | | 4.64 | 6.75 | 1 | 1 |
| Q00688 | Peptidyl-prolyl cis-trans isomerase FKE | | 0.00 | | | | 0.00 | | | 4.71 | 6.70 | 1 | 1 |
| P07305 | Histone H1.0 OS=Homo s | 4.68 | 6.70 | 1 | 1 | 4.34 | 6.70 | 1 | 1 | | 0.00 | | |
| Q9Y6G9 | Cytoplasmic dynein 1 light intermediat | | 0.00 | | | 8.43 | 6.69 | 2 | 2 | 4.11 | 3.25 | 1 | 1 |
| P54819 | Adenylate kinase 2, mitochondrial OS= | | 0.00 | | | | 0.00 | | | 5.01 | 6.69 | 1 | 1 |
| Q96IU4 | Alpha/beta hydrolase domain-containi | | 0.00 | | | 3.93 | 6.67 | 1 | 1 | | 0.00 | | |
| Q9NRR5 | Ubiquilin-4 OS=Homo sapiens GN=UB | | 0.00 | | | 4.22 | 2.66 | 1 | 1 | 9.03 | 6.66 | 2 | 2 |
| A1L0T0 | Acetolactate synthase-like | 6.03 | 3.64 | 1 | 1 | 18.22 | 6.65 | 2 | 3 | 11.51 | 6.65 | 2 | 2 |
| Q8TCS8 | Polyribonucleotide nucleo | 19.38 | 4.60 | 2 | 4 | 8.70 | 4.34 | 2 | 2 | 5.01 | 2.30 | 1 | 1 |
| P30085 | UMP-CMP kinase OS=Homo sapiens G | | 0.00 | | | | 0.00 | | | 4.30 | 6.63 | 1 | 1 |
| P52292 | Importin subunit alpha-1 | 10.10 | 6.62 | 2 | 2 | 10.45 | 6.62 | 2 | 2 | 9.23 | 6.62 | 2 | 2 |
| Q3LXA3 | Bifunctional ATP-depende | 9.06 | 6.61 | 2 | 2 | 4.65 | 2.96 | 1 | 1 | | 0.00 | | |
| O75208 | Ubiquinone biosynthesis protein COQ9 | | 0.00 | | | 4.92 | 6.60 | 1 | 1 | | 0.00 | | |
| Q53H82 | Beta-lactamase-like protein 2 OS=Hor | | 0.00 | | | 4.91 | 6.60 | 1 | 1 | | 0.00 | | |
| P27105 | Erythrocyte band 7 integral membrane | | 0.00 | | | 6.58 | 6.60 | 1 | 1 | 5.98 | 6.60 | 1 | 1 |
| P36639 | 7,8-dihydro-8-oxoguanine | 3.88 | 6.60 | 1 | 1 | | 0.00 | | | | 0.00 | | |

| | | | | | | | | | | | | | |
|---|---|---|---|---|---|---|---|---|---|---|---|---|---|
| O75251 | NADH dehydrogenase [ul | 3.97 | 6.57 | 1 | 1 | | 0.00 | | | | 0.00 | | |
| Q96TA1 | Niban-like protein 1 OS=I | 14.65 | 4.42 | 2 | 3 | 5.45 | 2.28 | 1 | 1 | 5.09 | 2.14 | 1 | 1 |
| P17655 | Calpain-2 catalytic subuni | 16.32 | 6.57 | 3 | 3 | 10.67 | 6.57 | 2 | 2 | 9.42 | 2.14 | 1 | 2 |
| Q96GG9 | DCN1-like protein 1 OS=I | 5.18 | 6.56 | 1 | 1 | 4.84 | 6.56 | 1 | 1 | 4.32 | 6.56 | 1 | 1 |
| Q13151 | Heterogeneous nuclear ribonucleoprot | 0.00 | | | | | 0.00 | | | 5.74 | 6.56 | 1 | 1 |
| P04259 | Keratin, type II cytoskeletal 6B OS=H | 0.00 | | | | 12.67 | 4.43 | 2 | 3 | 4.42 | 2.13 | 1 | 1 |
| Q9BRX8 | Redox-regulatory protein | 4.02 | 6.55 | 1 | 1 | | 0.00 | | | 4.34 | 6.55 | 1 | 1 |
| Q99598 | Translin-associated prote | 4.55 | 6.55 | 1 | 1 | | 0.00 | | | | 0.00 | | |
| Q9NUJ1 | Mycophenolic acid acyl-gl | 4.93 | 6.54 | 1 | 1 | 5.45 | 6.54 | 1 | 1 | 5.05 | 6.54 | 1 | 1 |
| A6NIZ1 | Ras-related protein Rap-1 | 8.67 | 6.52 | 1 | 2 | 4.13 | 6.52 | 1 | 1 | | 0.00 | | |
| O60716 | Catenin delta-1 OS=Hom | 9.74 | 4.96 | 2 | 2 | | 0.00 | | | 9.57 | 4.55 | 2 | 2 |
| O15266 | Short stature homeobox protein OS=H | 0.00 | | | | 3.92 | 6.51 | 1 | 1 | | 0.00 | | |
| Q9NP79 | Vacuolar protein sorting-a | 5.95 | 6.51 | 1 | 1 | 5.42 | 6.51 | 1 | 1 | | 0.00 | | |
| A0MZ66 | Shootin-1 OS=Homo sapi | 4.62 | 3.17 | 1 | 1 | 9.93 | 6.50 | 2 | 2 | 8.46 | 6.50 | 2 | 2 |
| P27694 | Replication protein A 70 kDa DNA-binc | 0.00 | | | | 4.64 | 3.08 | 1 | 1 | 4.34 | 3.41 | 1 | 1 |
| Q9BZZ5 | Apoptosis inhibitor 5 OS=Homo sapier | 0.00 | | | | 4.72 | 3.05 | 1 | 1 | 10.04 | 3.44 | 1 | 2 |
| Q9Y5K8 | V-type proton ATPase subunit D OS=H | 0.00 | | | | | 0.00 | | | 5.43 | 6.48 | 1 | 1 |
| Q15785 | Mitochondrial import rece | 5.44 | 6.47 | 1 | 1 | 9.70 | 6.47 | 1 | 2 | 4.76 | 6.47 | 1 | 1 |
| P62701 | 40S ribosomal protein S4 | 3.97 | 6.46 | 1 | 1 | 4.07 | 6.46 | 1 | 1 | 4.28 | 6.46 | 1 | 1 |
| P36955 | Pigment epithelium-deriv | 9.12 | 6.46 | 2 | 2 | 4.53 | 3.35 | 1 | 1 | 4.59 | 3.35 | 1 | 1 |
| Q587J8 | KHDC3-like protein OS=H | 4.01 | 6.45 | 1 | 1 | | 0.00 | | | | 0.00 | | |
| P06730 | Eukaryotic translation init | 3.90 | 6.45 | 1 | 1 | | 0.00 | | | | 0.00 | | |
| Q9BXR0 | Queuine tRNA-ribosyltran | 5.21 | 6.45 | 1 | 1 | | 0.00 | | | | 0.00 | | |
| P33176 | Kinesin-1 heavy chain OS | 4.17 | 1.56 | 1 | 1 | 3.96 | 1.56 | 1 | 1 | 13.52 | 4.88 | 3 | 3 |
| P61247 | 40S ribosomal protein S3 | 10.40 | 6.44 | 1 | 2 | 5.24 | 6.44 | 1 | 1 | 15.06 | 6.44 | 1 | 3 |
| P53041 | Serine/threonine-protein phosphatase | 0.00 | | | | 5.05 | 3.81 | 1 | 1 | 9.24 | 6.41 | 2 | 2 |
| O00567 | Nucleolar protein 56 OS= | 9.75 | 6.40 | 2 | 2 | | 0.00 | | | 10.07 | 3.20 | 1 | 2 |
| Q9HC38 | Glyoxalase domain-contai | 4.49 | 6.39 | 1 | 1 | | 0.00 | | | | 0.00 | | |
| P41227 | N-alpha-acetyltransferase 10 OS=Hom | 0.00 | | | | 4.23 | 6.38 | 1 | 1 | | 0.00 | | |
| Q13144 | Translation initiation facto | 5.24 | 3.88 | 1 | 1 | | 0.00 | | | 4.39 | 2.50 | 1 | 1 |
| P39656 | Dolichyl-diphosphooligosa | 4.83 | 6.36 | 1 | 1 | | 0.00 | | | | 0.00 | | |
| Q9HBA9 | Putative N-acetylated-alp | 4.76 | 6.33 | 1 | 1 | | 0.00 | | | | 0.00 | | |
| P04179 | Superoxide dismutase [M | 3.82 | 6.31 | 1 | 1 | | 0.00 | | | | 0.00 | | |
| Q9Y2Z0 | Suppressor of G2 allele o | 5.57 | 6.30 | 1 | 1 | | 0.00 | | | 11.51 | 6.30 | 1 | 2 |
| P31146 | Coronin-1A OS=Homo sapiens GN=CC | 0.00 | | | | 5.17 | 6.29 | 1 | 1 | 5.04 | 6.29 | 1 | 1 |
| P04626 | Receptor tyrosine-protein | 21.00 | 5.10 | 3 | 4 | 4.78 | 1.51 | 1 | 1 | 10.19 | 3.11 | 2 | 2 |
| P30153 | Serine/threonine-protein | 9.43 | 6.28 | 2 | 2 | 10.28 | 3.06 | 1 | 2 | 9.28 | 6.28 | 2 | 2 |
| P50213 | Isocitrate dehydrogenase [NAD] subu | 0.00 | | | | | 0.00 | | | 4.60 | 6.28 | 1 | 1 |
| P00505 | Aspartate aminotransfera | 8.11 | 6.28 | 2 | 2 | 3.91 | 3.49 | 1 | 1 | | 0.00 | | |

| | | | | | | | | | | | |
|---|---|---|---|---|---|---|---|---|---|---|---|
| P48444 | Coatomer subunit delta C | 5.29 | 6.26 | 1 | 1 | | 0.00 | | | 5.68 | 6.26 | 1 | 1 |
| P41252 | Isoleucine--tRNA ligase, c | 8.66 | 3.41 | 2 | 2 | 9.46 | 2.93 | 2 | 2 | 14.62 | 4.28 | 3 | 3 |
| P30041 | Peroxiredoxin-6 OS=Homo sapiens GN | 0.00 | | | | 3.91 | 6.25 | 1 | 1 | 4.82 | 6.25 | 1 | 1 |
| P14678 | Small nuclear ribonucleop | 3.96 | 6.25 | 1 | 1 | | 0.00 | | | | 0.00 | | |
| P23526 | Adenosylhomocysteinase | 8.80 | 6.25 | 2 | 2 | 4.72 | 3.01 | 1 | 1 | | 0.00 | | |
| P62917 | 60S ribosomal protein L8 OS=Homo s | 0.00 | | | | | 0.00 | | | 4.34 | 6.23 | 1 | 1 |
| P30038 | Delta-1-pyrroline-5-carbo | 13.82 | 6.22 | 2 | 3 | 9.69 | 6.22 | 2 | 2 | 9.12 | 6.22 | 2 | 2 |
| P37235 | Hippocalcin-like protein 1 | 4.20 | 6.22 | 1 | 1 | | 0.00 | | | | 0.00 | | |
| Q9Y262 | Eukaryotic translation init | 9.34 | 6.21 | 2 | 2 | | 0.00 | | | 4.74 | 3.55 | 1 | 1 |
| O95299 | NADH dehydrogenase [ul | 4.91 | 6.20 | 1 | 1 | 6.09 | 6.20 | 1 | 1 | 5.26 | 6.20 | 1 | 1 |
| P60510 | Serine/threonine-protein | 6.04 | 6.19 | 1 | 1 | | 0.00 | | | 4.97 | 6.19 | 1 | 1 |
| O75534 | Cold shock domain-conta | 5.45 | 2.26 | 1 | 1 | 4.53 | 2.26 | 1 | 1 | 8.56 | 3.88 | 2 | 2 |
| P42566 | Epidermal growth factor r | 9.40 | 3.91 | 2 | 2 | 4.82 | 2.23 | 1 | 1 | 5.55 | 2.12 | 1 | 1 |
| O00505 | Importin subunit alpha-4 | 4.09 | 3.45 | 1 | 1 | 4.10 | 2.69 | 1 | 1 | 4.91 | 2.69 | 1 | 1 |
| P21333 | Filamin-A OS=Homo sapi | 33.80 | 4.61 | 7 | 7 | 23.20 | 3.59 | 5 | 5 | 39.09 | 4.23 | 6 | 8 |
| O75494 | Serine/arginine-rich splicing factor 10 | 0.00 | | | | | 0.00 | | | 4.42 | 6.11 | 1 | 1 |
| Q16473 | Putative tenascin-XA OS=Homo sapier | 0.00 | | | | 4.76 | 6.11 | 1 | 1 | | 0.00 | | |
| P61758 | Prefoldin subunit 3 OS=H | 3.86 | 6.09 | 1 | 1 | | 0.00 | | | | 0.00 | | |
| Q68D91 | Metallo-beta-lactamase d | 3.82 | 6.09 | 1 | 1 | | 0.00 | | | | 0.00 | | |
| P27635 | 60S ribosomal protein L10 OS=Homo | 0.00 | | | | 4.61 | 6.07 | 1 | 1 | | 0.00 | | |
| O15160 | DNA-directed RNA polym | 4.87 | 6.07 | 1 | 1 | | 0.00 | | | | 0.00 | | |
| Q96DB5 | Regulator of microtubule | 5.85 | 6.05 | 1 | 1 | | 0.00 | | | 5.78 | 6.05 | 1 | 1 |
| P67936 | Tropomyosin alpha-4 chain OS=Homo | 0.00 | | | | | 0.00 | | | 10.09 | 6.05 | 1 | 2 |
| Q92841 | Probable ATP-dependent | 8.71 | 3.70 | 2 | 2 | 5.27 | 2.06 | 1 | 1 | 13.52 | 4.39 | 2 | 3 |
| P62753 | 40S ribosomal protein S6 | 4.91 | 6.02 | 1 | 1 | 4.35 | 6.02 | 1 | 1 | 4.28 | 6.02 | 1 | 1 |
| Q9NVA1 | Ubiquinol-cytochrome-c reductase con | 0.00 | | | | 4.07 | 6.02 | 1 | 1 | | 0.00 | | |
| Q9NYP9 | Protein Mis18-alpha OS=Homo sapien | 0.00 | | | | 4.08 | 6.01 | 1 | 1 | | 0.00 | | |
| Q13126 | S-methyl-5'-thioadenosine | 4.32 | 6.01 | 1 | 1 | 4.46 | 6.01 | 1 | 1 | | 0.00 | | |
| P26196 | Probable ATP-dependent RNA helicase | 0.00 | | | | 5.87 | 6.00 | 1 | 1 | 5.42 | 6.00 | 1 | 1 |
| Q92734 | Protein TFG OS=Homo sapiens GN=T | 0.00 | | | | | 0.00 | | | 4.39 | 6.00 | 1 | 1 |
| P02647 | Apolipoprotein A-I OS=H | 4.23 | 5.99 | 1 | 1 | | 0.00 | | | | 0.00 | | |
| Q12972 | Nuclear inhibitor of protei | 4.44 | 5.98 | 1 | 1 | 4.47 | 5.98 | 1 | 1 | | 0.00 | | |
| Q96JY6 | PDZ and LIM domain pro | 4.00 | 5.97 | 1 | 1 | 3.93 | 5.97 | 1 | 1 | | 0.00 | | |
| Q8NC56 | LEM domain-containing p | 3.97 | 2.58 | 1 | 1 | 3.91 | 3.38 | 1 | 1 | | 0.00 | | |
| Q13217 | DnaJ homolog subfamily | 5.54 | 5.95 | 1 | 1 | | 0.00 | | | 5.95 | 5.95 | 1 | 1 |
| Q9Y3I0 | tRNA-splicing ligase RtcB | 6.55 | 5.94 | 1 | 1 | 6.75 | 5.94 | 1 | 1 | | 0.00 | | |
| Q9BR76 | Coronin-1B OS=Homo sapiens GN=CC | 0.00 | | | | 5.46 | 5.93 | 1 | 1 | 5.40 | 5.93 | 1 | 1 |
| Q8TAT6 | Nuclear protein localizatic | 14.10 | 5.92 | 2 | 3 | 15.11 | 5.92 | 2 | 3 | 11.30 | 2.96 | 1 | 2 |
| O00232 | 26S proteasome non-ATP | 4.95 | 2.85 | 1 | 1 | 12.61 | 5.92 | 2 | 3 | 4.52 | 2.85 | 1 | 1 |

| | | | | | | | | | | | | | |
|---|---|---|---|---|---|---|---|---|---|---|---|---|---|
| Q9Y285 | Phenylalanine--tRNA ligas | 9.04 | 5.91 | 2 | 2 | 8.51 | 5.91 | 2 | 2 | 5.37 | 2.76 | 1 | 1 |
| P21964 | Catechol O-methyltransfe | 9.15 | 5.90 | 2 | 2 | 5.27 | 5.90 | 1 | 1 | 5.03 | 5.90 | 1 | 1 |
| P43246 | DNA mismatch repair protein Msh2 OS | 0.00 | | | | | 0.00 | | | 14.63 | 5.89 | 3 | 3 |
| Q6PID8 | Kelch domain-containing protein 10 O: | 0.00 | | | | | 0.00 | | | 4.40 | 5.88 | 1 | 1 |
| P24539 | ATP synthase F(0) complex subunit B: | 0.00 | | | | 3.96 | 5.86 | 1 | 1 | | 0.00 | | |
| Q9NUQ9 | Protein FAM49B OS=Hom | 4.33 | 5.86 | 1 | 1 | | 0.00 | | | 4.72 | 5.86 | 1 | 1 |
| P35606 | Coatomer subunit beta' C | 8.19 | 3.97 | 2 | 2 | 3.94 | 1.88 | 1 | 1 | | 0.00 | | |
| Q96GM1 | Lipid phosphate phosphatase-related | 0.00 | | | | | 0.00 | | | 4.18 | 5.83 | 1 | 1 |
| Q14764 | Major vault protein OS=H | 7.75 | 3.92 | 2 | 2 | 4.51 | 1.79 | 1 | 1 | 4.15 | 1.90 | 1 | 1 |
| P20618 | Proteasome subunit beta | 4.77 | 5.81 | 1 | 1 | | 0.00 | | | | 0.00 | | |
| Q13564 | NEDD8-activating enzyme E1 regulato | 0.00 | | | | 4.81 | 5.81 | 1 | 1 | 6.51 | 5.81 | 1 | 1 |
| O95678 | Keratin, type II cytoskeletal 75 OS=Hc | 0.00 | | | | | 0.00 | | | 8.68 | 5.81 | 2 | 2 |
| Q13148 | TAR DNA-binding protein 43 OS=Hom | 0.00 | | | | 6.24 | 5.80 | 1 | 1 | | 0.00 | | |
| Q9BWD1 | Acetyl-CoA acetyltransferase, cytosolic | 0.00 | | | | | 0.00 | | | 4.95 | 5.79 | 1 | 1 |
| Q9Y2R9 | 28S ribosomal protein S7 | 4.25 | 5.79 | 1 | 1 | 4.32 | 5.79 | 1 | 1 | 4.33 | 5.79 | 1 | 1 |
| Q52LJ0 | Protein FAM98B OS=Hom | 4.73 | 5.76 | 1 | 1 | | 0.00 | | | 4.96 | 5.76 | 1 | 1 |
| P16401 | Histone H1.5 OS=Homo s | 4.28 | 5.75 | 1 | 1 | | 0.00 | | | 4.19 | 5.75 | 1 | 1 |
| P17812 | CTP synthase 1 OS=Hom | 6.03 | 3.05 | 1 | 1 | 10.45 | 5.75 | 2 | 2 | 11.26 | 5.75 | 2 | 2 |
| Q309B1 | Tripartite motif-containing protein 16- | 0.00 | | | | 4.23 | 5.75 | 1 | 1 | | 0.00 | | |
| Q7Z434 | Mitochondrial antiviral-signaling protei | 0.00 | | | | 4.85 | 5.74 | 1 | 1 | | 0.00 | | |
| P52788 | Spermine synthase OS=H | 5.92 | 5.74 | 1 | 1 | 5.41 | 5.74 | 1 | 1 | 5.31 | 5.74 | 1 | 1 |
| Q6PID6 | Tetratricopeptide repeat protein 33 OS | 0.00 | | | | | 0.00 | | | 4.44 | 5.73 | 1 | 1 |
| Q9H9J2 | 39S ribosomal protein L4 | 4.08 | 5.72 | 1 | 1 | | 0.00 | | | 4.17 | 5.72 | 1 | 1 |
| Q99729 | Heterogeneous nuclear ri | 5.59 | 5.72 | 1 | 1 | 4.82 | 5.72 | 1 | 1 | | 0.00 | | |
| Q6P1N9 | Putative deoxyribonuclease TATDN1 C | 0.00 | | | | | 0.00 | | | 4.57 | 5.72 | 1 | 1 |
| P21912 | Succinate dehydrogenase | 5.14 | 5.71 | 1 | 1 | | 0.00 | | | | 0.00 | | |
| P13797 | Plastin-3 OS=Homo sapie | 4.88 | 2.22 | 1 | 1 | | 0.00 | | | 10.20 | 3.49 | 1 | 2 |
| Q9Y570 | Protein phosphatase metl | 4.87 | 5.70 | 1 | 1 | | 0.00 | | | | 0.00 | | |
| P25786 | Proteasome subunit alpha type-1 OS= | 0.00 | | | | | 0.00 | | | 4.26 | 5.70 | 1 | 1 |
| O43709 | Probable 18S rRNA (guanine-N(7))-me | 0.00 | | | | 4.01 | 5.69 | 1 | 1 | | 0.00 | | |
| Q99674 | Cell growth regulator witl | 3.91 | 5.65 | 1 | 1 | | 0.00 | | | | 0.00 | | |
| Q15124 | Phosphoglucomutase-like | 13.08 | 5.64 | 2 | 3 | 8.77 | 5.64 | 2 | 2 | 4.49 | 2.82 | 1 | 1 |
| P07996 | Thrombospondin-1 OS=H | 13.67 | 4.19 | 3 | 3 | 10.54 | 3.25 | 2 | 2 | | 0.00 | | |
| Q9P0L0 | Vesicle-associated membrane protein- | 0.00 | | | | 4.80 | 5.62 | 1 | 1 | 4.45 | 5.62 | 1 | 1 |
| Q92882 | Osteoclast-stimulating fac | 4.25 | 5.61 | 1 | 1 | | 0.00 | | | 4.25 | 5.61 | 1 | 1 |
| Q8TAQ2 | SWI/SNF complex subuni | 9.08 | 2.97 | 2 | 2 | 4.05 | 1.24 | 1 | 1 | 5.66 | 2.64 | 1 | 1 |
| P46108 | Adapter molecule crk OS= | 4.49 | 5.59 | 1 | 1 | | 0.00 | | | 4.82 | 5.59 | 1 | 1 |
| Q9HB07 | UPF0160 protein MYG1, r | 4.96 | 5.59 | 1 | 1 | | 0.00 | | | 5.52 | 5.59 | 1 | 1 |
| Q16543 | Hsp90 co-chaperone Cdc37 OS=Homo | 0.00 | | | | 4.72 | 5.56 | 1 | 1 | 9.69 | 5.56 | 1 | 2 |

| | | | | | | | | | | | | | |
|---|---|---|---|---|---|---|---|---|---|---|---|---|---|
| P04183 | Thymidine kinase, cytoso | 4.77 | 5.56 | 1 | 1 | | 0.00 | | | | 0.00 | | |
| Q9UKM9 | RNA-binding protein Raly OS=Homo s | | 0.00 | | | | 0.00 | | | 4.53 | 5.56 | 1 | 1 |
| Q92530 | Proteasome inhibitor PI31 subunit OS= | | 0.00 | | | | 0.00 | | | 4.22 | 5.54 | 1 | 1 |
| P55263 | Adenosine kinase OS=Ho | 10.11 | 5.52 | 1 | 2 | 10.20 | 5.52 | 1 | 2 | 5.05 | 5.52 | 1 | 1 |
| O95571 | Persulfide dioxygenase E | 4.20 | 5.51 | 1 | 1 | | 0.00 | | | 4.12 | 5.51 | 1 | 1 |
| P61026 | Ras-related protein Rab-1 | 4.21 | 5.50 | 1 | 1 | 3.96 | 5.50 | 1 | 1 | 4.28 | 5.50 | 1 | 1 |
| P43304 | Glycerol-3-phosphate deh | 4.78 | 2.48 | 1 | 1 | 9.67 | 5.50 | 2 | 2 | | 0.00 | | |
| P55039 | Developmentally-regulated GTP-bindir | | 0.00 | | | | 0.00 | | | 5.10 | 5.49 | 1 | 1 |
| Q5TC12 | ATP synthase mitochondr | 5.52 | 5.49 | 1 | 1 | 4.93 | 5.49 | 1 | 1 | | 0.00 | | |
| P37058 | Testosterone 17-beta-deh | 3.89 | 5.48 | 1 | 1 | | 0.00 | | | | 0.00 | | |
| P48507 | Glutamate--cysteine ligase regulatory | | 0.00 | | | | 0.00 | | | 4.38 | 5.47 | 1 | 1 |
| P30048 | Thioredoxin-dependent p | 4.27 | 5.47 | 1 | 1 | | 0.00 | | | | 0.00 | | |
| O00764 | Pyridoxal kinase OS=Hom | 5.37 | 5.45 | 1 | 1 | 5.19 | 5.45 | 1 | 1 | 4.45 | 5.45 | 1 | 1 |
| Q9H993 | UPF0364 protein C6orf211 OS=Homo | | 0.00 | | | | 0.00 | | | 5.06 | 5.44 | 1 | 1 |
| Q99961 | Endophilin-A2 OS=Homo sapiens GN= | | 0.00 | | | | 0.00 | | | 5.86 | 5.43 | 1 | 1 |
| Q9UMX0 | Ubiquilin-1 OS=Homo sapiens GN=UB | | 0.00 | | | 4.22 | 2.72 | 1 | 1 | 10.16 | 5.43 | 2 | 2 |
| Q9NQR4 | Omega-amidase NIT2 OS=Homo sapi | | 0.00 | | | | 0.00 | | | 4.85 | 5.43 | 1 | 1 |
| Q15393 | Splicing factor 3B subunit | 9.27 | 2.63 | 2 | 2 | 4.60 | 1.40 | 1 | 1 | 13.67 | 4.19 | 3 | 3 |
| Q9P015 | 39S ribosomal protein L15, mitochond | | 0.00 | | | 3.97 | 5.41 | 1 | 1 | | 0.00 | | |
| Q8NBF2 | NHL repeat-containing pr | 8.85 | 5.37 | 2 | 2 | 9.95 | 3.44 | 1 | 2 | 10.35 | 3.44 | 1 | 2 |
| Q14847 | LIM and SH3 domain pro | 4.07 | 5.36 | 1 | 1 | | 0.00 | | | | 0.00 | | |
| P49903 | Selenide, water dikinase | 5.23 | 5.36 | 1 | 1 | | 0.00 | | | | 0.00 | | |
| Q8IVW6 | AT-rich interactive domain-containing | | 0.00 | | | | 0.00 | | | 5.01 | 5.35 | 1 | 1 |
| P52272 | Heterogeneous nuclear ri | 4.53 | 2.19 | 1 | 1 | | 0.00 | | | 5.32 | 3.15 | 1 | 1 |
| P51884 | Lumican OS=Homo sapie | 4.52 | 5.33 | 1 | 1 | 4.62 | 5.33 | 1 | 1 | 9.91 | 5.33 | 1 | 2 |
| Q7Z6J2 | General receptor for phos | 3.88 | 5.32 | 1 | 1 | | 0.00 | | | | 0.00 | | |
| P07858 | Cathepsin B OS=Homo sapiens GN=C | | 0.00 | | | 4.99 | 5.31 | 1 | 1 | 4.26 | 5.31 | 1 | 1 |
| Q9H7Z7 | Prostaglandin E synthase | 4.69 | 5.31 | 1 | 1 | | 0.00 | | | 9.80 | 5.31 | 1 | 2 |
| P41091 | Eukaryotic translation initiation factor | | 0.00 | | | | 0.00 | | | 4.25 | 5.30 | 1 | 1 |
| P02533 | Keratin, type I cytoskelet | 7.76 | 5.08 | 2 | 2 | 8.63 | 5.30 | 2 | 2 | 4.18 | 3.18 | 1 | 1 |
| P55735 | Protein SEC13 homolog C | 4.35 | 5.28 | 1 | 1 | 4.95 | 5.28 | 1 | 1 | | 0.00 | | |
| Q8ND83 | SLAIN motif-containing p | 4.80 | 5.28 | 1 | 1 | | 0.00 | | | | 0.00 | | |
| P18615 | Negative elongation factor E OS=Hom | | 0.00 | | | 5.49 | 5.26 | 1 | 1 | 10.27 | 5.26 | 1 | 2 |
| Q99442 | Translocation protein SEC | 3.83 | 5.26 | 1 | 1 | | 0.00 | | | | 0.00 | | |
| Q16181 | Septin-7 OS=Homo sapiens GN=SEPT | | 0.00 | | | 4.99 | 5.26 | 1 | 1 | | 0.00 | | |
| Q14204 | Cytoplasmic dynein 1 hea | 38.77 | 3.16 | 8 | 8 | 32.77 | 2.48 | 6 | 7 | 38.11 | 2.91 | 8 | 8 |
| P49841 | Glycogen synthase kinase-3 beta OS= | | 0.00 | | | | 0.00 | | | 4.36 | 5.24 | 1 | 1 |
| P43897 | Elongation factor Ts, mitc | 4.70 | 5.23 | 1 | 1 | 4.86 | 5.23 | 1 | 1 | 4.91 | 5.23 | 1 | 1 |
| P53990 | IST1 homolog OS=Homo sapiens GN= | | 0.00 | | | 5.04 | 5.22 | 1 | 1 | | 0.00 | | |

| | | | | | | | | | | | | | |
|---|---|---|---|---|---|---|---|---|---|---|---|---|---|
| P26373 | 60S ribosomal protein L1 | 4.01 | 5.21 | 1 | 1 | 3.93 | 5.21 | 1 | 1 | 4.22 | 5.21 | 1 | 1 |
| O95793 | Double-stranded RNA-bin | 4.01 | 2.43 | 1 | 1 | | 0.00 | | | 5.35 | 2.77 | 1 | 1 |
| O43396 | Thioredoxin-like protein 1 | 3.91 | 5.19 | 1 | 1 | | 0.00 | | | 5.12 | 5.19 | 1 | 1 |
| Q9UBQ7 | Glyoxylate reductase/hyd | 5.45 | 5.18 | 1 | 1 | | 0.00 | | | | 0.00 | | |
| Q9H0A0 | N-acetyltransferase 10 OS | 9.95 | 3.71 | 2 | 2 | 9.41 | 3.51 | 2 | 2 | 10.01 | 2.05 | 1 | 2 |
| P36871 | Phosphoglucomutase-1 C | 9.04 | 5.16 | 2 | 2 | 9.27 | 5.16 | 2 | 2 | 4.50 | 2.85 | 1 | 1 |
| O95983 | Methyl-CpG-binding doma | 3.89 | 5.15 | 1 | 1 | | 0.00 | | | | 0.00 | | |
| Q15424 | Scaffold attachment facto | 4.90 | 2.84 | 1 | 1 | | 0.00 | | | 10.21 | 2.30 | 2 | 2 |
| P29144 | Tripeptidyl-peptidase 2 O | 10.44 | 2.88 | 2 | 2 | 20.31 | 5.12 | 3 | 4 | 16.25 | 5.12 | 3 | 3 |
| P50895 | Basal cell adhesion molecule OS=Hom | 0.00 | | | | | 0.00 | | | 5.01 | 5.10 | 1 | 1 |
| P16422 | Epithelial cell adhesion m | 4.36 | 5.10 | 1 | 1 | 4.47 | 5.10 | 1 | 1 | | 0.00 | | |
| O94776 | Metastasis-associated protein MTA2 O | 0.00 | | | | 8.58 | 5.09 | 2 | 2 | | 0.00 | | |
| Q15738 | Sterol-4-alpha-carboxylat | 4.04 | 5.09 | 1 | 1 | | 0.00 | | | | 0.00 | | |
| Q8NFV4 | Alpha/beta hydrolase dor | 4.49 | 5.08 | 1 | 1 | | 0.00 | | | 5.20 | 5.08 | 1 | 1 |
| P08779 | Keratin, type I cytoskelet | 8.17 | 4.86 | 2 | 2 | 4.14 | 2.11 | 1 | 1 | | 0.00 | | |
| Q9NPF4 | Probable tRNA N6-adenosine threonyl | 0.00 | | | | 3.99 | 5.07 | 1 | 1 | | 0.00 | | |
| O75533 | Splicing factor 3B subunit | 12.80 | 5.06 | 3 | 3 | | 0.00 | | | 9.39 | 2.76 | 2 | 2 |
| Q9Y6Z7 | Collectin-10 OS=Homo sapiens GN=C | 0.00 | | | | | 0.00 | | | 4.18 | 5.05 | 1 | 1 |
| Q9H4A6 | Golgi phosphoprotein 3 OS=Homo sap | 0.00 | | | | 4.36 | 5.03 | 1 | 1 | 5.18 | 5.03 | 1 | 1 |
| P11940 | Polyadenylate-binding pro | 9.45 | 5.03 | 2 | 2 | 9.47 | 5.03 | 2 | 2 | 5.62 | 2.67 | 1 | 1 |
| O14734 | Acyl-coenzyme A thioeste | 4.28 | 5.02 | 1 | 1 | | 0.00 | | | | 0.00 | | |
| P54709 | Sodium/potassium-transporting ATPas | 0.00 | | | | 4.13 | 5.02 | 1 | 1 | | 0.00 | | |
| P28288 | ATP-binding cassette sub | 6.22 | 2.88 | 1 | 1 | 10.04 | 5.01 | 2 | 2 | | 0.00 | | |
| Q14722 | Voltage-gated potassium | 3.99 | 5.01 | 1 | 1 | | 0.00 | | | | 0.00 | | |
| P33993 | DNA replication licensing | 4.81 | 3.06 | 1 | 1 | 4.42 | 1.95 | 1 | 1 | 4.30 | 1.95 | 1 | 1 |
| Q06430 | N-acetyllactosaminide bet | 4.98 | 5.00 | 1 | 1 | | 0.00 | | | | 0.00 | | |
| Q15435 | Protein phosphatase 1 re | 5.04 | 5.00 | 1 | 1 | | 0.00 | | | | 0.00 | | |
| P80303 | Nucleobindin-2 OS=Homo sapiens GN | 0.00 | | | | 4.81 | 5.00 | 1 | 1 | | 0.00 | | |
| Q8IZ26 | Zinc finger protein 34 OS=Homo sapie | 0.00 | | | | | 0.00 | | | 5.18 | 5.00 | 1 | 1 |
| Q8NBJ4 | Golgi membrane protein 1 OS=Homo | 0.00 | | | | | 0.00 | | | 4.85 | 4.99 | 1 | 1 |
| O60763 | General vesicular transpo | 4.64 | 1.87 | 1 | 1 | 4.28 | 1.87 | 1 | 1 | 8.88 | 3.12 | 2 | 2 |
| Q8N2F6 | Armadillo repeat-containi | 4.43 | 4.96 | 1 | 1 | | 0.00 | | | | 0.00 | | |
| P12429 | Annexin A3 OS=Homo sapiens GN=AI | 0.00 | | | | | 0.00 | | | 4.24 | 4.95 | 1 | 1 |
| P09758 | Tumor-associated calcium signal trans | 0.00 | | | | 4.20 | 4.95 | 1 | 1 | 4.36 | 4.95 | 1 | 1 |
| P78527 | DNA-dependent protein k | 57.01 | 3.95 | 11 | 13 | 32.51 | 2.69 | 7 | 7 | 27.38 | 2.33 | 6 | 6 |
| Q12824 | SWI/SNF-related matrix-associated ac | 0.00 | | | | | 0.00 | | | 4.27 | 4.94 | 1 | 1 |
| Q92979 | Ribosomal RNA small sub | 3.94 | 4.92 | 1 | 1 | | 0.00 | | | | 0.00 | | |
| Q9UBE0 | SUMO-activating enzyme subunit 1 OS | 0.00 | | | | 5.39 | 4.91 | 1 | 1 | 5.01 | 4.91 | 1 | 1 |
| Q9NUE0 | Palmitoyltransferase ZDHHC18 OS=Hc | 0.00 | | | | 3.97 | 4.90 | 1 | 1 | | 0.00 | | |

| | | | | | | | | | | | | | |
|---|---|---|---|---|---|---|---|---|---|---|---|---|---|
| Q04637 | Eukaryotic translation init | 18.09 | 4.88 | 4 | 4 | 5.25 | 2.13 | 1 | 1 | 4.66 | 1.06 | 1 | 1 |
| Q7L266 | Isoaspartyl peptidase/L-a | 4.16 | 4.87 | 1 | 1 | | 0.00 | | | | 0.00 | | |
| Q5VW32 | BRO1 domain-containing protein BRO) | 0.00 | | | | | 0.00 | | | 4.98 | 4.87 | 1 | 1 |
| O95218 | Zinc finger Ran-binding domain-contai | 0.00 | | | | | 0.00 | | | 4.29 | 4.85 | 1 | 1 |
| O00515 | Ladinin-1 OS=Homo sapi | 4.02 | 2.32 | 1 | 1 | | 0.00 | | | 4.44 | 2.51 | 1 | 1 |
| Q9BYV8 | Centrosomal protein of 41 kDa OS=Hc | 0.00 | | | | | 0.00 | | | 5.85 | 4.83 | 1 | 1 |
| Q9BTE3 | Mini-chromosome mainte | 5.18 | 4.83 | 1 | 1 | | 0.00 | | | | 0.00 | | |
| O00273 | DNA fragmentation factor subunit alph | 0.00 | | | | | 0.00 | | | 4.33 | 4.83 | 1 | 1 |
| Q9NWS0 | PIH1 domain-containing | 4.10 | 4.83 | 1 | 1 | | 0.00 | | | | 0.00 | | |
| Q99460 | 26S proteasome non-ATP | 6.68 | 2.73 | 1 | 1 | 9.96 | 4.83 | 2 | 2 | 5.24 | 2.10 | 1 | 1 |
| Q9Y4P1 | Cysteine protease ATG4B OS=Homo s | 0.00 | | | | | 0.00 | | | 4.24 | 4.83 | 1 | 1 |
| Q53GQ0 | Estradiol 17-beta-dehydrogenase 12 C | 0.00 | | | | 4.15 | 4.81 | 1 | 1 | 4.63 | 4.81 | 1 | 1 |
| Q12904 | Aminoacyl tRNA synthase | 3.84 | 4.81 | 1 | 1 | | 0.00 | | | | 0.00 | | |
| Q7KZ85 | Transcription elongation f | 6.13 | 1.33 | 1 | 1 | 4.73 | 1.10 | 1 | 1 | 15.36 | 3.71 | 3 | 3 |
| A8MU46 | Smoothelin-like protein 1 OS=Homo s | 0.00 | | | | 4.12 | 4.81 | 1 | 1 | | 0.00 | | |
| P55265 | Double-stranded RNA-spe | 18.63 | 3.59 | 3 | 4 | 9.49 | 2.69 | 2 | 2 | 4.27 | 1.22 | 1 | 1 |
| Q9H2U2 | Inorganic pyrophosphata | 5.51 | 4.79 | 1 | 1 | | 0.00 | | | | 0.00 | | |
| O00541 | Pescadillo homolog OS=Homo sapiens | 0.00 | | | | | 0.00 | | | 5.06 | 4.76 | 1 | 1 |
| Q9UMS4 | Pre-mRNA-processing factor 19 OS=H | 0.00 | | | | 4.35 | 4.76 | 1 | 1 | | 0.00 | | |
| Q9NW64 | Pre-mRNA-splicing factor | 4.66 | 4.76 | 1 | 1 | | 0.00 | | | | 0.00 | | |
| Q86TX2 | Acyl-coenzyme A thioeste | 5.01 | 4.75 | 1 | 1 | 3.92 | 4.75 | 1 | 1 | 5.36 | 4.75 | 1 | 1 |
| P49354 | Protein farnesyltransferas | 5.04 | 4.75 | 1 | 1 | 9.27 | 4.75 | 1 | 2 | 9.47 | 4.75 | 1 | 2 |
| Q2M2Z5 | Centrosomal protein kizuna OS=Homo | 0.00 | | | | 5.56 | 4.75 | 1 | 1 | | 0.00 | | |
| Q16531 | DNA damage-binding pro | 6.47 | 2.11 | 1 | 1 | | 0.00 | | | 11.63 | 4.74 | 2 | 2 |
| P50219 | Motor neuron and pancre | 3.81 | 4.74 | 1 | 1 | | 0.00 | | | | 0.00 | | |
| Q86VP6 | Cullin-associated NEDD8- | 12.97 | 3.50 | 3 | 3 | 13.00 | 2.36 | 2 | 3 | | 0.00 | | |
| Q86SH2 | Zygote arrest protein 1 OS=Homo sap | 0.00 | | | | 4.19 | 4.72 | 1 | 1 | | 0.00 | | |
| P25788 | Proteasome subunit alpha | 4.07 | 4.71 | 1 | 1 | | 0.00 | | | | 0.00 | | |
| P46777 | 60S ribosomal protein L5 | 4.31 | 4.71 | 1 | 1 | 4.74 | 4.71 | 1 | 1 | 4.54 | 4.71 | 1 | 1 |
| P25205 | DNA replication licensing | 4.84 | 2.48 | 1 | 1 | | 0.00 | | | 5.89 | 2.23 | 1 | 1 |
| Q02218 | 2-oxoglutarate dehydroge | 13.34 | 4.69 | 3 | 3 | | 0.00 | | | | 0.00 | | |
| Q96PZ0 | Pseudouridylate synthase | 4.66 | 2.57 | 1 | 1 | 4.35 | 2.57 | 1 | 1 | 9.19 | 4.69 | 2 | 2 |
| O15144 | Actin-related protein 2/3 complex subu | 0.00 | | | | 4.44 | 4.67 | 1 | 1 | | 0.00 | | |
| A6NDG6 | Phosphoglycolate phosph | 3.90 | 4.67 | 1 | 1 | 4.47 | 4.67 | 1 | 1 | 4.40 | 4.67 | 1 | 1 |
| Q7Z4P5 | Growth/differentiation factor 7 OS=Hc | 0.00 | | | | 3.91 | 4.67 | 1 | 1 | | 0.00 | | |
| Q9H8Y8 | Golgi reassembly-stacking protein 2 O | 0.00 | | | | 8.34 | 4.65 | 1 | 2 | | 0.00 | | |
| Q8N3X6 | Ligand-dependent nuclear receptor co | 0.00 | | | | 4.87 | 4.65 | 1 | 1 | | 0.00 | | |
| P54727 | UV excision repair protein | 9.13 | 4.65 | 1 | 2 | 8.95 | 4.65 | 1 | 2 | | 0.00 | | |
| P49585 | Choline-phosphate cytidy | 4.39 | 4.63 | 1 | 1 | | 0.00 | | | 5.74 | 4.63 | 1 | 1 |

| | | | | | | | | | | | | | |
|---|---|---|---|---|---|---|---|---|---|---|---|---|---|
| P31350 | Ribonucleoside-diphospha | 4.12 | 4.63 | 1 | 1 | 4.11 | 4.63 | 1 | 1 | 4.37 | 4.63 | 1 | 1 |
| O95365 | Zinc finger and BTB domain-containing | 0.00 | | | | | 0.00 | | | 5.13 | 4.62 | 1 | 1 |
| Q8IZA3 | Histone H1oo OS=Homo | 4.00 | 4.62 | 1 | 1 | | 0.00 | | | | 0.00 | | |
| Q14161 | ARF GTPase-activating protein GIT2 C | 0.00 | | | | 4.80 | 4.61 | 1 | 1 | | 0.00 | | |
| Q6P9A2 | Polypeptide N-acetylgalactosaminyltra | 0.00 | | | | | 0.00 | | | 5.01 | 4.61 | 1 | 1 |
| Q6ZN04 | RNA-binding protein MEX | 5.20 | 4.57 | 1 | 1 | | 0.00 | | | | 0.00 | | |
| Q07960 | Rho GTPase-activating protein 1 OS=H | 0.00 | | | | | 0.00 | | | 4.38 | 4.56 | 1 | 1 |
| Q9NP92 | 28S ribosomal protein S3 | 4.46 | 4.56 | 1 | 1 | 4.72 | 4.56 | 1 | 1 | | 0.00 | | |
| O14579 | Coatomer subunit epsilon | 3.97 | 4.55 | 1 | 1 | 4.03 | 4.55 | 1 | 1 | 4.47 | 4.55 | 1 | 1 |
| Q9H3P7 | Golgi resident protein GCP60 OS=Hom | 0.00 | | | | | 0.00 | | | 11.88 | 4.55 | 1 | 2 |
| Q9Y276 | Mitochondrial chaperone BCS1 OS=Ho | 0.00 | | | | | 0.00 | | | 4.15 | 4.53 | 1 | 1 |
| Q9Y6E2 | Basic leucine zipper and W | 5.30 | 4.53 | 1 | 1 | 5.16 | 4.53 | 1 | 1 | 4.45 | 4.53 | 1 | 1 |
| Q9Y266 | Nuclear migration protein | 4.03 | 4.53 | 1 | 1 | | 0.00 | | | | 0.00 | | |
| Q3ZCQ8 | Mitochondrial import inner membrane | 0.00 | | | | 8.31 | 4.53 | 1 | 2 | | 0.00 | | |
| Q06124 | Tyrosine-protein phospha | 4.00 | 2.01 | 1 | 1 | 4.02 | 2.51 | 1 | 1 | 4.23 | 2.51 | 1 | 1 |
| Q15165 | Serum paraoxonase/arylesterase 2 OS | 0.00 | | | | | 0.00 | | | 4.18 | 4.52 | 1 | 1 |
| Q9C0C2 | 182 kDa tankyrase-1-binc | 8.68 | 2.20 | 2 | 2 | 8.24 | 1.97 | 2 | 2 | 5.28 | 1.45 | 1 | 1 |
| O14908 | PDZ domain-containing p | 4.25 | 4.50 | 1 | 1 | | 0.00 | | | | 0.00 | | |
| O15231 | Zinc finger protein 185 O | 4.81 | 4.50 | 1 | 1 | 5.79 | 4.50 | 1 | 1 | | 0.00 | | |
| O00300 | Tumor necrosis factor receptor superfa | 0.00 | | | | | 0.00 | | | 4.39 | 4.49 | 1 | 1 |
| Q8IXZ3 | Transcription factor Sp8 OS=Homo sa | 0.00 | | | | | 0.00 | | | 4.48 | 4.49 | 1 | 1 |
| P10515 | Dihydrolipoyllysine-residu | 8.99 | 4.48 | 2 | 2 | | 0.00 | | | 4.87 | 2.32 | 1 | 1 |
| Q9UBS4 | DnaJ homolog subfamily B member 1 | 0.00 | | | | | 0.00 | | | 4.27 | 4.47 | 1 | 1 |
| Q9Y365 | PCTP-like protein OS=Ho | 3.99 | 4.47 | 1 | 1 | 4.77 | 4.47 | 1 | 1 | 4.56 | 4.47 | 1 | 1 |
| Q9UHB9 | Signal recognition particle | 3.95 | 2.07 | 1 | 1 | 8.29 | 4.47 | 2 | 2 | 4.20 | 2.07 | 1 | 1 |
| Q7L0Y3 | Mitochondrial ribonucleas | 4.32 | 4.47 | 1 | 1 | | 0.00 | | | | 0.00 | | |
| P10644 | cAMP-dependent protein | 4.34 | 4.46 | 1 | 1 | | 0.00 | | | 4.43 | 4.46 | 1 | 1 |
| P31321 | cAMP-dependent protein kinase type I | 0.00 | | | | 4.22 | 4.46 | 1 | 1 | 5.06 | 4.46 | 1 | 1 |
| Q9BST9 | Rhotekin OS=Homo sapie | 4.95 | 4.44 | 1 | 1 | | 0.00 | | | 4.99 | 4.44 | 1 | 1 |
| Q05655 | Protein kinase C delta typ | 8.89 | 4.44 | 2 | 2 | | 0.00 | | | | 0.00 | | |
| Q9NYK5 | 39S ribosomal protein L39, mitochond | 0.00 | | | | 3.95 | 4.44 | 1 | 1 | | 0.00 | | |
| Q9NVA2 | Septin-11 OS=Homo sapiens GN=SEP | 0.00 | | | | | 0.00 | | | 5.07 | 4.43 | 1 | 1 |
| Q9NW68 | BSD domain-containing p | 3.94 | 4.42 | 1 | 1 | | 0.00 | | | 4.80 | 4.42 | 1 | 1 |
| O43837 | Isocitrate dehydrogenase [NAD] subu | 0.00 | | | | | 0.00 | | | 5.04 | 4.42 | 1 | 1 |
| Q6P9B9 | Integrator complex subur | 5.53 | 4.42 | 1 | 1 | | 0.00 | | | | 0.00 | | |
| Q00325 | Phosphate carrier protein, mitochondr | 0.00 | | | | | 0.00 | | | 4.19 | 4.42 | 1 | 1 |
| P26368 | Splicing factor U2AF 65 k | 5.02 | 4.42 | 1 | 1 | | 0.00 | | | 5.67 | 4.42 | 1 | 1 |
| Q14151 | Scaffold attachment facto | 4.90 | 2.73 | 1 | 1 | | 0.00 | | | 4.20 | 1.68 | 1 | 1 |
| Q92820 | Gamma-glutamyl hydrola | 9.76 | 4.40 | 1 | 2 | 4.80 | 4.40 | 1 | 1 | 4.96 | 4.40 | 1 | 1 |

| | | | | | | | | | | | | | |
|---|---|---|---|---|---|---|---|---|---|---|---|---|---|
| Q8TC12 | Retinol dehydrogenase 11 OS=Homo | | 0.00 | | | | 0.00 | | | 4.53 | 4.40 | 1 | 1 |
| O00571 | ATP-dependent RNA helic | 8.91 | 4.38 | 2 | 2 | 4.50 | 1.81 | 1 | 1 | 9.08 | 4.38 | 2 | 2 |
| P12236 | ADP/ATP translocase 3 OS=Homo sap | | 0.00 | | | | 0.00 | | | 4.41 | 4.36 | 1 | 1 |
| P05141 | ADP/ATP translocase 2 O | 4.80 | 4.36 | 1 | 1 | 4.79 | 4.36 | 1 | 1 | 4.48 | 4.36 | 1 | 1 |
| P22059 | Oxysterol-binding protein | 3.92 | 2.35 | 1 | 1 | | 0.00 | | | 9.15 | 4.34 | 2 | 2 |
| P53621 | Coatomer subunit alpha ( | 10.19 | 3.10 | 2 | 2 | 4.98 | 1.31 | 1 | 1 | 14.57 | 3.02 | 2 | 3 |
| P12081 | Histidine--tRNA ligase, cytoplasmic OS | | 0.00 | | | | 0.00 | | | 5.32 | 4.32 | 1 | 1 |
| P04424 | Argininosuccinate lyase OS=Homo sap | | 0.00 | | | 4.31 | 4.31 | 1 | 1 | 4.53 | 4.31 | 1 | 1 |
| Q6YN16 | Hydroxysteroid dehydrog | 4.80 | 4.31 | 1 | 1 | | 0.00 | | | 4.22 | 4.31 | 1 | 1 |
| P42126 | Enoyl-CoA delta isomeras | 4.34 | 4.30 | 1 | 1 | | 0.00 | | | 4.19 | 4.30 | 1 | 1 |
| O75907 | Diacylglycerol O-acyltrans | 4.13 | 4.30 | 1 | 1 | 9.17 | 4.30 | 1 | 2 | | 0.00 | | |
| O14929 | Histone acetyltransferase | 4.61 | 4.30 | 1 | 1 | | 0.00 | | | | 0.00 | | |
| Q9NVD7 | Alpha-parvin OS=Homo s | 3.93 | 4.30 | 1 | 1 | | 0.00 | | | | 0.00 | | |
| P14324 | Farnesyl pyrophosphate s | 3.84 | 4.30 | 1 | 1 | 7.96 | 4.30 | 1 | 2 | 4.30 | 4.30 | 1 | 1 |
| P33908 | Mannosyl-oligosaccharide 1,2-alpha-m | | 0.00 | | | 5.25 | 4.29 | 1 | 1 | | 0.00 | | |
| Q9H3N1 | Thioredoxin-related transmembrane p | | 0.00 | | | | 0.00 | | | 4.20 | 4.29 | 1 | 1 |
| Q92733 | Proline-rich protein PRCC OS=Homo s | | 0.00 | | | 4.42 | 4.28 | 1 | 1 | | 0.00 | | |
| P16278 | Beta-galactosidase OS=H | 7.75 | 2.22 | 1 | 2 | 4.30 | 2.22 | 1 | 1 | 8.57 | 4.28 | 2 | 2 |
| Q8N2K0 | Monoacylglycerol lipase ABHD12 OS=I | | 0.00 | | | 4.56 | 4.27 | 1 | 1 | 4.13 | 4.27 | 1 | 1 |
| Q96A49 | Synapse-associated prote | 4.30 | 4.26 | 1 | 1 | | 0.00 | | | | 0.00 | | |
| P47897 | Glutamine--tRNA ligase C | 8.81 | 4.26 | 2 | 2 | 4.99 | 2.06 | 1 | 1 | | 0.00 | | |
| Q58FF3 | Putative endoplasmin-like protein OS= | | 0.00 | | | 4.05 | 4.26 | 1 | 1 | | 0.00 | | |
| Q00341 | Vigilin OS=Homo sapiens | 15.39 | 2.84 | 2 | 3 | 9.20 | 2.84 | 2 | 2 | 9.70 | 3.00 | 2 | 2 |
| Q9NUQ7 | Ufm1-specific protease 2 OS=Homo sa | | 0.00 | | | | 0.00 | | | 4.25 | 4.26 | 1 | 1 |
| Q9NXG2 | THUMP domain-containing protein 1 C | | 0.00 | | | 4.78 | 4.25 | 1 | 1 | 4.69 | 4.25 | 1 | 1 |
| P08754 | Guanine nucleotide-bindir | 4.15 | 4.24 | 1 | 1 | 4.54 | 4.24 | 1 | 1 | | 0.00 | | |
| Q9NR30 | Nucleolar RNA helicase 2 | 10.27 | 4.21 | 2 | 2 | 4.74 | 2.17 | 1 | 1 | 4.43 | 2.17 | 1 | 1 |
| P39748 | Flap endonuclease 1 OS= | 3.98 | 4.21 | 1 | 1 | | 0.00 | | | 4.38 | 4.21 | 1 | 1 |
| P21283 | V-type proton ATPase sul | 4.11 | 4.19 | 1 | 1 | | 0.00 | | | | 0.00 | | |
| P61964 | WD repeat-containing protein 5 OS=H | | 0.00 | | | | 0.00 | | | 4.20 | 4.19 | 1 | 1 |
| P34897 | Serine hydroxymethyltrar | 5.09 | 4.17 | 1 | 1 | 5.00 | 4.17 | 1 | 1 | 4.80 | 4.17 | 1 | 1 |
| Q8IVD9 | NudC domain-containing | 4.47 | 4.16 | 1 | 1 | | 0.00 | | | 9.04 | 4.16 | 1 | 2 |
| P46926 | Glucosamine-6-phosphate | 4.08 | 4.15 | 1 | 1 | | 0.00 | | | | 0.00 | | |
| Q15061 | WD repeat-containing protein 43 OS= | | 0.00 | | | 6.28 | 4.14 | 1 | 1 | | 0.00 | | |
| Q92575 | UBX domain-containing protein 4 OS= | | 0.00 | | | 4.42 | 4.13 | 1 | 1 | | 0.00 | | |
| Q9Y2X9 | Zinc finger protein 281 O | 4.75 | 4.13 | 1 | 1 | | 0.00 | | | | 0.00 | | |
| P08621 | U1 small nuclear ribonucleoprotein 70 | | 0.00 | | | 4.36 | 4.12 | 1 | 1 | 4.99 | 4.12 | 1 | 1 |
| Q9Y371 | Endophilin-B1 OS=Homo | 3.92 | 4.11 | 1 | 1 | 3.93 | 4.11 | 1 | 1 | | 0.00 | | |
| O75306 | NADH dehydrogenase [ul | 3.94 | 4.10 | 1 | 1 | 4.75 | 4.10 | 1 | 1 | 4.18 | 4.10 | 1 | 1 |

| | | | | | | | | | | | | | |
|---|---|---|---|---|---|---|---|---|---|---|---|---|---|
| A8MVM7 | Putative uncharacterized protein ENSP | | 0.00 | | | 5.07 | 4.10 | 1 | 1 | | 0.00 | | |
| O43602 | Neuronal migration protein doublecort | | 0.00 | | | 4.29 | 4.08 | 1 | 1 | | 0.00 | | |
| Q02750 | Dual specificity mitogen-activated prot | | 0.00 | | | 4.37 | 4.07 | 1 | 1 | 4.50 | 4.07 | 1 | 1 |
| P09543 | 2',3'-cyclic-nucleotide 3'-p | 4.77 | 4.04 | 1 | 1 | 4.53 | 4.04 | 1 | 1 | 5.24 | 4.04 | 1 | 1 |
| Q9NQ29 | Putative RNA-binding pro | 4.53 | 4.04 | 1 | 1 | | 0.00 | | | | 0.00 | | |
| P26640 | Valine--tRNA ligase OS=H | 8.53 | 2.77 | 2 | 2 | | 0.00 | | | 8.91 | 2.85 | 2 | 2 |
| P14923 | Junction plakoglobin OS=Homo sapier | | 0.00 | | | 5.13 | 4.03 | 1 | 1 | | 0.00 | | |
| P51114 | Fragile X mental retardati | 12.17 | 4.03 | 1 | 2 | 4.72 | 4.03 | 1 | 1 | 6.14 | 4.03 | 1 | 1 |
| Q96GQ7 | Probable ATP-dependent RNA helicase | | 0.00 | | | | 0.00 | | | 9.22 | 4.02 | 2 | 2 |
| P11117 | Lysosomal acid phosphat | 4.15 | 4.02 | 1 | 1 | | 0.00 | | | | 0.00 | | |
| Q9Y2P8 | RNA 3'-terminal phosphat | 4.33 | 4.02 | 1 | 1 | | 0.00 | | | | 0.00 | | |
| Q96GA3 | Protein LTV1 homolog OS | 3.96 | 4.00 | 1 | 1 | | 0.00 | | | | 0.00 | | |
| Q9UGV2 | Protein NDRG3 OS=Homo sapiens GN | | 0.00 | | | 4.46 | 4.00 | 1 | 1 | | 0.00 | | |
| Q9BW91 | ADP-ribose pyrophosphat | 4.09 | 4.00 | 1 | 1 | | 0.00 | | | | 0.00 | | |
| Q9BPX6 | Calcium uptake protein 1, mitochondri | | 0.00 | | | | 0.00 | | | 4.30 | 3.99 | 1 | 1 |
| Q8IVT2 | Mitotic interactor and substrate of PLK | | 0.00 | | | 9.86 | 3.98 | 1 | 2 | | 0.00 | | |
| P04278 | Sex hormone-binding glo | 4.07 | 3.98 | 1 | 1 | | 0.00 | | | 4.62 | 3.98 | 1 | 1 |
| P61011 | Signal recognition particle 54 kDa prot | | 0.00 | | | | 0.00 | | | 5.37 | 3.97 | 1 | 1 |
| Q8WUA8 | Tsukushin OS=Homo sap | 4.31 | 3.97 | 1 | 1 | | | | | | | | |
| P07099 | Epoxide hydrolase 1 OS= | 5.02 | 3.96 | 1 | 1 | | 0.00 | | | | 0.00 | | |
| Q96C03 | Mitochondrial dynamics p | 4.01 | 3.96 | 1 | 1 | | 0.00 | | | | 0.00 | | |
| Q9BZE4 | Nucleolar GTP-binding protein 1 OS=H | | 0.00 | | | 5.19 | 3.94 | 1 | 1 | 11.48 | 3.94 | 1 | 2 |
| P19971 | Thymidine phosphorylase OS=Homo s | | 0.00 | | | | 0.00 | | | 4.47 | 3.94 | 1 | 1 |
| Q96EP5 | DAZ-associated protein 1 | 5.36 | 3.93 | 1 | 1 | 5.71 | 3.93 | 1 | 1 | 4.84 | 3.93 | 1 | 1 |
| P37287 | Phosphatidylinositol N-acetylglucosam | | 0.00 | | | | 0.00 | | | 4.85 | 3.93 | 1 | 1 |
| Q9UI12 | V-type proton ATPase sul | 4.89 | 3.93 | 1 | 1 | 4.65 | 3.93 | 1 | 1 | 4.65 | 3.93 | 1 | 1 |
| O95202 | LETM1 and EF-hand dom | 4.31 | 1.62 | 1 | 1 | 4.66 | 1.62 | 1 | 1 | 8.85 | 3.92 | 2 | 2 |
| P49257 | Protein ERGIC-53 OS=Homo sapiens ( | | 0.00 | | | | 0.00 | | | 5.00 | 3.92 | 1 | 1 |
| Q96K80 | Zinc finger CCCH domain-containing p | | 0.00 | | | | 0.00 | | | 4.11 | 3.92 | 1 | 1 |
| Q8N4J0 | UPF0586 protein C9orf41 | 3.93 | 3.91 | 1 | 1 | | 0.00 | | | | 0.00 | | |
| Q9H490 | Phosphatidylinositol glyca | 5.13 | 3.91 | 1 | 1 | | 0.00 | | | 4.36 | 3.91 | 1 | 1 |
| Q92890 | Ubiquitin fusion degradation protein 1 | | 0.00 | | | | 0.00 | | | 4.10 | 3.91 | 1 | 1 |
| Q14566 | DNA replication licensing | 4.36 | 1.83 | 1 | 1 | 4.86 | 2.07 | 1 | 1 | | 0.00 | | |
| P06737 | Glycogen phosphorylase, | 8.79 | 2.13 | 1 | 2 | 4.07 | 1.77 | 1 | 1 | | 0.00 | | |
| Q8N336 | ELMO domain-containing protein 1 OS | | 0.00 | | | | 0.00 | | | 4.26 | 3.89 | 1 | 1 |
| O76021 | Ribosomal L1 domain-containing prote | | 0.00 | | | | 0.00 | | | 4.97 | 3.88 | 1 | 1 |
| Q15599 | Na(+)/H(+) exchange regulatory cofa | | 0.00 | | | 4.25 | 3.86 | 1 | 1 | | 0.00 | | |
| Q9NYU2 | UDP-glucose:glycoproteir | 10.71 | 2.89 | 2 | 2 | | 0.00 | | | 4.80 | 0.96 | 1 | 1 |
| Q9NUG4 | Cerebral cavernous malfc | 3.84 | 3.85 | 1 | 1 | | 0.00 | | | | 0.00 | | |

| | | | | | | | | | | | | | |
|---|---|---|---|---|---|---|---|---|---|---|---|---|---|
| P12270 | Nucleoprotein TPR OS=H | 14.28 | 2.16 | 3 | 3 | 4.07 | 0.63 | 1 | 1 | 5.11 | 1.06 | 1 | 1 |
| Q9Y2R4 | Probable ATP-dependent RNA helicase | | 0.00 | | | | 0.00 | | | 5.16 | 3.84 | 1 | 1 |
| Q9P287 | BRCA2 and CDKN1A-inter | 3.82 | 3.82 | 1 | 1 | | 0.00 | | | 4.12 | 3.82 | 1 | 1 |
| P61160 | Actin-related protein 2 OS=Homo sapi | | 0.00 | | | | 0.00 | | | 4.53 | 3.81 | 1 | 1 |
| O14979 | Heterogeneous nuclear ri | 4.87 | 3.81 | 1 | 1 | | 0.00 | | | 4.64 | 3.81 | 1 | 1 |
| A6NHL2 | Tubulin alpha chain-like 3 | 4.05 | 3.81 | 1 | 1 | | 0.00 | | | | 0.00 | | |
| O14983 | Sarcoplasmic/endoplasmi | 5.04 | 2.30 | 1 | 1 | 4.28 | 1.50 | 1 | 1 | 4.83 | 2.30 | 1 | 1 |
| P31153 | S-adenosylmethionine syı | 4.63 | 3.80 | 1 | 1 | 4.88 | 3.80 | 1 | 1 | 4.38 | 3.80 | 1 | 1 |
| Q8TE77 | Protein phosphatase Slingshot homolo | | 0.00 | | | 4.98 | 3.79 | 1 | 1 | | 0.00 | | |
| O75643 | U5 small nuclear ribonucl | 9.16 | 1.92 | 2 | 2 | 14.42 | 2.81 | 3 | 3 | | 0.00 | | |
| Q8IYB3 | Serine/arginine repetitive matrix prote | | 0.00 | | | 5.10 | 3.76 | 1 | 1 | 5.19 | 3.76 | 1 | 1 |
| P36269 | Gamma-glutamyltransferase 5 OS=Ho | | 0.00 | | | 5.01 | 3.75 | 1 | 1 | | 0.00 | | |
| Q7Z5L9 | Interferon regulatory fact | 5.21 | 3.75 | 1 | 1 | | 0.00 | | | | 0.00 | | |
| Q01082 | Spectrin beta chain, non- | 15.19 | 2.16 | 3 | 3 | 9.82 | 1.44 | 2 | 2 | 15.59 | 2.37 | 3 | 3 |
| Q14344 | Guanine nucleotide-bindi | 3.90 | 3.71 | 1 | 1 | | 0.00 | | | | 0.00 | | |
| O95396 | Adenylyltransferase and s | 5.41 | 3.70 | 1 | 1 | | 0.00 | | | 5.41 | 3.70 | 1 | 1 |
| Q92769 | Histone deacetylase 2 OS | 3.89 | 3.69 | 1 | 1 | | 0.00 | | | | 0.00 | | |
| Q9BS26 | Endoplasmic reticulum re | 4.48 | 3.69 | 1 | 1 | 4.64 | 3.69 | 1 | 1 | 5.15 | 3.69 | 1 | 1 |
| Q15075 | Early endosome antigen 1 | 4.74 | 1.13 | 1 | 1 | 5.68 | 1.20 | 1 | 1 | 15.84 | 3.69 | 3 | 3 |
| Q27J81 | Inverted formin-2 OS=Homo sapiens ( | | 0.00 | | | 4.90 | 2.16 | 1 | 1 | 5.73 | 1.52 | 1 | 1 |
| P49321 | Nuclear autoantigenic spe | 9.82 | 3.68 | 2 | 2 | 5.60 | 2.03 | 1 | 1 | 9.86 | 3.68 | 2 | 2 |
| Q8NFH3 | Nucleoporin Nup43 OS=Homo sapiens | | 0.00 | | | 4.01 | 3.68 | 1 | 1 | | 0.00 | | |
| P35250 | Replication factor C subu | 4.45 | 3.67 | 1 | 1 | | 0.00 | | | | 0.00 | | |
| Q01844 | RNA-binding protein EWS | 5.22 | 3.66 | 1 | 1 | | 0.00 | | | | 0.00 | | |
| P43034 | Platelet-activating factor a | 8.22 | 3.66 | 1 | 2 | | 0.00 | | | 4.40 | 3.66 | 1 | 1 |
| P49356 | Protein farnesyltransferase subunit be | | 0.00 | | | | 0.00 | | | 4.58 | 3.66 | 1 | 1 |
| Q9NSE4 | Isoleucine--tRNA ligase, r | 9.63 | 3.66 | 2 | 2 | | 0.00 | | | 5.26 | 1.98 | 1 | 1 |
| Q7Z2W4 | Zinc finger CCCH-type antiviral protein | | 0.00 | | | 9.62 | 2.00 | 1 | 2 | 9.74 | 3.66 | 2 | 2 |
| Q01970 | 1-phosphatidylinositol 4,5 | 10.41 | 3.65 | 2 | 2 | 5.47 | 1.46 | 1 | 1 | 4.96 | 1.46 | 1 | 1 |
| Q6P2Q9 | Pre-mRNA-processing-spl | 19.71 | 3.64 | 4 | 4 | | 0.00 | | | 5.58 | 0.99 | 1 | 1 |
| P08686 | Steroid 21-hydroxylase O | 4.14 | 3.64 | 1 | 1 | | 0.00 | | | | 0.00 | | |
| O75530 | Polycomb protein EED OS | 4.42 | 3.63 | 1 | 1 | | 0.00 | | | | 0.00 | | |
| P41219 | Peripherin OS=Homo sap | 4.18 | 3.62 | 1 | 1 | | 0.00 | | | | 0.00 | | |
| Q9NYV4 | Cyclin-dependent kinase 12 OS=Homo | | 0.00 | | | | 0.00 | | | 10.92 | 3.62 | 2 | 2 |
| Q9P289 | Serine/threonine-protein | 4.25 | 3.61 | 1 | 1 | | 0.00 | | | | 0.00 | | |
| O15213 | WD repeat-containing protein 46 OS= | | 0.00 | | | | 0.00 | | | 4.42 | 3.61 | 1 | 1 |
| A2A3N6 | Putative PIP5K1A and PS | 4.65 | 1.97 | 1 | 1 | 4.10 | 1.62 | 1 | 1 | | 0.00 | | |
| P48637 | Glutathione synthetase O | 4.17 | 3.59 | 1 | 1 | | 0.00 | | | | 0.00 | | |
| Q96PK6 | RNA-binding protein 14 OS=Homo sap | | 0.00 | | | 4.71 | 3.59 | 1 | 1 | | 0.00 | | |

| | | | | | | | | | | | | | |
|---|---|---|---|---|---|---|---|---|---|---|---|---|---|
| Q9NVH1 | DnaJ homolog subfamily | 4.55 | 3.58 | 1 | 1 | 4.37 | 3.58 | 1 | 1 | 4.65 | 3.58 | 1 | 1 |
| Q8NFF5 | FAD synthase OS=Homo sapiens GN= | | 0.00 | | | 4.62 | 3.58 | 1 | 1 | 4.62 | 3.58 | 1 | 1 |
| O15382 | Branched-chain-amino-ac | 3.90 | 3.57 | 1 | 1 | | 0.00 | | | | 0.00 | | |
| P51116 | Fragile X mental retardation syndrome | | 0.00 | | | | 0.00 | | | 5.51 | 3.57 | 1 | 1 |
| Q92917 | G patch domain and KOW | 3.88 | 3.57 | 1 | 1 | | 0.00 | | | | 0.00 | | |
| Q96SK2 | Transmembrane protein 209 OS=Hom | | 0.00 | | | | 0.00 | | | 4.92 | 3.57 | 1 | 1 |
| Q96NJ3 | Zinc finger protein 285 OS=Homo sap | | 0.00 | | | 4.74 | 3.56 | 1 | 1 | | 0.00 | | |
| Q9UNS2 | COP9 signalosome complex subunit 3 | | 0.00 | | | 4.49 | 3.55 | 1 | 1 | 4.43 | 3.55 | 1 | 1 |
| Q8N8S7 | Protein enabled homolog | 4.03 | 3.55 | 1 | 1 | | 0.00 | | | | 0.00 | | |
| Q01650 | Large neutral amino acids transporter | | 0.00 | | | 3.98 | 3.55 | 1 | 1 | 4.24 | 3.55 | 1 | 1 |
| O96013 | Serine/threonine-protein | 3.88 | 3.55 | 1 | 1 | | 0.00 | | | | 0.00 | | |
| P11172 | Uridine 5'-monophosphat | 3.90 | 3.54 | 1 | 1 | | 0.00 | | | 4.17 | 3.54 | 1 | 1 |
| A1A5D9 | Bicaudal D-related protein 2 OS=Hom | | 0.00 | | | | 0.00 | | | 4.26 | 3.54 | 1 | 1 |
| Q9UN86 | Ras GTPase-activating protein-binding | | 0.00 | | | 4.43 | 3.53 | 1 | 1 | | 0.00 | | |
| P52294 | Importin subunit alpha-5 | 4.05 | 3.53 | 1 | 1 | | 0.00 | | | | 0.00 | | |
| P51648 | Fatty aldehyde dehydrog | 4.13 | 3.51 | 1 | 1 | 3.93 | 3.51 | 1 | 1 | 4.22 | 3.51 | 1 | 1 |
| Q6DD88 | Atlastin-3 OS=Homo sapi | 6.31 | 3.51 | 1 | 1 | 4.01 | 3.51 | 1 | 1 | 5.55 | 3.51 | 1 | 1 |
| Q5TDH0 | Protein DDI1 homolog 2 ( | 3.84 | 3.51 | 1 | 1 | | 0.00 | | | | 0.00 | | |
| Q05519 | Serine/arginine-rich splici | 4.31 | 3.51 | 1 | 1 | | 0.00 | | | | 0.00 | | |
| Q01831 | DNA repair protein complementing XP | | 0.00 | | | 5.99 | 3.51 | 1 | 1 | | 0.00 | | |
| Q15172 | Serine/threonine-protein phosphatase | | 0.00 | | | 4.29 | 3.50 | 1 | 1 | 4.31 | 3.09 | 1 | 1 |
| Q15067 | Peroxisomal acyl-coenzyn | 4.63 | 3.48 | 1 | 1 | | 0.00 | | | 5.16 | 3.48 | 1 | 1 |
| P55010 | Eukaryotic translation init | 4.96 | 3.48 | 1 | 1 | 9.78 | 3.48 | 1 | 2 | 5.13 | 3.48 | 1 | 1 |
| Q9GZM8 | Nuclear distribution protein nudE-like | | 0.00 | | | 4.07 | 3.48 | 1 | 1 | | 0.00 | | |
| P35269 | General transcription factor IIF subuni | | 0.00 | | | | 0.00 | | | 4.24 | 3.48 | 1 | 1 |
| P40222 | Alpha-taxilin OS=Homo s | 4.45 | 3.48 | 1 | 1 | 5.24 | 3.48 | 1 | 1 | 5.60 | 3.48 | 1 | 1 |
| P35659 | Protein DEK OS=Homo sa | 4.36 | 3.47 | 1 | 1 | | 0.00 | | | | 0.00 | | |
| Q01085 | Nucleolysin TIAR OS=Ho | 3.93 | 3.47 | 1 | 1 | | 0.00 | | | | 0.00 | | |
| P26639 | Threonine--tRNA ligase, cytoplasmic C | | 0.00 | | | 3.91 | 1.80 | 1 | 1 | 4.15 | 1.66 | 1 | 1 |
| O60784 | Target of Myb protein 1 OS=Homo sa | | 0.00 | | | | 0.00 | | | 4.65 | 3.46 | 1 | 1 |
| Q13177 | Serine/threonine-protein kinase PAK 2 | | 0.00 | | | | 0.00 | | | 5.24 | 3.44 | 1 | 1 |
| Q9H1V8 | Sodium-dependent neutral amino acid | | 0.00 | | | 4.73 | 3.44 | 1 | 1 | | 0.00 | | |
| O60701 | UDP-glucose 6-dehydrog | 4.05 | 3.44 | 1 | 1 | 4.02 | 3.44 | 1 | 1 | 9.56 | 3.44 | 1 | 2 |
| P23588 | Eukaryotic translation initiation factor | | 0.00 | | | 9.71 | 3.44 | 1 | 2 | 15.02 | 3.44 | 1 | 3 |
| Q9Y2X3 | Nucleolar protein 58 OS= | 8.76 | 3.40 | 1 | 2 | 4.63 | 3.40 | 1 | 1 | 5.43 | 3.40 | 1 | 1 |
| O75475 | PC4 and SFRS1-interactir | 4.32 | 3.40 | 1 | 1 | | 0.00 | | | 5.63 | 3.40 | 1 | 1 |
| Q86WB0 | Nuclear-interacting partn | 3.89 | 3.39 | 1 | 1 | | 0.00 | | | | 0.00 | | |
| Q08945 | FACT complex subunit SSRP1 OS=Hor | | 0.00 | | | 5.67 | 3.39 | 1 | 1 | | 0.00 | | |
| Q9Y6E0 | Serine/threonine-protein | 5.69 | 3.39 | 1 | 1 | | 0.00 | | | | 0.00 | | |

| | | | | | | | | | | | | | |
|---|---|---|---|---|---|---|---|---|---|---|---|---|---|
| Q5FYA8 | Arylsulfatase H OS=Homo sapiens GN | | 0.00 | | | 4.85 | 3.38 | 1 | 1 | | 0.00 | | |
| P15924 | Desmoplakin OS=Homo s | 26.01 | 1.95 | 3 | 5 | 24.25 | 2.61 | 4 | 5 | 26.43 | 3.38 | 5 | 5 |
| P55084 | Trifunctional enzyme sub | 9.98 | 3.38 | 1 | 2 | 15.65 | 3.38 | 1 | 3 | 5.16 | 3.38 | 1 | 1 |
| O75844 | CAAX prenyl protease 1 h | 5.38 | 3.37 | 1 | 1 | 8.62 | 3.37 | 1 | 2 | 5.94 | 3.37 | 1 | 1 |
| Q96M27 | Protein PRRC1 OS=Homo | 3.89 | 3.37 | 1 | 1 | | 0.00 | | | | 0.00 | | |
| Q15833 | Syntaxin-binding protein 2 OS=Homo | | 0.00 | | | 3.98 | 3.37 | 1 | 1 | | 0.00 | | |
| Q7Z3B4 | Nucleoporin p54 OS=Homo sapiens G | | 0.00 | | | | 0.00 | | | 4.86 | 3.35 | 1 | 1 |
| Q14152 | Eukaryotic translation init | 8.16 | 2.03 | 2 | 2 | 9.75 | 2.17 | 2 | 2 | | 0.00 | | |
| Q86TD4 | Sarcalumenin OS=Homo sapiens GN= | | 0.00 | | | | 0.00 | | | 5.49 | 3.33 | 1 | 1 |
| Q96EY1 | DnaJ homolog subfamily A member 3, | | 0.00 | | | 4.46 | 3.33 | 1 | 1 | 4.82 | 3.33 | 1 | 1 |
| Q9UHI8 | A disintegrin and metallo | 5.26 | 3.31 | 1 | 1 | | 0.00 | | | | 0.00 | | |
| P78352 | Disks large homolog 4 OS=Homo sapi | | 0.00 | | | | 0.00 | | | 4.65 | 3.31 | 1 | 1 |
| Q92879 | CUGBP Elav-like family member 1 OS= | | 0.00 | | | 3.98 | 3.29 | 1 | 1 | 4.43 | 3.29 | 1 | 1 |
| Q5K4L6 | Long-chain fatty acid trar | 5.46 | 3.29 | 1 | 1 | 5.07 | 3.29 | 1 | 1 | | 0.00 | | |
| O75153 | Clustered mitochondria p | 3.87 | 1.53 | 1 | 1 | | 0.00 | | | 4.84 | 1.76 | 1 | 1 |
| P20073 | Annexin A7 OS=Homo sa | 9.60 | 3.28 | 1 | 2 | 5.32 | 3.28 | 1 | 1 | | 0.00 | | |
| P18858 | DNA ligase 1 OS=Homo s | 9.51 | 3.26 | 2 | 2 | 9.37 | 3.26 | 2 | 2 | 4.95 | 1.52 | 1 | 1 |
| P41214 | Eukaryotic translation initiation factor | | 0.00 | | | 4.13 | 3.25 | 1 | 1 | | 0.00 | | |
| P13489 | Ribonuclease inhibitor OS | 4.78 | 3.25 | 1 | 1 | | 0.00 | | | | 0.00 | | |
| Q8TEX9 | Importin-4 OS=Homo sap | 7.85 | 3.24 | 2 | 2 | 4.26 | 1.57 | 1 | 1 | 5.08 | 1.57 | 1 | 1 |
| Q2M2I5 | Keratin, type I cytoskeletal 24 OS=Ho | | 0.00 | | | | 0.00 | | | 4.44 | 3.24 | 1 | 1 |
| Q8IXI1 | Mitochondrial Rho GTPas | 5.96 | 3.24 | 1 | 1 | | 0.00 | | | | 0.00 | | |
| P49821 | NADH dehydrogenase [ul | 3.93 | 3.23 | 1 | 1 | | 0.00 | | | | 0.00 | | |
| Q1KMD3 | Heterogeneous nuclear ribonucleoprot | | 0.00 | | | 5.82 | 3.21 | 1 | 1 | 5.19 | 3.21 | 1 | 1 |
| Q9UHD9 | Ubiquilin-2 OS=Homo sap | 4.50 | 3.21 | 1 | 1 | | 0.00 | | | | 0.00 | | |
| P63010 | AP-2 complex subunit bet | 8.69 | 3.20 | 2 | 2 | | 0.00 | | | | 0.00 | | |
| Q6P1J9 | Parafibromin OS=Homo sapiens GN=( | | 0.00 | | | | 0.00 | | | 4.31 | 3.20 | 1 | 1 |
| Q6UN15 | Pre-mRNA 3'-end-processing factor FI | | 0.00 | | | 4.53 | 3.20 | 1 | 1 | 5.08 | 3.20 | 1 | 1 |
| Q53GS9 | U4/U6.U5 tri-snRNP-associated protei | | 0.00 | | | 4.38 | 3.19 | 1 | 1 | | 0.00 | | |
| P50995 | Annexin A11 OS=Homo s | 4.74 | 3.17 | 1 | 1 | | 0.00 | | | | 0.00 | | |
| O60825 | 6-phosphofructo-2-kinase | 4.39 | 3.17 | 1 | 1 | | 0.00 | | | 4.57 | 3.17 | 1 | 1 |
| Q07065 | Cytoskeleton-associated p | 4.73 | 3.16 | 1 | 1 | 4.93 | 3.16 | 1 | 1 | 5.49 | 3.16 | 1 | 1 |
| Q9H0D6 | 5'-3' exoribonuclease 2 O | 9.73 | 3.16 | 2 | 2 | 9.46 | 3.16 | 2 | 2 | 4.87 | 1.58 | 1 | 1 |
| Q10567 | AP-1 complex subunit bet | 4.83 | 1.58 | 1 | 1 | 4.07 | 1.58 | 1 | 1 | | 0.00 | | |
| O14744 | Protein arginine N-methyltransferase 5 | | 0.00 | | | 3.98 | 3.14 | 1 | 1 | 4.57 | 3.14 | 1 | 1 |
| P04004 | Vitronectin OS=Homo sap | 4.54 | 3.14 | 1 | 1 | 4.34 | 3.14 | 1 | 1 | 4.56 | 3.14 | 1 | 1 |
| Q96AE4 | Far upstream element-binding protein | | 0.00 | | | 9.65 | 3.11 | 1 | 2 | 4.56 | 3.11 | 1 | 1 |
| Q6P1M0 | Long-chain fatty acid transport protein | | 0.00 | | | | 0.00 | | | 5.44 | 3.11 | 1 | 1 |
| P56192 | Methionine--tRNA ligase, | 4.79 | 1.89 | 1 | 1 | 8.08 | 3.11 | 2 | 2 | | 0.00 | | |

| | | | | | | | | | | | | | |
|---|---|---|---|---|---|---|---|---|---|---|---|---|---|
| P49736 | DNA replication licensing factor MCM2 | | 0.00 | | | | 0.00 | | | 5.09 | 3.10 | 1 | 1 |
| Q9NP81 | Serine--tRNA ligase, mitochondrial OS | | 0.00 | | | | 0.00 | | | 5.53 | 3.09 | 1 | 1 |
| Q13867 | Bleomycin hydrolase OS=Homo sapien | | 0.00 | | | | 0.00 | | | 4.83 | 3.08 | 1 | 1 |
| P23368 | NAD-dependent malic en: | 4.29 | 3.08 | 1 | 1 | 5.00 | 3.08 | 1 | 1 | | 0.00 | | |
| Q13492 | Phosphatidylinositol-bindi | 4.42 | 3.07 | 1 | 1 | | 0.00 | | | 5.01 | 3.07 | 1 | 1 |
| Q9Y265 | RuvB-like 1 OS=Homo sa | 4.40 | 3.07 | 1 | 1 | 9.03 | 3.07 | 1 | 2 | 5.00 | 3.07 | 1 | 1 |
| P50851 | Lipopolysaccharide-respo | 9.81 | 1.96 | 2 | 2 | | 0.00 | | | 5.07 | 1.12 | 1 | 1 |
| Q9C0E8 | Protein lunapark OS=Hor | 3.85 | 3.04 | 1 | 1 | | 0.00 | | | | 0.00 | | |
| Q494U1 | Pleckstrin homology dom | 4.02 | 3.02 | 1 | 1 | | 0.00 | | | | 0.00 | | |
| Q7Z5R6 | Amyloid beta A4 precursor protein-bin | | 0.00 | | | | 0.00 | | | 4.21 | 3.00 | 1 | 1 |
| Q9HC77 | Centromere protein J OS= | 4.73 | 1.57 | 1 | 1 | | 0.00 | | | 4.24 | 1.42 | 1 | 1 |
| Q13618 | Cullin-3 OS=Homo sapiens GN=CUL3 | | 0.00 | | | | 0.00 | | | 5.23 | 2.99 | 1 | 1 |
| Q96HE7 | ERO1-like protein alpha ( | 3.87 | 2.99 | 1 | 1 | | 0.00 | | | | 0.00 | | |
| P18031 | Tyrosine-protein phospha | 4.14 | 2.99 | 1 | 1 | | 0.00 | | | | 0.00 | | |
| O15075 | Serine/threonine-protein kinase DCLK1 | | 0.00 | | | 4.25 | 2.97 | 1 | 1 | | 0.00 | | |
| P26641 | Elongation factor 1-gamn | 3.90 | 2.97 | 1 | 1 | | 0.00 | | | | 0.00 | | |
| P05166 | Propionyl-CoA carboxylas | 9.83 | 2.97 | 1 | 2 | | 0.00 | | | 4.32 | 2.97 | 1 | 1 |
| P11217 | Glycogen phosphorylase, | 5.51 | 2.97 | 1 | 1 | | 0.00 | | | | 0.00 | | |
| Q9NRY5 | Protein FAM114A2 OS=Homo sapiens | | 0.00 | | | | 0.00 | | | 4.20 | 2.97 | 1 | 1 |
| Q8WVM8 | Sec1 family domain-containing protein | | 0.00 | | | 8.98 | 2.96 | 1 | 2 | | 0.00 | | |
| Q9Y305 | Acyl-coenzyme A thioeste | 3.95 | 2.96 | 1 | 1 | | 0.00 | | | | 0.00 | | |
| P28329 | Choline O-acetyltransfera | 3.90 | 2.94 | 1 | 1 | | 0.00 | | | | 0.00 | | |
| P61201 | COP9 signalosome compl | 3.98 | 2.93 | 1 | 1 | | 0.00 | | | | 0.00 | | |
| P31930 | Cytochrome b-c1 comple: | 4.52 | 2.92 | 1 | 1 | 4.34 | 2.92 | 1 | 1 | | 0.00 | | |
| Q9UPT8 | Zinc finger CCCH domain-containing p | | 0.00 | | | 8.28 | 2.92 | 2 | 2 | | 0.00 | | |
| Q9Y4P3 | Transducin beta-like protein 2 OS=Ho | | 0.00 | | | | 0.00 | | | 4.14 | 2.91 | 1 | 1 |
| Q9HAV4 | Exportin-5 OS=Homo sap | 4.04 | 1.66 | 1 | 1 | 4.30 | 1.66 | 1 | 1 | 4.88 | 1.25 | 1 | 1 |
| P55196 | Afadin OS=Homo sapiens | 9.84 | 1.92 | 2 | 2 | 4.09 | 0.99 | 1 | 1 | 4.78 | 0.93 | 1 | 1 |
| Q7Z6Z7 | E3 ubiquitin-protein ligas | 10.30 | 0.94 | 2 | 2 | 18.62 | 1.97 | 4 | 4 | 5.49 | 0.73 | 1 | 1 |
| Q04206 | Transcription factor p65 ( | 4.65 | 2.90 | 1 | 1 | | 0.00 | | | | 0.00 | | |
| Q92973 | Transportin-1 OS=Homo sapiens GN= | | 0.00 | | | | 0.00 | | | 5.22 | 2.90 | 1 | 1 |
| Q8NCA5 | Protein FAM98A OS=Hom | 4.28 | 2.89 | 1 | 1 | 4.19 | 2.89 | 1 | 1 | | 0.00 | | |
| P05165 | Propionyl-CoA carboxylas | 4.77 | 2.88 | 1 | 1 | | 0.00 | | | | 0.00 | | |
| Q9P258 | Protein RCC2 OS=Homo sapiens GN= | | 0.00 | | | 4.79 | 2.87 | 1 | 1 | | 0.00 | | |
| Q14728 | Major facilitator superfamily domain-c | | 0.00 | | | 3.99 | 2.86 | 1 | 1 | | 0.00 | | |
| Q9NNW7 | Thioredoxin reductase 2, | 5.06 | 2.86 | 1 | 1 | 8.35 | 2.86 | 1 | 2 | 4.27 | 2.86 | 1 | 1 |
| Q9UP83 | Conserved oligomeric Gol | 5.27 | 2.86 | 1 | 1 | | 0.00 | | | | 0.00 | | |
| Q8N6L0 | Protein KASH5 OS=Homo sapiens GN= | | 0.00 | | | 4.10 | 2.85 | 1 | 1 | | 0.00 | | |
| Q9NW75 | G patch domain-containir | 4.28 | 2.84 | 1 | 1 | | 0.00 | | | | 0.00 | | |

| | | | | | | | | | | | | |
|---|---|---|---|---|---|---|---|---|---|---|---|---|
| Q03252 | Lamin-B2 OS=Homo sapiens GN=LMN | 0.00 | | | | 0.00 | | | 4.63 | 2.83 | 1 | 1 |
| Q8NF37 | Lysophosphatidylcholine acyltransfera: | 0.00 | | | 4.38 | 2.81 | 1 | 1 | | 0.00 | | |
| Q4KMQ1 | Taperin OS=Homo sapier | 4.34 | 2.53 | 1 | 1 | | 0.00 | | | 10.01 | 2.81 | 2 | 2 |
| P36915 | Guanine nucleotide-bindi | 4.66 | 2.80 | 1 | 1 | | 0.00 | | | | 0.00 | | |
| Q13573 | SNW domain-containing protein 1 OS= | 0.00 | | | | 0.00 | | | 4.32 | 2.80 | 1 | 1 |
| P52306 | Rap1 GTPase-GDP dissociation stimula | 0.00 | | | | 0.00 | | | 5.02 | 2.80 | 1 | 1 |
| Q16850 | Lanosterol 14-alpha dem | 5.12 | 2.78 | 1 | 1 | 4.82 | 2.78 | 1 | 1 | 4.31 | 2.78 | 1 | 1 |
| P01024 | Complement C3 OS=Hom | 14.38 | 2.77 | 2 | 3 | 4.89 | 1.44 | 1 | 1 | 5.42 | 1.44 | 1 | 1 |
| Q9H223 | EH domain-containing protein 4 OS=H | 0.00 | | | 4.26 | 2.77 | 1 | 1 | 4.78 | 2.77 | 1 | 1 |
| Q9UDY2 | Tight junction protein ZO | 5.80 | 2.77 | 1 | 1 | | 0.00 | | | | 0.00 | | |
| Q3KQV9 | UDP-N-acetylhexosamine | 4.20 | 2.76 | 1 | 1 | | 0.00 | | | 4.33 | 2.76 | 1 | 1 |
| A6NLF2 | RNA polymerase II transcription factor | 0.00 | | | | 0.00 | | | 4.41 | 2.75 | 1 | 1 |
| P09622 | Dihydrolipoyl dehydrogenase, mitocho | 0.00 | | | 4.03 | 2.75 | 1 | 1 | | 0.00 | | |
| Q17RY0 | Cytoplasmic polyadenylation element-l | 0.00 | | | | 0.00 | | | 4.16 | 2.74 | 1 | 1 |
| P46060 | Ran GTPase-activating pr | 5.08 | 2.73 | 1 | 1 | | 0.00 | | | 4.95 | 2.73 | 1 | 1 |
| Q9H6R4 | Nucleolar protein 6 OS=H | 5.28 | 2.71 | 1 | 1 | | 0.00 | | | | 0.00 | | |
| Q14980 | Nuclear mitotic apparatus protein 1 O: | 0.00 | | | | 0.00 | | | 14.25 | 2.70 | 3 | 3 |
| O43815 | Striatin OS=Homo sapien | 4.96 | 2.69 | 1 | 1 | | 0.00 | | | | 0.00 | | |
| E9PAV3 | Nascent polypeptide-asso | 9.15 | 1.35 | 2 | 2 | 17.22 | 2.02 | 3 | 4 | 17.45 | 2.69 | 4 | 4 |
| Q92800 | Histone-lysine N-methyltransferase EZ | 0.00 | | | 3.92 | 2.68 | 1 | 1 | | 0.00 | | |
| Q9NZB2 | Constitutive coactivator o | 8.44 | 2.68 | 2 | 2 | | 0.00 | | | | 0.00 | | |
| Q68E01 | Integrator complex subunit 3 OS=Hon | 0.00 | | | | 0.00 | | | 4.69 | 2.68 | 1 | 1 |
| P37198 | Nuclear pore glycoprotein | 4.41 | 2.68 | 1 | 1 | | 0.00 | | | | 0.00 | | |
| Q96AX1 | Vacuolar protein sorting-associated pr | 0.00 | | | 5.14 | 2.68 | 1 | 1 | | 0.00 | | |
| Q99767 | Amyloid beta A4 precursor protein-bin | 0.00 | | | 4.68 | 2.67 | 1 | 1 | 4.43 | 2.67 | 1 | 1 |
| P10909 | Clusterin OS=Homo sapie | 3.88 | 2.67 | 1 | 1 | | 0.00 | | | | 0.00 | | |
| O75027 | ATP-binding cassette sub-family B me | 0.00 | | | 4.87 | 2.66 | 1 | 1 | 4.80 | 2.66 | 1 | 1 |
| Q8IUR6 | CREB3 regulatory factor OS=Homo sa | 0.00 | | | 3.92 | 2.66 | 1 | 1 | | 0.00 | | |
| O95453 | Poly(A)-specific ribonucle | 3.84 | 2.66 | 1 | 1 | | 0.00 | | | | 0.00 | | |
| Q9H270 | Vacuolar protein sorting-a | 5.22 | 2.66 | 1 | 1 | | 0.00 | | | | 0.00 | | |
| Q6P1M3 | Lethal(2) giant larvae protein homolog | 0.00 | | | | 0.00 | | | 5.78 | 2.65 | 1 | 1 |
| O43615 | Mitochondrial import inne | 4.06 | 2.65 | 1 | 1 | | 0.00 | | | 4.18 | 2.65 | 1 | 1 |
| O00533 | Neural cell adhesion molecule L1-like | 0.00 | | | | 0.00 | | | 5.23 | 2.65 | 1 | 1 |
| Q92731 | Estrogen receptor beta OS=Homo sap | 0.00 | | | 4.05 | 2.64 | 1 | 1 | | 0.00 | | |
| P49790 | Nuclear pore complex pro | 4.00 | 1.42 | 1 | 1 | | 0.00 | | | 5.50 | 1.22 | 1 | 1 |
| Q5T0N5 | Formin-binding protein 1-like OS=Hon | 0.00 | | | | 0.00 | | | 4.27 | 2.64 | 1 | 1 |
| P02771 | Alpha-fetoprotein OS=Ho | 10.35 | 2.63 | 1 | 2 | 10.68 | 2.63 | 1 | 2 | 10.69 | 2.63 | 1 | 2 |
| O94826 | Mitochondrial import rece | 3.86 | 2.63 | 1 | 1 | | 0.00 | | | | 0.00 | | |
| Q9BSC4 | Nucleolar protein 10 OS= | 4.59 | 2.62 | 1 | 1 | | 0.00 | | | | 0.00 | | |

| | | | | | | | | | | | | | |
|---|---|---|---|---|---|---|---|---|---|---|---|---|---|
| Q14839 | Chromodomain-helicase-I | 9.76 | 1.94 | 2 | 2 | 8.26 | 1.78 | 2 | 2 | 5.52 | 1.10 | 1 | 1 |
| Q9H4G0 | Band 4.1-like protein 1 OS=Homo sap | 0.00 | | | | | 0.00 | | | 4.35 | 2.61 | 1 | 1 |
| Q70EK8 | Inactive ubiquitin carboxy | 5.24 | 2.61 | 1 | 1 | | 0.00 | | | | 0.00 | | |
| Q9Y6W5 | Wiskott-Aldrich syndrome | 4.05 | 2.61 | 1 | 1 | | 0.00 | | | 4.23 | 2.61 | 1 | 1 |
| P49419 | Alpha-aminoadipic semial | 4.30 | 2.60 | 1 | 1 | | 0.00 | | | | 0.00 | | |
| Q9UMF0 | Intercellular adhesion mo | 4.86 | 2.60 | 1 | 1 | | 0.00 | | | | 0.00 | | |
| Q9C0B0 | RING finger protein unkempt homolog | 0.00 | | | | | 0.00 | | | 4.28 | 2.59 | 1 | 1 |
| P55884 | Eukaryotic translation initiation factor | 0.00 | | | | 4.68 | 2.58 | 1 | 1 | | 0.00 | | |
| P35241 | Radixin OS=Homo sapiens GN=RDX P | 0.00 | | | | | 0.00 | | | 4.70 | 2.57 | 1 | 1 |
| Q9Y2J8 | Protein-arginine deiminas | 4.83 | 2.56 | 1 | 1 | 5.35 | 2.56 | 1 | 1 | 4.60 | 2.56 | 1 | 1 |
| Q9H6T3 | RNA polymerase II-assoc | 4.48 | 2.56 | 1 | 1 | 4.89 | 2.56 | 1 | 1 | | 0.00 | | |
| Q2TB10 | Zinc finger protein 800 OS=Homo sap | 0.00 | | | | | 0.00 | | | 4.70 | 2.56 | 1 | 1 |
| O15371 | Eukaryotic translation init | 4.40 | 2.55 | 1 | 1 | | 0.00 | | | | 0.00 | | |
| P04745 | Alpha-amylase 1 OS=Hor | 3.99 | 2.54 | 1 | 1 | | 0.00 | | | | 0.00 | | |
| Q6P995 | Protein FAM171B OS=Homo sapiens ( | 0.00 | | | | 5.18 | 2.54 | 1 | 1 | | 0.00 | | |
| Q9P265 | Disco-interacting protein | 4.01 | 1.14 | 1 | 1 | 3.93 | 1.40 | 1 | 1 | 4.41 | 1.40 | 1 | 1 |
| Q9UFB7 | Zinc finger and BTB domain-containing | 0.00 | | | | 4.02 | 2.54 | 1 | 1 | | 0.00 | | |
| O60488 | Long-chain-fatty-acid--Co | 4.42 | 2.53 | 1 | 1 | 4.07 | 2.53 | 1 | 1 | | 0.00 | | |
| O76094 | Signal recognition particle subunit SRP | 0.00 | | | | 9.34 | 2.53 | 1 | 2 | 4.85 | 2.53 | 1 | 1 |
| P08243 | Asparagine synthetase [glutamine-hyd | 0.00 | | | | | 0.00 | | | 4.24 | 2.50 | 1 | 1 |
| Q08379 | Golgin subfamily A memb | 5.03 | 2.50 | 1 | 1 | | 0.00 | | | | 0.00 | | |
| Q9UKW4 | Guanine nucleotide exchange factor V | 0.00 | | | | | 0.00 | | | 4.29 | 2.48 | 1 | 1 |
| P04843 | Dolichyl-diphosphooligosaccharide--pr | 0.00 | | | | 4.11 | 2.47 | 1 | 1 | | 0.00 | | |
| P02461 | Collagen alpha-1(III) chain OS=Homo | 0.00 | | | | 4.70 | 2.46 | 1 | 1 | | 0.00 | | |
| Q13219 | Pappalysin-1 OS=Homo s | 5.31 | 2.46 | 1 | 1 | | 0.00 | | | | 0.00 | | |
| O95831 | Apoptosis-inducing factor 1, mitochon | 0.00 | | | | | 0.00 | | | 4.22 | 2.45 | 1 | 1 |
| P08237 | ATP-dependent 6-phosph | 8.84 | 2.44 | 1 | 2 | | 0.00 | | | 4.15 | 2.44 | 1 | 1 |
| O95644 | Nuclear factor of activate | 4.73 | 2.44 | 1 | 1 | | 0.00 | | | | 0.00 | | |
| O75694 | Nuclear pore complex pro | 8.59 | 2.44 | 2 | 2 | 4.27 | 1.22 | 1 | 1 | 4.73 | 1.22 | 1 | 1 |
| Q99615 | DnaJ homolog subfamily C member 7 | 0.00 | | | | | 0.00 | | | 4.19 | 2.43 | 1 | 1 |
| Q9H845 | Acyl-CoA dehydrogenase family memb | 0.00 | | | | 4.26 | 2.42 | 1 | 1 | 4.47 | 2.42 | 1 | 1 |
| P10155 | 60 kDa SS-A/Ro ribonucle | 4.09 | 2.42 | 1 | 1 | | 0.00 | | | | 0.00 | | |
| Q8NCL4 | Polypeptide N-acetylgalactosaminyltra | 0.00 | | | | | 0.00 | | | 4.97 | 2.41 | 1 | 1 |
| Q4G0J3 | La-related protein 7 OS= | 3.97 | 2.41 | 1 | 1 | | 0.00 | | | | 0.00 | | |
| Q6P996 | Pyridoxal-dependent deca | 5.75 | 2.41 | 1 | 1 | 5.54 | 2.41 | 1 | 1 | 5.61 | 2.41 | 1 | 1 |
| P49589 | Cysteine--tRNA ligase, cytoplasmic OS | 0.00 | | | | | 0.00 | | | 4.78 | 2.41 | 1 | 1 |
| Q9GZY0 | Nuclear RNA export facto | 4.23 | 2.40 | 1 | 1 | | 0.00 | | | | 0.00 | | |
| Q9Y3Z3 | Deoxynucleoside triphosp | 4.73 | 2.40 | 1 | 1 | 4.09 | 2.40 | 1 | 1 | | 0.00 | | |
| Q15063 | Periostin OS=Homo sapie | 10.61 | 2.39 | 1 | 2 | 4.34 | 2.39 | 1 | 1 | | 0.00 | | |

| | | | | | | | | | | | | | |
|---|---|---|---|---|---|---|---|---|---|---|---|---|---|
| A6NFQ2 | Protein FAM115C OS=Homo sapiens ( | | 0.00 | | | | 0.00 | | | 4.13 | 2.39 | 1 | 1 |
| Q15849 | Urea transporter 2 OS=Homo sapiens | | 0.00 | | | | 0.00 | | | 4.64 | 2.39 | 1 | 1 |
| Q12797 | Aspartyl/asparaginyl beta | 5.19 | 2.37 | 1 | 1 | | 0.00 | | | 5.70 | 2.37 | 1 | 1 |
| P06400 | Retinoblastoma-associated protein OS | | 0.00 | | | | 0.00 | | | 4.27 | 2.37 | 1 | 1 |
| P41743 | Protein kinase C iota type OS=Homo s | | 0.00 | | | | 0.00 | | | 4.10 | 2.35 | 1 | 1 |
| Q9UJC3 | Protein Hook homolog 1 ( | 4.47 | 2.34 | 1 | 1 | | 0.00 | | | 4.94 | 2.34 | 1 | 1 |
| Q7L804 | Rab11 family-interacting protein 2 OS | | 0.00 | | | 4.34 | 2.34 | 1 | 1 | 4.32 | 2.34 | 1 | 1 |
| Q9UBT2 | SUMO-activating enzyme | 4.10 | 2.34 | 1 | 1 | | 0.00 | | | | 0.00 | | |
| Q86Y56 | Dynein assembly factor 5, axonemal C | | 0.00 | | | 5.34 | 2.34 | 1 | 1 | 5.75 | 2.34 | 1 | 1 |
| Q9NY61 | Protein AATF OS=Homo s | 4.41 | 2.32 | 1 | 1 | | 0.00 | | | 4.25 | 2.32 | 1 | 1 |
| Q96ST2 | Protein IWS1 homolog OS | 4.50 | 2.32 | 1 | 1 | | 0.00 | | | | 0.00 | | |
| Q96EY7 | Pentatricopeptide repeat | 4.70 | 2.32 | 1 | 1 | 10.53 | 2.32 | 1 | 2 | | 0.00 | | |
| Q6P4Q7 | Metal transporter CNNM4 OS=Homo s | | 0.00 | | | | 0.00 | | | 4.92 | 2.32 | 1 | 1 |
| Q9NQX3 | Gephyrin OS=Homo sapie | 3.91 | 2.31 | 1 | 1 | | 0.00 | | | 4.63 | 2.31 | 1 | 1 |
| O43264 | Centromere/kinetochore p | 4.80 | 2.31 | 1 | 1 | | 0.00 | | | 4.20 | 2.31 | 1 | 1 |
| A6NDK9 | Putative golgin subfamily A member 6 | | 0.00 | | | | 0.00 | | | 4.15 | 2.31 | 1 | 1 |
| Q9H0M4 | Zinc finger CW-type PWWP domain pr | | 0.00 | | | | 0.00 | | | 4.67 | 2.31 | 1 | 1 |
| Q05209 | Tyrosine-protein phospha | 4.31 | 2.31 | 1 | 1 | 4.40 | 2.31 | 1 | 1 | | 0.00 | | |
| Q92615 | La-related protein 4B OS=Homo sapie | | 0.00 | | | | 0.00 | | | 4.33 | 2.30 | 1 | 1 |
| Q9NVC6 | Mediator of RNA polymerase II transc | | 0.00 | | | 4.23 | 2.30 | 1 | 1 | | 0.00 | | |
| Q06210 | Glutamine--fructose-6-phosphate amir | | 0.00 | | | 4.62 | 2.29 | 1 | 1 | 5.05 | 2.29 | 1 | 1 |
| Q8IVT5 | Kinase suppressor of Ras 1 OS=Homo | | 0.00 | | | | 0.00 | | | 4.61 | 2.28 | 1 | 1 |
| O00139 | Kinesin-like protein KIF2A OS=Homo s | | 0.00 | | | | 0.00 | | | 4.44 | 2.27 | 1 | 1 |
| O95359 | Transforming acidic coilec | 9.36 | 1.32 | 2 | 2 | 9.34 | 1.56 | 2 | 2 | 4.55 | 0.61 | 1 | 1 |
| Q8NBJ5 | Procollagen galactosyltransferase 1 OS | | 0.00 | | | | 0.00 | | | 8.25 | 2.25 | 1 | 2 |
| O43719 | HIV Tat-specific factor 1 OS=Homo sa | | 0.00 | | | | 0.00 | | | 4.63 | 2.25 | 1 | 1 |
| O43290 | U4/U6.U5 tri-snRNP-associated protei | | 0.00 | | | | 0.00 | | | 5.33 | 2.25 | 1 | 1 |
| Q9UBY0 | Sodium/hydrogen exchanger 2 OS=Hc | | 0.00 | | | 4.07 | 2.22 | 1 | 1 | | 0.00 | | |
| Q93084 | Sarcoplasmic/endoplasmic reticulum c | | 0.00 | | | | 0.00 | | | 5.90 | 2.21 | 1 | 1 |
| P52701 | DNA mismatch repair protein Msh6 OS | | 0.00 | | | 8.92 | 2.21 | 2 | 2 | 4.28 | 0.88 | 1 | 1 |
| P50416 | Carnitine O-palmitoyltran | 5.08 | 2.20 | 1 | 1 | 5.93 | 2.20 | 1 | 1 | 5.52 | 2.20 | 1 | 1 |
| Q5T7W0 | Zinc finger protein 618 OS=Homo sap | | 0.00 | | | | 0.00 | | | 4.12 | 2.20 | 1 | 1 |
| P49023 | Paxillin OS=Homo sapien | 4.46 | 2.20 | 1 | 1 | | 0.00 | | | | 0.00 | | |
| P33402 | Guanylate cyclase soluble subunit alph | | 0.00 | | | | 0.00 | | | 4.13 | 2.19 | 1 | 1 |
| Q16822 | Phosphoenolpyruvate car | 3.98 | 2.19 | 1 | 1 | | 0.00 | | | | 0.00 | | |
| Q92621 | Nuclear pore complex protein Nup205 | | 0.00 | | | 5.27 | 1.39 | 1 | 1 | 4.30 | 0.80 | 1 | 1 |
| Q6ZVT6 | Uncharacterized protein C3orf67 OS=I | | 0.00 | | | 3.94 | 2.18 | 1 | 1 | | 0.00 | | |
| P84550 | SKI family transcriptional corepressor | | 0.00 | | | | 0.00 | | | 4.10 | 2.18 | 1 | 1 |
| Q6PJT7 | Zinc finger CCCH domain-containing p | | 0.00 | | | | 0.00 | | | 4.18 | 2.17 | 1 | 1 |

| | | | | | | | | | | | | | |
|---|---|---|---|---|---|---|---|---|---|---|---|---|---|
| Q9Y592 | Centrosomal protein of 83 kDa OS=Ho | | 0.00 | | | | 0.00 | | | 4.10 | 2.16 | 1 | 1 |
| Q96KP1 | Exocyst complex component 2 OS=Ho | | 0.00 | | | 4.57 | 2.16 | 1 | 1 | | 0.00 | | |
| O75382 | Tripartite motif-containin | 4.30 | 2.15 | 1 | 1 | | 0.00 | | | | 0.00 | | |
| P13671 | Complement component C6 OS=Hom | | 0.00 | | | 3.93 | 2.14 | 1 | 1 | | 0.00 | | |
| Q8IXB1 | DnaJ homolog subfamily C member 10 | | 0.00 | | | 4.11 | 2.14 | 1 | 1 | | 0.00 | | |
| Q13214 | Semaphorin-3B OS=Homo sapiens GN | | 0.00 | | | 3.96 | 2.14 | 1 | 1 | | 0.00 | | |
| Q5T4S7 | E3 ubiquitin-protein ligas | 9.74 | 1.00 | 2 | 2 | 4.82 | 0.56 | 1 | 1 | 5.22 | 0.58 | 1 | 1 |
| P43243 | Matrin-3 OS=Homo sapiens GN=MATI | | 0.00 | | | 4.46 | 2.13 | 1 | 1 | | 0.00 | | |
| P23229 | Integrin alpha-6 OS=Homo sapiens GI | | 0.00 | | | | 0.00 | | | 5.17 | 2.12 | 1 | 1 |
| Q14008 | Cytoskeleton-associated p | 4.24 | 0.69 | 1 | 1 | | 0.00 | | | 5.19 | 1.43 | 1 | 1 |
| P02751 | Fibronectin OS=Homo sa | 9.67 | 1.38 | 2 | 2 | 4.45 | 0.71 | 1 | 1 | | 0.00 | | |
| P17480 | Nucleolar transcription factor 1 OS=Ho | | 0.00 | | | | 0.00 | | | 4.50 | 2.09 | 1 | 1 |
| Q86Y38 | Xylosyltransferase 1 OS= | 4.09 | 2.09 | 1 | 1 | | 0.00 | | | | 0.00 | | |
| P54577 | Tyrosine--tRNA ligase, cy | 3.94 | 2.08 | 1 | 1 | | 0.00 | | | | 0.00 | | |
| Q9NQI0 | Probable ATP-dependent RNA helicase | | 0.00 | | | | 0.00 | | | 4.31 | 2.07 | 1 | 1 |
| Q9NZT2 | Opioid growth factor rece | 3.86 | 2.07 | 1 | 1 | | 0.00 | | | | 0.00 | | |
| O75182 | Paired amphipathic helix | 4.85 | 2.07 | 1 | 1 | | 0.00 | | | | 0.00 | | |
| Q14258 | E3 ubiquitin/ISG15 ligase | 4.61 | 2.06 | 1 | 1 | | 0.00 | | | | 0.00 | | |
| Q9H7Z3 | Protein NRDE2 homolog OS=Homo sa | | 0.00 | | | 4.72 | 2.06 | 1 | 1 | | 0.00 | | |
| Q8IYB8 | ATP-dependent RNA helicase SUPV3L1 | | 0.00 | | | 4.16 | 2.04 | 1 | 1 | | 0.00 | | |
| O60242 | Brain-specific angiogenesis inhibitor 3 | | 0.00 | | | 5.19 | 2.04 | 1 | 1 | | 0.00 | | |
| Q6PL18 | ATPase family AAA domain-containing | | 0.00 | | | 9.10 | 2.01 | 2 | 2 | 4.61 | 1.08 | 1 | 1 |
| Q8IW93 | Rho guanine nucleotide exchange fact | | 0.00 | | | 4.05 | 2.00 | 1 | 1 | | 0.00 | | |
| Q99459 | Cell division cycle 5-like p | 4.07 | 2.00 | 1 | 1 | | 0.00 | | | | 0.00 | | |
| Q8IZ83 | Aldehyde dehydrogenase family 16 me | | 0.00 | | | | 0.00 | | | 4.91 | 2.00 | 1 | 1 |
| Q9Y6T7 | Diacylglycerol kinase beta | 4.11 | 1.99 | 1 | 1 | | 0.00 | | | | 0.00 | | |
| Q86YT6 | E3 ubiquitin-protein ligase MIB1 OS=H | | 0.00 | | | 4.17 | 1.99 | 1 | 1 | | 0.00 | | |
| Q6WCQ1 | Myosin phosphatase Rho- | 3.88 | 1.95 | 1 | 1 | | 0.00 | | | | 0.00 | | |
| P52735 | Guanine nucleotide exchange factor V | | 0.00 | | | | 0.00 | | | 4.48 | 1.94 | 1 | 1 |
| P35658 | Nuclear pore complex pro | 4.11 | 1.05 | 1 | 1 | 4.03 | 1.05 | 1 | 1 | 5.09 | 0.86 | 1 | 1 |
| P35221 | Catenin alpha-1 OS=Hom | 4.36 | 1.88 | 1 | 1 | | 0.00 | | | | 0.00 | | |
| Q8IZL8 | Proline-, glutamic acid- ar | 4.34 | 1.86 | 1 | 1 | 4.00 | 1.86 | 1 | 1 | | 0.00 | | |
| P12109 | Collagen alpha-1(VI) chai | 5.32 | 1.85 | 1 | 1 | 10.87 | 1.85 | 1 | 2 | 11.52 | 1.85 | 1 | 2 |
| O75152 | Zinc finger CCCH domain-containing p | | 0.00 | | | 4.13 | 1.85 | 1 | 1 | | 0.00 | | |
| P57740 | Nuclear pore complex pro | 4.98 | 1.84 | 1 | 1 | | 0.00 | | | | 0.00 | | |
| P28290 | Sperm-specific antigen 2 | 4.02 | 1.83 | 1 | 1 | | 0.00 | | | | 0.00 | | |
| P07384 | Calpain-1 catalytic subuni | 4.02 | 1.82 | 1 | 1 | | 0.00 | | | | 0.00 | | |
| P55011 | Solute carrier family 12 member 2 OS- | | 0.00 | | | | 0.00 | | | 4.30 | 1.82 | 1 | 1 |
| Q86XP3 | ATP-dependent RNA helic | 3.90 | 1.81 | 1 | 1 | | 0.00 | | | | 0.00 | | |

| | | | | | | | | | | | | | |
|---|---|---|---|---|---|---|---|---|---|---|---|---|---|
| Q8TCU6 | Phosphatidylinositol 3,4,5 | 4.84 | 1.81 | 1 | 1 | | 0.00 | | | | 0.00 | | |
| O94906 | Pre-mRNA-processing factor 6 OS=Ho | | 0.00 | | | 4.85 | 1.81 | 1 | 1 | | 0.00 | | |
| P52732 | Kinesin-like protein KIF11 OS=Homo s | | 0.00 | | | | 0.00 | | | 4.35 | 1.80 | 1 | 1 |
| P17844 | Probable ATP-dependent RNA helicase | | 0.00 | | | | 0.00 | | | 4.11 | 1.79 | 1 | 1 |
| Q96RQ3 | Methylcrotonoyl-CoA carb | 4.65 | 1.79 | 1 | 1 | 4.26 | 1.79 | 1 | 1 | 4.40 | 1.79 | 1 | 1 |
| Q7Z410 | Transmembrane protease serine 9 OS | | 0.00 | | | | 0.00 | | | 4.29 | 1.79 | 1 | 1 |
| Q658Y4 | Protein FAM91A1 OS=Homo sapiens ( | | 0.00 | | | 4.60 | 1.79 | 1 | 1 | | 0.00 | | |
| Q6VY07 | Phosphofurin acidic cluster sorting pro | | 0.00 | | | | 0.00 | | | 4.38 | 1.77 | 1 | 1 |
| Q5JTH9 | RRP12-like protein OS=Homo sapiens | | 0.00 | | | | 0.00 | | | 5.55 | 1.77 | 1 | 1 |
| Q9NSV4 | Protein diaphanous homo | 3.86 | 1.76 | 1 | 1 | | 0.00 | | | | 0.00 | | |
| Q13563 | Polycystin-2 OS=Homo sa | 4.22 | 1.76 | 1 | 1 | | 0.00 | | | | 0.00 | | |
| Q15003 | Condensin complex subunit 2 OS=Hor | | 0.00 | | | | 0.00 | | | 4.70 | 1.75 | 1 | 1 |
| Q99613 | Eukaryotic translation init | 5.49 | 1.75 | 1 | 1 | 8.72 | 1.75 | 1 | 2 | 5.54 | 1.75 | 1 | 1 |
| Q9BUJ2 | Heterogeneous nuclear ri | 5.37 | 1.75 | 1 | 1 | 4.76 | 1.75 | 1 | 1 | | 0.00 | | |
| O15381 | Nuclear valosin-containin | 3.93 | 1.75 | 1 | 1 | 4.50 | 1.75 | 1 | 1 | | 0.00 | | |
| P46459 | Vesicle-fusing ATPase OS | 4.77 | 1.75 | 1 | 1 | 4.37 | 1.75 | 1 | 1 | | 0.00 | | |
| P52789 | Hexokinase-2 OS=Homo sapiens GN= | | 0.00 | | | 5.38 | 1.74 | 1 | 1 | | 0.00 | | |
| Q96P70 | Importin-9 OS=Homo sap | 5.16 | 1.73 | 1 | 1 | 4.75 | 1.73 | 1 | 1 | 5.59 | 1.73 | 1 | 1 |
| Q9H2P0 | Activity-dependent neuro | 3.90 | 1.72 | 1 | 1 | | 0.00 | | | 4.59 | 1.72 | 1 | 1 |
| Q6P3W7 | SCY1-like protein 2 OS=H | 3.85 | 1.72 | 1 | 1 | | 0.00 | | | | 0.00 | | |
| P49747 | Cartilage oligomeric matr | 3.95 | 1.72 | 1 | 1 | | 0.00 | | | | 0.00 | | |
| Q6NSJ2 | Pleckstrin homology-like ( | 3.84 | 1.72 | 1 | 1 | | 0.00 | | | | 0.00 | | |
| Q01804 | OTU domain-containing p | 3.99 | 1.71 | 1 | 1 | | 0.00 | | | | 0.00 | | |
| Q9Y4F5 | Centrosomal protein of 1 | 4.94 | 1.70 | 1 | 1 | | 0.00 | | | | 0.00 | | |
| Q6Y7W6 | PERQ amino acid-rich with GYF domai | | 0.00 | | | | 0.00 | | | 5.23 | 1.69 | 1 | 1 |
| Q8IX07 | Zinc finger protein ZFPM1 OS=Homo s | | 0.00 | | | | 0.00 | | | 4.15 | 1.69 | 1 | 1 |
| Q5JTC6 | APC membrane recruitment protein 1 | | 0.00 | | | 3.93 | 1.67 | 1 | 1 | | 0.00 | | |
| P78524 | Suppression of tumoriger | 3.82 | 1.67 | 1 | 1 | | 0.00 | | | | 0.00 | | |
| Q9NYA4 | Myotubularin-related protein 4 OS=Ho | | 0.00 | | | 3.98 | 1.67 | 1 | 1 | | 0.00 | | |
| Q13200 | 26S proteasome non-ATPase regulator | | 0.00 | | | 4.05 | 1.65 | 1 | 1 | 5.18 | 1.65 | 1 | 1 |
| P53992 | Protein transport protein | 4.17 | 1.65 | 1 | 1 | | 0.00 | | | | 0.00 | | |
| O95373 | Importin-7 OS=Homo sapiens GN=IP( | | 0.00 | | | | 0.00 | | | 5.08 | 1.64 | 1 | 1 |
| Q15459 | Splicing factor 3A subunit 1 OS=Homo | | 0.00 | | | | 0.00 | | | 4.42 | 1.64 | 1 | 1 |
| Q6UB35 | Monofunctional C1-tetrahydrofolate sy | | 0.00 | | | | 0.00 | | | 4.28 | 1.64 | 1 | 1 |
| A0FGR8 | Extended synaptotagmin- | 4.16 | 1.63 | 1 | 1 | | 0.00 | | | | 0.00 | | |
| Q14676 | Mediator of DNA damage checkpoint p | | 0.00 | | | 4.74 | 1.63 | 1 | 1 | | 0.00 | | |
| Q9Y4E8 | Ubiquitin carboxyl-termin | 4.26 | 1.63 | 1 | 1 | | 0.00 | | | | 0.00 | | |
| O15020 | Spectrin beta chain, non- | 4.45 | 0.84 | 1 | 1 | 5.11 | 0.84 | 1 | 1 | 4.59 | 0.79 | 1 | 1 |
| Q9Y6D5 | Brefeldin A-inhibited guar | 8.16 | 1.62 | 2 | 2 | | 0.00 | | | 5.27 | 0.84 | 1 | 1 |

| | | | | | | | | | | | | | |
|---|---|---|---|---|---|---|---|---|---|---|---|---|---|
| Q12788 | Transducin beta-like protein 3 OS=Ho | | 0.00 | | | | 0.00 | | | 4.54 | 1.61 | 1 | 1 |
| Q9NQW6 | Actin-binding protein anillin OS=Homo | | 0.00 | | | | 0.00 | | | 4.53 | 1.60 | 1 | 1 |
| Q5SNT6 | WASH complex subunit FAM21B OS=H | | 0.00 | | | | 0.00 | | | 5.03 | 1.60 | 1 | 1 |
| Q9Y3P9 | Rab GTPase-activating pr | 4.36 | 1.59 | 1 | 1 | | 0.00 | | | | 0.00 | | |
| Q9NPG3 | Ubinuclein-1 OS=Homo sapiens GN=U | | 0.00 | | | | 0.00 | | | 4.15 | 1.59 | 1 | 1 |
| Q9H3U1 | Protein unc-45 homolog A | 3.88 | 1.59 | 1 | 1 | | 0.00 | | | | 0.00 | | |
| Q5JRA6 | Melanoma inhibitory activ | 4.99 | 0.89 | 1 | 1 | 4.10 | 0.68 | 1 | 1 | | 0.00 | | |
| Q9BZH6 | WD repeat-containing protein 11 OS= | | 0.00 | | | | 0.00 | | | 5.42 | 1.55 | 1 | 1 |
| Q6PKG0 | La-related protein 1 OS= | 4.99 | 1.55 | 1 | 1 | | 0.00 | | | 5.05 | 1.55 | 1 | 1 |
| Q9BYX2 | TBC1 domain family member 2A OS=I | | 0.00 | | | | 0.00 | | | 4.31 | 1.51 | 1 | 1 |
| Q9UPN9 | E3 ubiquitin-protein ligase TRIM33 OS | | 0.00 | | | 4.13 | 1.51 | 1 | 1 | | 0.00 | | |
| Q29RF7 | Sister chromatid cohesion protein PDS | | 0.00 | | | 4.16 | 1.50 | 1 | 1 | 10.43 | 1.50 | 1 | 2 |
| Q14324 | Myosin-binding protein C, fast-type OS | | 0.00 | | | 3.91 | 1.49 | 1 | 1 | | 0.00 | | |
| Q8IX12 | Cell division cycle and apo | 3.98 | 1.48 | 1 | 1 | | 0.00 | | | | 0.00 | | |
| Q9UHF7 | Zinc finger transcription f | 5.26 | 1.48 | 1 | 1 | | 0.00 | | | 4.74 | 1.48 | 1 | 1 |
| P27816 | Microtubule-associated pr | 3.95 | 1.48 | 1 | 1 | 3.95 | 1.48 | 1 | 1 | | 0.00 | | |
| Q8N1F7 | Nuclear pore complex protein Nup93 O | | 0.00 | | | 4.22 | 1.47 | 1 | 1 | | 0.00 | | |
| P36776 | Lon protease homolog, m | 4.18 | 1.46 | 1 | 1 | | 0.00 | | | | 0.00 | | |
| O14974 | Protein phosphatase 1 re | 4.78 | 1.46 | 1 | 1 | | 0.00 | | | | 0.00 | | |
| O43592 | Exportin-T OS=Homo sap | 3.88 | 1.46 | 1 | 1 | | 0.00 | | | | 0.00 | | |
| Q9BSJ8 | Extended synaptotagmin- | 4.88 | 1.45 | 1 | 1 | | 0.00 | | | 5.29 | 1.45 | 1 | 1 |
| P30876 | DNA-directed RNA polymerase II subu | | 0.00 | | | | 0.00 | | | 4.65 | 1.45 | 1 | 1 |
| P49588 | Alanine--tRNA ligase, cytoplasmic OS= | | 0.00 | | | 4.01 | 1.45 | 1 | 1 | | 0.00 | | |
| P11498 | Pyruvate carboxylase, mit | 4.86 | 1.44 | 1 | 1 | | 0.00 | | | | 0.00 | | |
| Q86VH2 | Kinesin-like protein KIF27 OS=Homo s | | 0.00 | | | | 0.00 | | | 4.54 | 1.43 | 1 | 1 |
| O60264 | SWI/SNF-related matrix-associated ac | | 0.00 | | | 4.10 | 1.43 | 1 | 1 | 4.15 | 1.43 | 1 | 1 |
| Q8TDI7 | Transmembrane channel-like protein 2 | | 0.00 | | | | 0.00 | | | 4.29 | 1.43 | 1 | 1 |
| P82279 | Protein crumbs homolog | 4.00 | 1.42 | 1 | 1 | | 0.00 | | | | 0.00 | | |
| O15067 | Phosphoribosylformylglyc | 4.11 | 1.42 | 1 | 1 | | 0.00 | | | | 0.00 | | |
| O60610 | Protein diaphanous homo | 4.99 | 1.42 | 1 | 1 | 10.47 | 1.42 | 1 | 2 | | 0.00 | | |
| Q6ZTY8 | Putative uncharacterized protein C12o | | 0.00 | | | 4.23 | 1.42 | 1 | 1 | | 0.00 | | |
| Q96KR1 | Zinc finger RNA-binding protein OS=H | | 0.00 | | | | 0.00 | | | 4.15 | 1.40 | 1 | 1 |
| P49916 | DNA ligase 3 OS=Homo s | 4.41 | 1.39 | 1 | 1 | | 0.00 | | | | 0.00 | | |
| P69849 | Nodal modulator 3 OS=Homo sapiens | | 0.00 | | | | 0.00 | | | 4.49 | 1.39 | 1 | 1 |
| Q9NTI5 | Sister chromatid cohesion protein PDS | | 0.00 | | | | 0.00 | | | 4.36 | 1.38 | 1 | 1 |
| Q13009 | T-lymphoma invasion and | 3.98 | 1.38 | 1 | 1 | | 0.00 | | | | 0.00 | | |
| Q9Y2L1 | Exosome complex exonuclease RRP44 | | 0.00 | | | 4.06 | 1.36 | 1 | 1 | 4.37 | 1.36 | 1 | 1 |
| P49746 | Thrombospondin-3 OS=H | 3.91 | 1.36 | 1 | 1 | | 0.00 | | | | 0.00 | | |
| Q15021 | Condensin complex subunit 1 OS=Hon | | 0.00 | | | | 0.00 | | | 4.35 | 1.36 | 1 | 1 |

| | | | | | | | | | | | | | |
|---|---|---|---|---|---|---|---|---|---|---|---|---|---|
| Q9P217 | Zinc finger SWIM domain-containing p | | 0.00 | | | | 0.00 | | | 4.19 | 1.35 | 1 | 1 |
| Q5JV73 | FERM and PDZ domain-containing pro | | 0.00 | | | 5.15 | 1.33 | 1 | 1 | | 0.00 | | |
| P51610 | Host cell factor 1 OS=Homo sapiens G | | 0.00 | | | | 0.00 | | | 5.52 | 1.33 | 1 | 1 |
| Q9UGU0 | Transcription factor 20 O: | 4.04 | 1.33 | 1 | 1 | | 0.00 | | | | 0.00 | | |
| O15084 | Serine/threonine-protein phosphatase | | 0.00 | | | 4.08 | 1.33 | 1 | 1 | | 0.00 | | |
| P08183 | Multidrug resistance protein 1 OS=Ho | | 0.00 | | | 4.22 | 1.33 | 1 | 1 | | 0.00 | | |
| Q5T1H1 | Protein eyes shut homolo | 4.90 | 0.76 | 1 | 1 | 4.00 | 0.57 | 1 | 1 | | 0.00 | | |
| P19827 | Inter-alpha-trypsin inhibit | 3.93 | 1.32 | 1 | 1 | | 0.00 | | | | 0.00 | | |
| P20908 | Collagen alpha-1(V) chair | 3.88 | 1.31 | 1 | 1 | | 0.00 | | | | 0.00 | | |
| Q92614 | Unconventional myosin-X | 4.05 | 0.73 | 1 | 1 | 3.93 | 0.58 | 1 | 1 | | 0.00 | | |
| Q96J66 | ATP-binding cassette sub | 3.90 | 1.30 | 1 | 1 | | 0.00 | | | | 0.00 | | |
| O95239 | Chromosome-associated kinesin KIF4A | | 0.00 | | | 4.01 | 1.30 | 1 | 1 | | 0.00 | | |
| P22102 | Trifunctional purine biosy | 5.07 | 1.29 | 1 | 1 | 4.70 | 1.29 | 1 | 1 | | 0.00 | | |
| O60292 | Signal-induced proliferati | 4.05 | 1.29 | 1 | 1 | | 0.00 | | | | 0.00 | | |
| Q7Z3K3 | Pogo transposable element with ZNF c | | 0.00 | | | | 0.00 | | | 4.83 | 1.28 | 1 | 1 |
| Q93009 | Ubiquitin carboxyl-terminal hydrolase | | 0.00 | | | | 0.00 | | | 4.20 | 1.27 | 1 | 1 |
| Q08211 | ATP-dependent RNA helic | 3.87 | 1.26 | 1 | 1 | | 0.00 | | | 4.70 | 1.26 | 1 | 1 |
| Q14126 | Desmoglein-2 OS=Homo sapiens GN= | | 0.00 | | | 4.37 | 1.25 | 1 | 1 | 4.97 | 1.25 | 1 | 1 |
| Q96KQ7 | Histone-lysine N-methyltr | 3.95 | 1.24 | 1 | 1 | | 0.00 | | | | 0.00 | | |
| A0AVT1 | Ubiquitin-like modifier-activating enzy | | 0.00 | | | 3.97 | 1.24 | 1 | 1 | | 0.00 | | |
| Q92616 | Translational activator GC | 8.86 | 1.24 | 2 | 2 | 4.32 | 0.60 | 1 | 1 | 4.35 | 0.64 | 1 | 1 |
| Q9C0C9 | E2/E3 hybrid ubiquitin-pr | 4.91 | 1.24 | 1 | 1 | | 0.00 | | | | 0.00 | | |
| Q8IZH2 | 5'-3' exoribonuclease 1 OS=Homo sap | | 0.00 | | | | 0.00 | | | 5.01 | 1.23 | 1 | 1 |
| Q99700 | Ataxin-2 OS=Homo sapie | 3.82 | 1.22 | 1 | 1 | | 0.00 | | | | 0.00 | | |
| Q9Y4F4 | Protein FAM179B OS=Ho | 4.97 | 1.22 | 1 | 1 | | 0.00 | | | | 0.00 | | |
| O60271 | C-Jun-amino-terminal kin | 3.89 | 1.21 | 1 | 1 | | 0.00 | | | | 0.00 | | |
| O60486 | Plexin-C1 OS=Homo sapi | 3.88 | 1.21 | 1 | 1 | | 0.00 | | | | 0.00 | | |
| Q08378 | Golgin subfamily A memb | 4.51 | 1.20 | 1 | 1 | | 0.00 | | | | 0.00 | | |
| Q8TEQ6 | Gem-associated protein 5 | 3.99 | 1.19 | 1 | 1 | 4.02 | 1.19 | 1 | 1 | 4.59 | 1.19 | 1 | 1 |
| Q10570 | Cleavage and polyadenyla | 4.50 | 1.18 | 1 | 1 | | 0.00 | | | | 0.00 | | |
| Q9UM54 | Unconventional myosin-V | 4.72 | 1.16 | 1 | 1 | | 0.00 | | | | 0.00 | | |
| P12107 | Collagen alpha-1(XI) chain OS=Homo | | 0.00 | | | 8.24 | 1.16 | 1 | 2 | | 0.00 | | |
| P46379 | Large proline-rich protein BAG6 OS=H | | 0.00 | | | | 0.00 | | | 4.55 | 1.15 | 1 | 1 |
| P41229 | Lysine-specific demethyla | 3.85 | 1.15 | 1 | 1 | | 0.00 | | | | 0.00 | | |
| O60313 | Dynamin-like 120 kDa protein, mitoch | | 0.00 | | | 3.98 | 1.15 | 1 | 1 | | 0.00 | | |
| O60841 | Eukaryotic translation initiation factor | | 0.00 | | | | 0.00 | | | 9.48 | 1.15 | 1 | 2 |
| P25940 | Collagen alpha-3(V) chain OS=Homo s | | 0.00 | | | 4.03 | 1.15 | 1 | 1 | | 0.00 | | |
| Q15648 | Mediator of RNA polymer | 4.10 | 1.14 | 1 | 1 | | 0.00 | | | | 0.00 | | |
| Q9Y4C1 | Lysine-specific demethyla | 3.81 | 1.14 | 1 | 1 | | 0.00 | | | | 0.00 | | |

| | | | | | | | | | | | | | |
|---|---|---|---|---|---|---|---|---|---|---|---|---|---|
| Q7L576 | Cytoplasmic FMR1-interac | 3.93 | 1.12 | 1 | 1 | | 0.00 | | | | 0.00 | | |
| Q9C0I4 | Thrombospondin type-1 c | 3.83 | 1.12 | 1 | 1 | | 0.00 | | | | 0.00 | | |
| P26358 | DNA (cytosine-5)-methylt | 4.56 | 1.11 | 1 | 1 | | 0.00 | | | | 0.00 | | |
| Q8WWZ4 | ATP-binding cassette sub-family A me | 0.00 | | | | | 0.00 | | | 4.14 | 1.10 | 1 | 1 |
| Q8IZD2 | Histone-lysine N-methyltransferase 2E | 0.00 | | | | | 0.00 | | | 5.08 | 1.08 | 1 | 1 |
| Q63HN8 | E3 ubiquitin-protein ligase | 6.25 | 0.42 | 1 | 1 | 8.61 | 0.61 | 2 | 2 | 4.68 | 0.33 | 1 | 1 |
| Q9NSI6 | Bromodomain and WD re | 4.80 | 1.03 | 1 | 1 | | 0.00 | | | 4.97 | 1.03 | 1 | 1 |
| Q7Z4S6 | Kinesin-like protein KIF21A OS=Homo | 0.00 | | | | 4.46 | 1.02 | 1 | 1 | | 0.00 | | |
| Q5VU43 | Myomegalin OS=Homo sa | 4.83 | 1.02 | 1 | 1 | | 0.00 | | | | 0.00 | | |
| A6NMZ7 | Collagen alpha-6(VI) chai | 4.29 | 1.02 | 1 | 1 | | 0.00 | | | | 0.00 | | |
| Q99250 | Sodium channel protein type 2 subuni | 0.00 | | | | | 0.00 | | | 4.15 | 1.00 | 1 | 1 |
| Q9P260 | LisH domain and HEAT repeat-contain | 0.00 | | | | | 0.00 | | | 4.18 | 0.99 | 1 | 1 |
| Q8WWQ0 | PH-interacting protein OS | 4.90 | 0.99 | 1 | 1 | | 0.00 | | | 4.52 | 0.99 | 1 | 1 |
| O15031 | Plexin-B2 OS=Homo sapi | 4.42 | 0.98 | 1 | 1 | | 0.00 | | | | 0.00 | | |
| Q9P1Z9 | Coiled-coil domain-contai | 4.00 | 0.91 | 1 | 1 | | 0.00 | | | | 0.00 | | |
| Q9H583 | HEAT repeat-containing p | 4.20 | 0.89 | 1 | 1 | | 0.00 | | | | 0.00 | | |
| P49792 | E3 SUMO-protein ligase R | 3.84 | 0.40 | 1 | 1 | | 0.00 | | | 4.33 | 0.47 | 1 | 1 |
| Q7Z6E9 | E3 ubiquitin-protein ligase | 3.85 | 0.84 | 1 | 1 | | 0.00 | | | | 0.00 | | |
| O75165 | DnaJ homolog subfamily C member 13 | 0.00 | | | | | 0.00 | | | 4.37 | 0.80 | 1 | 1 |
| Q8IWZ3 | Ankyrin repeat and KH domain-contair | 0.00 | | | | | 0.00 | | | 9.16 | 0.79 | 1 | 2 |
| P42694 | Probable helicase with zir | 3.83 | 0.77 | 1 | 1 | | 0.00 | | | | 0.00 | | |
| P12259 | Coagulation factor V OS= | 5.51 | 0.76 | 1 | 1 | 5.38 | 0.76 | 1 | 1 | | 0.00 | | |
| Q4AC94 | C2 domain-containing protein 3 OS=H | 0.00 | | | | 3.94 | 0.76 | 1 | 1 | | 0.00 | | |
| Q8TEM1 | Nuclear pore membrane g | 4.12 | 0.74 | 1 | 1 | | 0.00 | | | | 0.00 | | |
| Q15154 | Pericentriolar material 1 protein OS=H | 0.00 | | | | 4.00 | 0.74 | 1 | 1 | | 0.00 | | |
| Q96L96 | Alpha-protein kinase 3 OS | 3.92 | 0.73 | 1 | 1 | | 0.00 | | | | 0.00 | | |
| P49750 | YLP motif-containing protein 1 OS=Ho | 0.00 | | | | | 0.00 | | | 4.25 | 0.72 | 1 | 1 |
| P42345 | Serine/threonine-protein | 4.27 | 0.71 | 1 | 1 | | 0.00 | | | | 0.00 | | |
| Q9UQ35 | Serine/arginine repetitive matrix prote | 0.00 | | | | | 0.00 | | | 4.63 | 0.65 | 1 | 1 |
| Q9NYQ7 | Cadherin EGF LAG seven-pass G-type | 0.00 | | | | | 0.00 | | | 4.66 | 0.63 | 1 | 1 |
| O60229 | Kalirin OS=Homo sapiens GN=KALRN | 0.00 | | | | 3.93 | 0.60 | 1 | 1 | | 0.00 | | |
| Q9NZJ4 | Sacsin OS=Homo sapiens | 5.37 | 0.59 | 1 | 1 | | 0.00 | | | | 0.00 | | |
| Q12955 | Ankyrin-3 OS=Homo sapiens GN=ANK | 0.00 | | | | | 0.00 | | | 5.31 | 0.59 | 1 | 1 |
| P27708 | CAD protein OS=Homo sa | 4.04 | 0.58 | 1 | 1 | | 0.00 | | | | 0.00 | | |
| P18583 | Protein SON OS=Homo sapiens GN=S | 0.00 | | | | 4.23 | 0.58 | 1 | 1 | | 0.00 | | |
| Q14789 | Golgin subfamily B memb | 5.44 | 0.55 | 1 | 1 | | 0.00 | | | | 0.00 | | |
| A2VEC9 | SCO-spondin OS=Homo s | 4.90 | 0.52 | 1 | 1 | | 0.00 | | | | 0.00 | | |
| Q96DT5 | Dynein heavy chain 11, axonemal OS= | 0.00 | | | | 4.73 | 0.49 | 1 | 1 | | 0.00 | | |
| P13611 | Versican core protein OS= | 4.07 | 0.47 | 1 | 1 | | 0.00 | | | | 0.00 | | |

| | | | | | | | | | | | | | |
|---|---|---|---|---|---|---|---|---|---|---|---|---|---|
| A5YKK6 | CCR4-NOT transcription c | 3.90 | 0.42 | 1 | 1 | | 0.00 | | | | 0.00 | | |
| Q07954 | Prolow-density lipoprotein receptor-rel | | 0.00 | | | 4.04 | 0.37 | 1 | 1 | | 0.00 | | |
| Q9UPN3 | Microtubule-actin cross-lin | 3.83 | 0.22 | 1 | 1 | | 0.00 | | | | 0.00 | | |
| Q8WZ42 | Titin OS=Homo sapiens C | 3.89 | 0.06 | 1 | 1 | | 0.00 | | | | 0.00 | | |
| | | | | | 3031 | | | | 2454 | | | | 2769 |
