# Supplementary material for: Microfluidic reactors for advancing the MS analysis of fast biological responses
Source: Microsyst Nanoeng. 2019 Feb 11;5:7. doi: 10.1038/s41378-019-0048-3 (PMC6369226; doi:10.1038/s41378-019-0048-3)
Supplement: Supplementary file 5 — Supplemental table 2 [file 41378_2019_48_MOESM5_ESM.pdf]

ID	Protein description	# AAs	# PSM A (normalized)	# PSM B (normalized)	# PSM C (normalized)	# Average	RSD, %	Type of control
P60709	Actin B Homo sapiens	375	21	15	29	21.7	32.4	Endogenous control
P04406	Glyceraldehyde-3-phosphate dehydrogenase (GAPDH) - Homo	335	50	40	47	45.7	11.2	Endogenous control
P07437	Tubulin beta chain (TBB5) - Homo sapiens	444	8	8	8	8.0	0.0	Endogenous control
P14618	Pyruvate kinase PKM - Homo sapiens	531	15	10	14	13.0	20.4	Endogenous control
P19338	Nucleolin - Homo sapiens	710	8	9	7	8.0	12.5	Endogenous control
P01966	Hemoglobin subunit alpha - Bos taurus	141	6	8	9	7.7	19.9	Standard protein spike
P00921	Carbonic anhydrase 2 - Bos taurus	259	15	16	11	14.0	18.9	Standard protein spike
P02070	Hemoglobin subunit beta - Bos taurus	145	21	27	25	24.3	12.6	Standard protein spike
P02662	Alpha-S1-casein - Bos taurus	214	13	10	7	10.0	30.0	Standard protein spike
P02666	Beta-casein - Bos taurus	224	4	6	4	4.7	24.7	Standard protein spike
P12763	Alpha-2-HS-glycoprotein - Bos taurus	359	93	123	112	109.3	13.9	Standard protein spike
P14618	Pyruvate kinase PKM (GADFLVTEVENGGSLGSK)	18	44	35	49	42.7	16.6	Peptide spike
P14618	Pyruvate kinase PKM (LAPITSDPTEATAVGAVEASFK)	22	69	64	68	67.0	3.9	Peptide spike
